# Endophytic Fungi as a Source of Antibacterial Compounds—A Focus on Gram-Negative Bacteria

**DOI:** 10.3390/antibiotics11111509

**Published:** 2022-10-29

**Authors:** Dayse Pereira Dias Silva, Macley Silva Cardoso, Alexandre José Macedo

**Affiliations:** Laboratório de Diversidade Microbiana (LabDiM), Centro de Biotecnologia, Faculdade de Farmácia, Universidade Federal do Rio Grande do Sul, Av. Bento Gonçalves, 9500, Porto Alegre 91501-970, Brazil

**Keywords:** antibacterial activity, endophytic fungi, metabolites, gram-negative bacteria

## Abstract

Bacterial resistance has become one of the main motives in the worldwide race for undescribed antibacterial agents. The difficulties in the treatment of bacterial infections are a public health issue that increasingly highlights the need for antimicrobial agents. Endophytic microorganisms are a promising alternative in the search for drugs, due to the vast number of metabolites produced with unique characteristics and bioactive potential. This review highlights the importance of endophytic microorganisms as a source of secondary metabolites in the search for active molecules against bacteria of medical importance, with a special focus on gram-negative species. This fact is supported by the findings raised in this review, which brings an arsenal of 166 molecules with characterized chemical structures and their antibacterial activities. In addition, the low cost, ease of maintenance, and optimization-controlled fermentation conditions favor reproducibility in commercial scale. Given their importance, it is necessary to intensify the search for new molecules from endophytic microorganisms, and to increasingly invest in this very promising font.

## 1. Introduction

The development of antibiotic drugs has initially created a false sense of security, implying that the problem of infectious diseases would be definitively solved. Over the years, however, this scenario has changed and infectious diseases have come to be feared again [1]. In addition to the microbial pathogens for which there is no current treatment option, the emergence of multidrug resistant organisms further compromises the use of antibiotic drugs. Microbial resistance has been long known to create pitfalls in the treatment of several diseases caused by bacteria, viruses, fungi, and parasites, decreasing the effectiveness of the therapy. The adaptability and resistance of infectious agents to antibiotics is incredibly fast-moving [2]. For example, sulfonamide-resistant organisms, such as *Streptococcus pyrogenes*, emerged shortly after the introduction of the first sulfonamide drug, the azo compound “Prontosil,” developed in the 1930s as a solution for gram-negative and gram-positive antimicrobial therapy [3].

Bacterial resistance has, thus, become one of the main factors in triggering a global, urgent search for novel antimicrobial agents. Resistance associated with known pathogens such as methicillin-resistant *Staphylococcus aureus* (MRSA), respiratory pathogens such as *Streptococcus pneumoniae*, and gram-negative pathogens, is increasingly often described in epidemiological analyses, impacting all fields of medicine and representing a threat to public health [4]. A list of priorities for antibiotic-resistant bacteria has been created by the World Health Organization (WHO) to guide research and development of new antibiotics. Critical priority organisms include carbapenem-resistant *Acinetobacter baumannii*, carbapenem-resistant *Pseudomonas aeruginosa*, and carbapenem-resistant *Enterobacteriaceae* and 3rd generation cephalosporin. The high priority group comprises *Enterococcus faecium*, *Staphylococcus aureus*, *Helicobacter pylori*, *Campylobacter*, *Salmonella* spp., and *Neisseria gonorrhoeae*, while *Streptococcus pneumoniae*, *Haemophilius influenzae*, and *Shigella* spp. are considered medium priority [5].

Infections by resistant microbes are often associated with hospital environments due to clinical routine, but they also occur in the community and are a consequence of selective pressure from antibiotic use in humans, and animals. The environment’s accumulation of antimicrobials also contributes to the increase of AMR (Antimicrobial Resistance) [6,7,8]. Environmental antimicrobial resistance is also associated with urban water pollution, as in the Hua Krabue canal in central Thailand. Asia has an important relationship with aquaculture, accounting for 91% of global aquaculture production, so the rapid growth of this sector has modified and destabilized several ecosystems. This has caused vulnerability and pollution of these waters and, consequently, the spread of diseases has led to the uncontrolled use of antibiotics. On the part of these producers, the impact of this is the risk to human health and the contribution to antimicrobial resistance [9].

Agriculture contributes significantly to AMR in humans; in the United States, 80% of the antibiotics sold are added to animal feed as a way to prevent future diseases, in addition to being used to treat sick animals. Around 63 thousand tons of antibiotics have been used in livestock production worldwide [10]. In the last decades, the ease of international travel has also contributed to the global problem of AMR; approximately 30% of international travelers acquire extended-spectrum ß-lactamase producing *Escherichia coli* (ESBL-PE). These are often resistant to various antimicrobials, so these trips have a role in the spread of resistant genes and organisms [7,10].

On the other hand, attention has been paid to antimicrobial susceptibility testing. From a clinical point of view, these trials are intended to assess the effectiveness or failure of potential antimicrobial candidates. However, evidence has been raised about the ability of these tests to select clinically effective antimicrobials, considering the differences observed between in vitro results and therapeutic failure in patients submitted to the drugs subsequently selected. [11,12]. Another reason is based on regulatory issues regarding the use of antibiotics by the world population. While some Western countries still use antibiotics such as ciprofloxacin against *Salmonella typhi* in therapeutics, South African countries deal with ciprofloxacin-resistant strains and employ azithromycin and ceftriaxone as first-line antimicrobial treatment [13]. The acquisition of antibiotics without a prescription demonstrates a challenging reality in the fight against resistant pathogens [14].

Microbial resistance arises through several mechanisms, including resistance genes that evolve over time in response to selective pressure exerted by the inappropriate use of antibiotics, and the lack of new antimicrobial drugs in the current scenario [15]. These resistance genes are transferred from one bacterium to another by horizontal transfer in the form of plasmids, transposons, integrons, or bacteriophages [2,8,16,17]. Environmental contamination by pharmaceutical compounds, in particular antibiotics, plays a major role in the spread of microbial resistance. Because wastewater treatment is not fully effective in removing contaminants, drinking water systems are vulnerable, exposing several environments and organisms to antibiotics and resistance genes [16].

The impact generated by microbial resistance is immeasurable, leading to prolonged disease and increased mortality, in addition to the social and human impact and the rise in costs for the health sector. The problem of antimicrobial resistance is further aggravated due to the inappropriate and exacerbated use of antibiotics worldwide, the lack of new antimicrobials, and the lack of investments in the search for new antimicrobials, which also contribute to this sad scenario [18]. In the last 20 years, only two new classes of antibiotics, lipopeptides and oxazolidinones, were approved by the international drug agencies US Food and Drug Administration (FDA) and European Medicines Agency (EMA). Both classes are active against gram-positive bacteria. However, when it comes to gram-negative bacteria, the quinolone class discovered in 1962 was the last class of drug that was active against gram-negative bacteria [19].

By 2021, the current clinical antibacterial pipeline has listed 45 traditional antibiotics, of which 27 have been reported to be active against the priority pathogens listed [20]. The last antibacterial agent reported is cefiderocol, active against gram-negative bacteria, including the critical bacteria listed by the WHO, namely *Acinetobacter baumannii*, *Pseudomonas aeruginosa*, and Enterobacteriaceae. Cefiderocol has a chemical structure similar to ceftazidime and cepefime, developed to treat various bacteria resistant to β-lactams and carbapenems [21].

The associated healthcare costs are extremely high, reaching 20 billion dollars per year in the United States [22]. It is estimated that more than 2.8 million infections resistant to antibiotics were registered in the US, and more than 35 thousand deaths per year occurred as a result of these infections [23]. If no effective solution is found to slow the growth of microbial resistance, it is estimated that by 2050, 10 million lives will be affected and will be at risk [8]. Although accurate estimates of costs related to microbial resistance are difficult to make, a loss of USD 100 trillion in the GDP is predicted by 2050 if no action is taken against AMR [24].

## 2. Natural Products from Endophytic Microorganisms as a Resource in the Search for Bioactive Molecules

Accelerated microbial resistance to drugs, recurrent diseases, and new cases of life-threatening infections make us more and more in need of new therapeutic compounds [25]. The world population has long treated diseases using natural products [26]. The isolation and elucidation of several compounds, among which taxol is a prime example, was the starting point in the search for new therapies [27]. Specialized metabolites are produced in response to external environmental changes, such as nutritional changes, infections, and competition, and have proven to be a promising option in the search for new drugs [28,29].

Almost one fifth of all new drugs approved between 1981 and 2019 were derived from natural products. Bringing these data with a focus on antibacterial agents, of the new drugs approved between 2015 and 2019, 48% of them came from natural products and their derivatives [30]. By joining forces, biology and synthetic chemistry create great potential for discovery of new structures that can serve as effective drugs against a variety of diseases. Microorganisms are promising and reproducible sources of bioactive chemical potential when compared to other types of natural sources, such as plants and animals [31]. These organisms (fungi and bacteria, among others) play a fundamental role in the production of new natural products [32].

Endophytes (from the Latin words endo = inside, and phytos = plant) are endosymbiont organisms (mostly fungi or bacteria) that live within plant tissues. The term endophytic fungi was coined by De Bary [33], who distinguished them from pathogenic fungi for being asymptomatic, i.e., not causing any harm to the plant host [34].

Endophytes are present in plants, more precisely, in their roots, seeds, stems, and leaves through a symbiotic association involving mutualism or an association involving parasitism. In mutualistic interactions, microorganisms are able to produce beneficial metabolites to plants, promoting resistance to biotic (phytopathogens and herbivores) and abiotic (pollution, temperature, radiation, etc.) stresses. In this association, the host plant protects and makes available the exudate produced by its roots as a source of energy for the endophytic, which, in response, produces metabolites that promote plant growth and confer resistance to external conditions. In parasitism, the endophytic starts to exert pathogenic action on its host, as is observed in some varieties of rice, tomato, wheat, soybean, cotton, citrus, and others [35,36,37]. The endophytic can co-exist and establish a type of relation of neutralism. It can also assume a quiescent state, that is, it remains dormant for a long period of time without expressing any manifestation in the host, until changes occur in environmental conditions and these are favorable to the endophytic [38].

Endophytic microorganisms can influence several characteristics of the plant as well as perform important functions for their well-being, acting as protection against pathogens and herbivores [39]. Some mechanisms are employed by endophytes to provide protection to the plant; these mechanisms include direct and indirect actions. The direct mechanism involves the production of antibiotics that will help in the elimination of pathogens; secretion of enzymes such as chitinases, hemicellulases, and cellulases; production of phytohormones, such as gibberellic acid, that will help in the growth of the plant, making this attribute interesting for agricultural area; production of siderophores as important iron chelators; production of indole compounds, etc. As a consequence, in the indirect mechanisms, endophytes induce plant resistance, and there is a stimulus in the production of secondary metabolites, promotion of plant growth, and regulation of plant physiology [40].

Currently, it is known that endophytes are capable of producing toxins, antibiotics, and many other products of biotechnological interest, in addition to promoting plant protection [32]. The potential of endophytic microorganisms to produce a range of secondary metabolites makes them an interesting source in the search for new antimicrobial agents, and their application in the food and cosmetics industry can contribute to several biotechnological applications. Some of the metabolites produced by endophytes are terpenes, alkaloids, phenols, tannins, quinones, saponins, steroids, etc. [38].

Some factors, such as climatic conditions, geographic location, soil conditions in which the endophytic is inserted, and season of the year in which the sample was collected, contribute to the production of differentiated secondary metabolites. It is believed that endophytes produce metabolites similar to those produced by the host plant, and this characteristic makes them quite attractive. This is because during the fermentation process, these microorganisms manage to produce the metabolite of interest in an optimized way and on a large scale. In this way, they have the advantage of being ecologically correct, because the raw material used for fermentation in the laboratory to obtain the metabolite of interest is much smaller when compared to the plant raw material of the plant, which demands a very large amount, making the process ecologically incorrect [41].

Virtually all plant species studied to date presented endophytic microorganisms, fungi, or bacteria which are present in most different tissues and plant organs. It is believed that many of the medicinal properties of certain plants may be related to the metabolites produced by endophytes [31,32]. Endophytic microorganisms have become an increasingly appealing research object, especially the ones from plants already used in traditional medicine for their therapeutic properties [37].

### 2.1. Mechanisms of Action Antimicrobial

**Figure 1:**
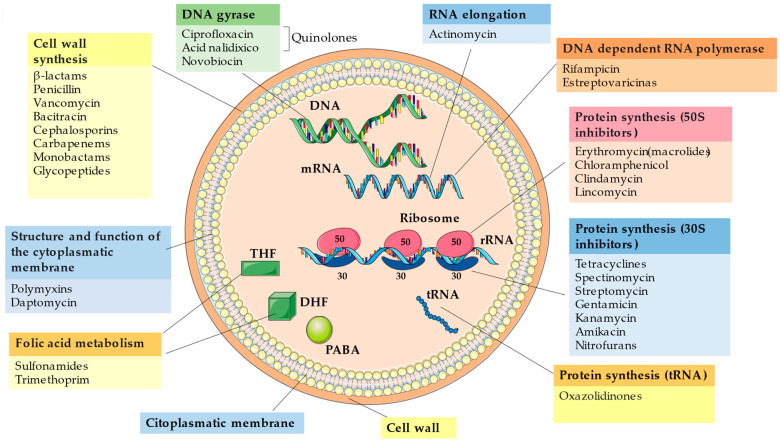
Mechanisms of action antimicrobial. The authors (2022) adapted from [42].

Faced with the search for bioactive molecules to combat antimicrobial resistance that often seems endless due to the impressive ability of these bacteria to adapt and mutate, it is first necessary to understand how the mechanisms of antimicrobial action occur and invest in the elucidation of these mechanisms. Figure 1 shows the main mechanisms of action by which an antibiotic can act, either by destroying or inhibiting the microorganism. The mechanisms of action of an antimicrobial can happen in several ways: through its interaction with the cell wall, through the plasma membrane, in protein synthesis, causing inhibitions in the synthesis of DNA and RNA, and interfering with the metabolism of folic acid.

PABA = para-aminobenzoic acid; DHF = dihydrofolate; THF = tetrahydrofolate; DNA = deoxyribonucleic acid; mRNA = messenger ribonucleic acid; tRNA = transporter ribonucleic acid; rRNA = ribosomal ribonucleic acid.

#### 2.1.1. Mechanisms of Action in the Cell Membrane

The plasma membrane consists of fatty acids, phospholipids, polysaccharides, and proteins, and is responsible for controlling the transport of molecules and ions through a selective permeability performed by transmembrane proteins, such as porins, which form channels allowing the passage of molecules such as peptides. amino acids, nucleotides, vitamins, etc. [43]. When this membrane is interfered with by antimicrobial agents, selective permeability is altered and the cell’s ionic gradient is lost, resulting in cell damage and cell death [42,44]. In this type of mechanism, the antimicrobial seeks to interfere with the synthesis of fatty acids and phospholipids in the membrane. An example of an antimicrobial that acts in this pathway is Polymyxin B, which affects the permeability of the bacterial membrane, facilitating the uncontrolled movement of ions through it. Daptomycin is a lipopeptide that acts by interacting with membrane phospholipids, depolarizing it and interrupting the membrane potential, releasing ions from the cytoplasm to the extracellular matrix [42]. This mechanism occurs in *Pseudomonas aeruginosa* treated with polymyxin B [45].

#### 2.1.2. Mechanisms of Action in the Cell Wall

The cell wall is made up of peptidoglycan forming a rigid and thick wall; the wall of gram-negative bacteria has only a thin layer of peptidoglycan, and it is through this layer that the non-specific diffusion of hydrophilic solutes occurs, through porins. Antimicrobial agents target cell wall synthesis, while β-thalactams, penicillins, vancomycins, bacitracins, caphalosporins, glycopeptides, and carbapenems act on the cell wall by crossing porins present in the outer membrane and binding to protein receptors of the type penicillin-binding proteins (PBP), thus inactivating them and interrupting the final step of cell wall synthesis. In gram-negative bacteria, this process is difficult because the antibiotic needs to cross the cell membrane. The opposite happens in gram-positive bacteria because the peptidoglycan polymer is closer to the surface, facilitating the binding of the antibiotic with the PBP [44].

Penicillin, like other β-lactam antibiotics, has bactericidal power. Penicillin G is the only natural penicillin administered clinically; its derivative penicillin V is more stable when administered orally, but both forms have limited activity against gram-negative bacteria, as these bacteria produce β-lactamases hydrolyzing enzymes that are synthesized by gram-positive and gram-negative bacteria, and these enzymes degrade a portion of the β-lactam ring of β-lactam antibiotics. Gram-negative species such as *Klebsiela pneumoniae* and *Escherichia coli* produce broad-spectrum β-lactamases (ESBL) by degrading the β-lactam rings of the antibiotics ceftazidime [42]. Penicillin and cephalosporin act in the last step of peptidoglycan formation as an analogue of the D-alanine-D-alanine moiety. In this way, the enzymes involved in the transpeptidization reaction react with the β-lactams that block the transpeptidase reaction. The bacteria is then killed by inhibiting cell wall peptidoglycan synthesis [42,46].

#### 2.1.3. Mechanisms of the Action in the Protein Synthesis

In the mechanisms of action through protein synthesis, antimicrobial agents such as tetracyclines, erythromycin, streptomycin, among others, target ribosomes. Ribosomes have two subunits, one consisting of proteins and the other of a type of RNA called ribosomal RNA (rRNA). The subunits of a 70S ribosome are a smaller 30S unit containing one rRNA molecule, and the other larger 50S subunit containing two rRNA molecules [43]. Bacterial DNA is used to synthesize RNA molecules by a process known as transcription. The ribosomal structure synthesizes proteins present in mRNA by a process called translation [47].

Antibiotics such as tetracyclines and aminoglycosides (streptomycin, gentamicin, etc.) have their mechanism of action through binding with the 30S subunit of the bacterial ribosome, preventing the binding of aminoacyl tRNA in the A site of the ribosomes as well as the addition of amino acids and, consequently, the protein synthesis [48]. In gram-negative bacteria, tetracyclines cross the outer membrane through porin channels, coordinating an ion-tetracycline complex that is attracted by the outer membrane potential and leads to accumulation in the periplasm. There, the complex dissociates and releases uncharged tetracycline into the medium, causing it to diffuse through the inner membrane. Once inside the bacterial cell, tetracycline reversibly binds to the ribosome, which would explain its bacteriostatic power. [49,50].

Inhibitors of the 50S subunit interact with the ribosome by binding to the 23S portion of the rRNA, thus preventing its movement through the mRNA. This happens through the inhibition of peptidyl transferase; as a consequence, the coding of amino acids necessary for protein synthesis does not occur. Chloraphenicol, erythromycin, and lycosamides are examples of antibiotics that bind to the 50S subunit [47]. Oxazolidinones, represented by linezolid, are a class of protein inhibitor that differs from other inhibitors by binding to the 50S subunit and preventing it from binding to the 30S subunit 70S [51].

#### 2.1.4. Mechanisms of Action in the Nucleic Acid Synthesis

During DNA replication, topoisomerases IV (gram-positive) and topoisomerases II enzymes, also called DNA gyrase (gram-negative), play an important role in bacterial DNA replication and transcription, allowing the supercoiling of the DNA molecule [43].

DNA gyrase responsible for opening the DNA strand is made up of multiple subunits that perform crucial ATP-dependent functions in DNA replication. DNA gyrase is composed of two subunits, GyrA and GyrB, and topoisomerase IV has two subunits, ParC/GlrA and ParE/GrlB. These subunits have the function of binding to DNA and hydrolyzing ATP, and both are targets of quinolones and coumarin antibiotics. Without supercoiling, there is no separation of DNA strands and, therefore, there is no transcription and protein synthesis [52].

Quinolones such as nalidixic acid and ciprofloxacin are used in the treatment of *P. aeruginonsa* infections by inhibiting DNA gyrase in the replication fork formation. Some mutations can occur in the GyrA and GyrB genes of the bacterium; when this happens, the bacterium develops resistance to these antibiotics [42].

#### 2.1.5. Mechanisms of Action in the Metabolisms of the Folic Acid

Sulfonamides and trimethoprim act on folic acid metabolism. Sulfonamide inhibits the enzyme dihydropteroate synthase having a greater affinity for that enzyme than for the natural substrate for aminobenzoic acid (PABA), a precursor of tetrahydrofolate acid (THF), which is extremely important in nucleic acid synthesis. On the other hand, trimethoprim acts at a later stage of folic acid synthesis by inhibiting the enzyme dihydrofolate reductase [52].

Sulfonamides and trimethoprim, when combined, act at different stages of the same biosynthetic pathway, showing synergism and a low mutation rate for resistance. In the clinic, sulfonamides are used for the treatment of urinary tract infections caused by *E. coli* [53].

## 3. Natural Products against Gram-Negative Bacteria

The great variability of chemical structures found in natural products makes them extremely attractive leads in the search for novel antimicrobial compounds that might help overcome the problem of resistance. The present review provides a survey of molecules isolated from endophytic fungi with antibacterial activity against Gram-negative bacteria. The search comprised only isolated molecules, excluding data from crude extract. The search was carried out at PUBMED using the following keywords: “Endophytic fungi and antibiotics.” Table 1 presents the data collected from PUBMED searches, encompassing the period from 2012 to 2019.

## 4. Discussion

### 4.1. Acids

The endophytic fungus *Diaporthe phaseolorum* (8S) isolated from the seeds of the Amazonian plant *Paullinia cupana* var. *sorbilis (Mart.)*, popularly known as guarana, was collected in the city of Manaus (2°53′29.14″ S and 59°58′39.90″ W, 99 m high) and Maue’s (3°22′54″ S and 57°42′55″ W, 18 m high), State of Amazonas, Brazil. The plant was collected at the end of the dry season, with a rainfall and temperature, respectively, of 196.0 mm and 28.46 °C in Manaus, and 272.3 mm and 28.17 °C in Maue’s. The authors reported the production of 3-hydroxypropionic acid **(1)** (3-HPA). The compound was evaluated for its antimicrobial activity and showed activity against strains of multi-resistant and sensitive *E. coli* and *P. aeruginosa*. MIC values were >30 µg/mL for *E. coli* strains and 0.23 µg/mL for *P. aeruginosa* strains [54].

### 4.2. Alkaloids

In 2019, Jiang and co-workers [55] described three alkaloids from the endophytic *Fusarium proliferatum* AF-04 (GenBank accession no. MF426031) isolated from green Chinese onion, named indol-3-acetic acid **(2)**, methyl indolyl-3- acetate **(3)**, and bassiatin **(4)**. These alkaloids were active against *E. coli* with MIC above 100 μg/mL.

Pinheiro and co-workers [56] isolated the endophytic fungus *Aspergillus* sp. EJC08 from the plant *Bauhinia guianensis*, collected in Belém-PA city, a typical species from the Amazon Rainforest of the family Leguminosea [94]. From this endophyte, the authors isolated two alkaloids with antibacterial activity, pseurotin A **(5)** and fumigaclavine C **(6)**, both active against *E. coli* and *P. aeruginosa*. The fungus was grown in a rice medium for thirty days, and the alkaloids were obtained by ethyl acetate extraction and successive fractionation followed by purification on a silica gel chromatographic column.

### 4.3. Anthraquinones and Derivatives

Altersolanol B **(7)**, a naturally-occurring reduced anthraquinone [95], was isolated from the endophytic fungus *Phomopsis longicolla* HL-2232 (GenBank accession number KJ466981), obtained from fresh healthy leaves of *Bruguiera sexangular* var. *rhynchopetala* collected from the South China Sea. The compound was active against two strains of *Vibrio*, *V. parahaemolyticus* and *V. anguillarum*, with MIC values of 2.5 and 5.0 µg/mL, respectively [57].

Sun and co-workers [58] described seven anthraquinones from the endophytic fungus *Coniothyrium* sp. (internal strain No. zw86), isolated from the plant *Salsola oppostifolia*, obtained from the island Gomera, in the Canary Islands. The authors evaluated the antibacterial activity of the compounds against *E. coli* using an agar diffusion test and obtained inhibition zones as follows: 11 mm for compounds **(9)** and **(10)**, 15 mm for **(11)**, 7.5 mm for **(12)** and **(14)**, and 6 mm for compounds **(13)** and **(15)**.

The endophytic fungus *Xylaria* sp. JK50 (accession number: JX624289.1), isolated from *Taxus mairei* and belonging to the *Taxaceae* family, produces an anthraquinone derivative with antibacterial activity. The compound nalgiovensin **(11)** showed MIC values of 50 µg/mL against *E. coli* strains [59].

### 4.4. Benzofuranoids

Cai and collaborators [60] isolated two benzofuranoids with antibacterial activity; 2- (hydroxymethyl) -3-propylphenol **(16)** and (-)—brassicadiol **(17)** were isolated from the endophytic fungus *Aspergillus* sp. ZJ-68 found in the leaves of the mangrove plant *Kandelia candel* collected from the Zhanjiang Mangrove Nature Reserve in Guangdong Province, China. The benzofuranoids were active against strains of *E. coli* and *P. aeruginosa* with MIC = 8.3 and >100 µg/mL, respectively, for **(16)**, and MIC = 12.5 and >100 µg/mL for **(17)**.

### 4.5. Benzophenone and Derivatives

Shang and collaborators [61] isolated the endophyte *Nigrospora* sp. MA75 (accession No. GenBank HQ891662), from the steam of the marine plant *Pongamia pinnata*, collected from Guangxi Zhuang Autonomous Region of China. After fermentation and ethyl acetate extraction, it was possible to obtain the benzophenone griseophenone C **(18**). The compound is active against *E. coli*, *P. aeruginosa* and *P. fluorescens*, with MIC values of 2, 0.5, and 0.5 µg/mL, respectively.

Liu and co-workers [62] described four compounds derived from benzophenone, cytosporins A-D **(19–22)**, found in the endophytic fungus *Cytospora rhizophorae* A761 isolated from a traditional Chinese medicine plant, *Morinda officinalis How* (family Rubiaceae). They are comprised of phenolic groups conjugated with hemiterpenes, being the first example of a natural meroterpenoid with a structure that incorporates a hemiterpene and a benzophenone portion. The compounds were evaluated in vitro for antimicrobial activity, all presenting an MIC of 250 µg/mL for *E. coli*.

### 4.6. Benzopyrones

Benzopyrones was isolated from the endophytic *Alternaria tenuissima* SP-07 from the roots of *Salvia przewalskii* Maxim of the family Lamiaceae, collected from Longxi County, Gansu Province of China. The genus *Alternaria* is a type of ascomycete and the most well-known plant pathogen. From the ethyl acetate extract, it was possible to obtain the benzopyrones alternariol **(23)**, alternariol methyl ether **(24)**, and altenuene **(25)**. These all possessed antibacterial activity against *E. coli*, showing MIC of 100 µg/mL, >100 µg/mL, and 100 µg/mL for the respective compounds [63].

### 4.7. Biphenyl Derivative

An undescribed biphenyl derivative, 5,5′-dimethoxybiphenyl-2,2′-diol **(26)**, was isolated from the endophytic fungus *Phomopsis longicolla* HL-2232, obtained from the fresh healthy leaf of *Bruguiera sexangula* var. *rhynchopetala*, collected in southern China. The compound was evaluated for its antibacterial potential, and exhibited activity against *Vibrio parahaemolyticus*, with MIC of 10 µg/mL [57].

### 4.8. Butyrolactone Derivative

The endophytic fungus *Aspergillus flavipes* Y-62, isolated from the stems of the plant *Suaeda glauca* (Bunge) collected in the Zhoushan coast, Zhejiang province, East China, produced a butyrolactone derivative, 2-O-methylbutyrolactone **(27)**, with antibacterial activity. The compound showed MIC values of 32 µg/mL for strains of *E. coli* and *K. pneumoniae*, and MIC of 64 µg/mL for *P. aeruginosa* [64].

### 4.9. Carboxamides

Two undescribed bioactive carboxamides, called vochysiamide A **(28)** and B **(29)**, were isolated and evaluated for their antimicrobial activity. The two compounds showed MIC of 1 mg/mL and 0.08 mg/mL, respectively, against *Klebsiella pneumoniae* carbapenemase-producing strain (KPC). The compounds were isolated from the endophytic fungus *Diaporthe vochysiae* LGMF1583, which was obtained from the leaves of the medicinal plant *Vochysia divergens*, popularly known as Cambará, and was collected in Corumbá in the region of the wetland of the Mato Grosso do Sul 19°32′36.9″ S, 57°02′21.8″ W [65]. Carbapenemases are enzymes with hydrolytic activity that bind and deactivate their targets, the beta-lactams. They are considered a major problem with microbial resistance in human pathogens, and have worsened due to their resistance and cross-resistance mechanisms compared to other antimicrobials [96,97].

For a long time, carbapenems have been used successfully to treat infections caused by enterobacteriaceae, such as *E. coli* and *K. pneumoniae*. However, carbapenem-producing enterobacteriaceae, known as carbapenem-resistant enterobacteriaceae (CRE), have emerged, and they present broad resistance to most ß-lactam antibiotics, including “last line” carbapenems [98].

### 4.10. Quinone Derivative

A quinone derivative called 2,3-didehydro-19ahydroxy 14-epicochlioquinone B **(30)** was reported by Shang and co-workers [61]. It was isolated from the endophytic fungus *Nigrospora* sp. MA75 (GenBank accession No. HQ891662), obtained from the marine semi-mangrove plant *Pongamia pinnata*, collected from Guangxi Zhuang Autonomous Region of China. The compound showed potent antibacterial activity against *E. coli*, *P. aeruginosa*, and *P. fluorescens* strains, with MIC of 4.0, 4.0, and 0.5 µg/mL, respectively. This compound showed results superior to the positive control Ampicillin for *E. coli* and *P. fluorescens* (control MIC values of 8 and 4 µg/mL, respectively).

### 4.11. Cyclic Peptide

Ma and co-workers [66] isolated one cyclic peptide, malformin E **(31)** of the endophytic fungus *Aspergillus tamarii* FR02, isolated from the roots of the *Ficus carica* that was collected from Qinling Mountain in China’s Shaanxi province. The compound was active against *C. albicans, E. coli*, and *P. aeruginosa*, with MIC of 7.24, 0.91, and 1.82 µM, respectively.

### 4.12. Terpenes

Five cyclopiane-type diterpenes, Leptosphin C **(32)**, conidiogenone F **(33)**, conidiogenone C **(34)**, conidiogenone D **(35)**, and conidiogenone G **(36)**, were identified in the solid state fermentation culture of the endophytic fungus *Leptosphaeria* sp. XL026 (GenBank accession no. MK603060) isolated from the leaves of *Panax notoginseng*, which were collected from Shijiazhuang, Hebei province, P. R. China. These molecules showed antibacterial activity against *E. coli*, *P. aeruginosa*, and *Salmonella typhimurium* strains, with MIC ranging from 12.5 to >100 µg/mL. The cyclopian-type diterpene conidiogenone D **(35)** is noteworthy for having a MIC value of 12.5 µg/mL for *P. aeruginosa* [67], a species that is among the main gram-negative bacteria present in hospital infections [99].

### 4.13. Cytochalasan-Type Alkaloids

Cytochalasan-type alkaloids were isolated from the endophytic fungus *Chaetomium globosum* NM0066 obtained from the leaves of *Ginkgo biloba* plant. The cultivation of the fungus in a Sabouraud medium produced a new cytochalasan alkaloid, chaetoglobosin Vb **(37)**, and two other known alkaloids, chaetoglobosins V **(38)** and G **(39)**. Chaetoglobosin Vb was active against strains of *E. coli* and *P. aeruginosa* with MIC above 100 μg/mL, while the alkaloids chaetoglobosins V and chaetoglobosins G both had MIC >100 and 50 μg/mL for *E. coli* and *P. aeruginosa*, respectively [68]. Chaetomium is a genus of fungi of the family Chaetomiaceae, subdivision Ascomycota, with more than one hundred species in terrestrial and marine environments. It has been widely reported in the production of chaetoglobosin, and has been shown to be a rich source of natural products with complex structures, with more than 200 isolated compounds of this genus already reported. Among them, in addition to chaetoglobosins, are other molecules such as epipolythiodioxopiperazines, azaphilones, depsidones, xanthones, anthraquinones, chromones, terpenoids, and steroids [100].

### 4.14. Cytochalasins and Derivatives

The cytochalasin is a family of specialized metabolites of fungi with more than 80 members isolated from various species of fungi endophytic, including *Aspergillus*, *Rhinocladiella*, and *Xylaria* spp. [101,102,103]. This group, due to its unique structures, exhibits a wide spectrum of biological activities, such as antibacterial [64] and cytotoxic [101]. They present an isoindolone ring highly substituted at C-3 by a benzyl group, being fused to a macrocyclic ring of 11 to 14 members [102].

Yang and collaborators [69] described cytochalasin 11 -cytochalasa-5 (6), 13-diene-1,21-dione-7,18-dihydroxy-16,18-dimethyl 10-phenyl (7S *, 13E, 16S *, 18R *) **(40)** from the endophytic fungus *Daldinia eschscholtzii* HJ001 (GenBank accession no. KY440188), isolated from a mangrove (*Bruguiera sexangula var. rhynchopetala)* in the South China Sea. This metabolite was active against *E. coli* and two strains of *Vibrio parahaemolyticus* and *V. alginolyticus*, with a MIC of 50 μg/mL.

Jouda and co-workers [70] isolated from the endophytic fungus *Phomopsis* sp. CAM240, obtained from the nut of the Garcinia kola (family Clusiaceae) bought at the Mokolo local market in Yaounde (Cameroon), three cytochalasins, 18-metoxycytochalasin J **(41)**, cytochalasins H **(42)**, and cytochalasins J **(43)**. The compounds were active against *Vibrio cholerae NB2, Vibrio cholerae PC2*, and *Shigella flexneri SDINT.* Compounds **(41)** and **(43)** exhibited MIC of 512 µg/mL for *V. cholerae* NB2, >512 µg/mL for *V. cholerae* PC2, and 128 µg/mL for *S. flexneri* SDINT. Compound **(42)**, in turn, exhibited MIC of 512, 256, and 128 µg/mL, respectively, for the three strains.

Eight cytochalasin derivatives were isolated from the endophytic fungus *Aspergillus flavipes* Y-62, obtained from the Suaeda glauca (Bunge) stems collected along the Zhoushan coast, Zhejiang province, East China. These are cytochalasan Z16 **(44)**, Z7 **(45)**, Z17 **(46)**, rosellichalasin **(47)**, Z13 **(48)**, dipeptide aspergillazine A **(49)**, flavipin **(50)**, and N-benzoyl-L phenyalaninol **(51)**. These compounds were active against the pathogens *E. coli*, *K. pneumoniae*, and *P. aeruginosa*. The compound Z7 **(45)** was the most active against *E. coli*, with a MIC of 16 μg/mL, while the other compounds had MIC between 32 and 128 μg/mL [64].

### 4.15. Decalin Polyketides

Two decalin polyketides were described from the endophytic fungus *Eupenicillium* sp. LG41, isolated from the roots of *Xanthium sibiricum* (Asteraceae), a medicinal Chinese plant collected in Taian, Republic of China. The compounds eupenicinicol A **(52)** and eupenicinicol B **(53)** were evaluated for their antibacterial activity. Compound **(52)** was active against strains of *Acinetobacter* sp. and *E. coli*, showing MIC of 10 and 5.0 µg/mL, respectively. Compound **(53)** showed MIC >10 and 5.0 µg/mL against *Acinetobacter* sp. and *E. coli* [71].

### 4.16. Depsipeptide

Jiang and co-workers [55] characterized the depsipeptide from the fungus *Fusarium proliferatum* AF-04 (GenBank accession no. MF426031) isolated from green Chinese onions. The compound Depsipeptide Beuvarecinin **(54)** showed antibacterial activity against *E. coli*, exhibiting MIC above 100 µg/mL.

### 4.17. Desmethyl Fusarin C Derivatives

Kyekyeku and collaborators [72] isolated seven desmethyl derivatives from Fusarin C from the endophytic fungus *Fusarium solani* JK10. The fungus was obtained from the roots of *Chlorophora regia* (Moraceae) obtained from the Asakraka forest in the region of Ghana (6°37′48.39″ N 0°41′6.87″ W), is an important medicinal plant in Africa. All compounds were active against strains of *Acinetobacter* sp. and *E. coli*. The Compounds **(55)**, **(56)**, **(59)**, and **(61)** showed MIC >10 µg/mL for *Acinetobacter* sp., while compounds **(57)**, **(58)** and **(60)** presented MIC of 10 µg/mL, a marked activity compared to the reference streptomycin. Against *E. coli*, compounds **(55)**, **(56)**, **(57)**, **(58)** and **(60)** presented MIC of 10 µg/mL. Compounds **(59)** and **(61)**, on the other hand, showed a higher activity against *E. coli*, with an MIC of 5 µg/mL.

### 4.18. Diketopiperazine and Epipolythiodioxopiperazine

The compound diketopiperazine bionectin D **(62)**, a rare dioxopiperazine with a methylthio substitution on α carbon of a cyclized amino acid residue, and bionectin E **(63)**, also a diketopiperazine, were isolated from the endophytic fungus *Bionectria* sp. Y1085 (GenBank accession number MH429795.1), from the plant *Huperzia serrata*, which was collected in Yunnan province in China. Compound **(62)** was active against *E. coli* and *Salmonella typhimurium* with MIC of 25 µg/mL for both strains. On the other hand, compound **(63)** showed MIC of 12.5 µg/mL for both strains [73].

Three diketopiperazines were obtained from the ethyl acetate extract of the endophytic fungus *Bionectria* sp. Y1085 was isolated from *Huperzia serrata*, verticillin A **(64)**, sch 52901 **(65)**, and gliocladicillin C **(66).** The three compounds were active against *E. coli* and *S. typhimurium* strains. Compound **(64)** showed MIC of 12.5 µg/mL for both strains and compounds **(65)**, and **(66)** exhibited MIC of 6.25 µg/mL for the two strains tested [73].

An ETP not described, called 6-formamide chetomin **(67)**, was isolated from the endophytic fungus *Chaetomium* sp. M336, isolated from the *Huperzia serrata* (Thunb. ex Murray) Trev. Plant, which was collected in Xichou, Yunnan province in China. Compound **(67)** was suggested to have a promising antibacterial potential, with an MIC of 0.78 µg/mL against strains of *E. coli* and *Salmonella thyphimurium* [74].

### 4.19. Ergosterol Derivatives

Ergosterol is a sterol that plays a key role in fungal membranes. Some of its functions are controlling the fluidity and permeability of the membrane, and in some plants, sterols have a specific role in cell proliferation and signal transduction. There are reports on the production of sterols by microalgae, yeasts, fungi, protozoa, cyanobacteria, and other types of bacteria [104].

Li et al. [75] isolated six ergosterol derivatives through ethyl acetate extraction of the endophytic fungus *Diaporthe* sp. LG23. The fungus was obtained from the leaves of a Chinese medicinal plant, *Mahonia fortune* (Berberidaceae), collected in Shanghai in the Republic of China. The compounds 3β,5α,9α-trihydroxy-(22E,24R)-ergosta-7,22-dien-6-one **(68)**, 3β,5α,9α,14α tetrahydroxy-(22E,24R)-ergosta-7,22-dien-6-one **(69)**, (22E,24R) ergosta-7,9(11),22-triene-3β,5α,6α-triol **(70)**, chaxine C **(71)**, demethylincisterol A3 **(72)**, and volemolide **(73)**, were active against gram-negative bacteria *E. coli* DSM 682 and *P. aeruginosa* DSM 22644, exhibiting MIC values of >10 µg/mL.

### 4.20. Fusaric Acid Derivatives

Zhou and collaborators [76] isolated fusaric acid **(74)**, as well as two derivatives, fusaricates H **(75)** and fusaricates I **(76).** The compounds were found in the ethyl acetate extract of the fungus *Fusarium solani* HDN15-410, obtained from the roots of *Rhizophora apiculate Blume*, which were collected in Sanya Bailu Park of Hainan Province, China. The compounds were evaluated for their antibacterial activity, both **(75)** and **(76)** presenting MIC >200 µg/mL against strains of *P. aeruginosa* and *Vibrio parahaemolyticus*, while **(74)** showed MIC of 35.8 µg mL against *P. aeruginosa* and MIC >200 µg/mL for *V. parahaemolyticus*. The compound fusaricates represent the first cases of fusaric acid butanediol esters and are diastereoisomers.

### 4.21. Helvolic Acid Derivative

The endophytic fungus *Fusarium* sp. FL10 (GenBank under accession no. JX119038) produced two derivatives of helvolic acid, helvolic acid methyl ester **(77)**, helvolic acid **(78)**, and hydrohelvolic acid **(79).** The endophyte was isolated from the leaves of the medicinal plant *Ficus carica*, which was collected from Qinling Mountain, Shaanxi Province, China. The three compounds were active against strains of *E. coli* and *P. aeruginosa*, with MIC of 6.25 and 3.13 µg/mL, respectively [77].

### 4.22. Indene Derivative

The indene derivative methyl 2- (4-hydroxybenzyl) -1,7-dihydroxy-6- (3-methylbut-2-enyl) -1H-indene-1-carboxylate **(80)** was isolated from the endophytic fungus *Aspergillus flavipes* Y-62, obtained from stem of *Suaeda glauca* (Bunge), which was collected along the Zhoushan coast, Zhejiang province, East China. The compound was evaluated for its antibacterial activity against *Klebisiella pneumoniae* and *P. aeruginosa*, with MIC of 32 µg/mL for both strains [64].

### 4.23. Indole Alkaloids

Nine indole alkaloids—namely cristatumin A–D **(81–84)**, neoechinulin A **(85)**, isoechinulin A **(86)**, variecolorin G **(87)**, preechinulin **(88)**, and tardioxopiperazine A **(89)** were identified from the fungus *Eurotium cristatum* EN-220, isolated from the marine alga *Sargassum thunbergii* kelp. All compounds were potentially active against *E. coli*, with MIC of 64 μg/mL. The fungus was grown in a rice medium at room temperature for 30 days, and alkaloids were obtained by performing exhaustive EtOAc extractions and chromatographic purification [78].

The marine environment has attracted great interest for the research of bioactive compounds. Several molecules from marine algae, as well as from algae-associated endophytic fungi, have been increasingly reported in the literature, highlighting their unique structural characteristics. The ecosystem in which these microorganisms are inserted, often under extreme pressure and salinity conditions, makes them capable of synthesizing specialized molecules, making these organisms an important source for bioprospection of molecules of pharmacological interest [105].

### 4.24. Indole Diterpenoids

Fungal indole diterpenoid metabolites are a large and structurally diverse group. Structural features shared among members of this group are a cyclic diterpene and an indole skeleton derived from geranylgeranyl diphosphate (GGPP) and indole-3-glycerol phosphate, a precursor to tryptophan [106].

Zhao and co-workers [79] isolated 11 indole diterpenoids from the endophytic fungus *Drechmeria* sp. SYPF 8335, found in the roots of *Panax notoginsen* (Burkill) F. H. Chen ex C. Y. Wu & K. M. Feng (Araliaceae), which was collected from Wenshan, Yunnan, China (23°31′6.2″ N,103°19′55.0″ E). These are drechmerin A **(90)**, drechmerin B **(91)**, drechmerin C **(92)**, drechmerin D **(93)**, drechmerin E **(94)**, drechmerin F **(95)**, drechmerin G **(96)**, terpendoles A **(97)**, terpendoles C **(98)**, terpendoles I **(99)**, and dehydroxypaxilline **(100)**, all having MIC >200 µg/mL for *P. aeruginosa* and *Klebsiella pneumoniae*. The fungus was sequenced, and your submitted and deposited at GenBank with accession numbers MF588878 (ITS), MF588890 (18S), MF588897 (28S), and MF614144 (TEF1). The results of the analysis based on the sequence of the DNA suggest a new species in the genus *Drechmeria*.

Several indole diterpenes have already been isolated. Some exhibit a diversity of biological activities, among them antiviral against the Influenza A—H1N1 virus [107], antibiotic activity [108]. Its pharmacological properties further stimulate the search for structural and biochemical elucidation of the synthesis of indole diterpenoids [100].

### 4.25. Isocoumarin and Derivatives

Two isocoumarins, diaporthin **(101)** and orthosporin **(102)**, were found in the endophytic fungus *Diaporthe terebinthifolli* LGMF907, isolated from the leaves of the medicinal plant *Shinus terebinthifolius* Raddi, which was collected in Curitiba, Paraná, Brazil (25°28′31.847″ S, 49°17′5.629″ W). The endophytic species isolated was described by Gomes et al. [109] as a new species (MycoBank MB802952). According to previous studies, the endophyte showed biological activity against *Phyllosticta citricarpa*, a fungal agent that causes black spots in citrus [110]. Based on previous studies, the authors aimed to investigate biological activity against other pathogens, observing antibacterial activity against *E. coli*. Compound **(101)** showed inhibition zones of 1.73 mm and compound **(102)** inhibition zones of 1.03 mm, with a concentration of 100 μg/disc for both compounds [80].

Chemically, isocoumarins are characterized as isomers of coumarins, with an inverted lactone ring frequently having a 3-alkyl (C1–C17) ring or a 3-phenyl in the α-pyrone ring. Isocoumarins are natural products, and are quite abundant in fungi, bacteria, lichen and, to a lesser extent, in plants. Some genera of fungi already reported as major producers of isocoumarins are *Fusarium*, *Penicillium*, *Aspergillus*, *Artemisia*, and *Cladosporium* [111].

Two isocoumarins, (-) -5-carboxylmellein **(103)** and (-) -5-methylmellein **(104)**, were isolated from the endophytic fungus *Xylaria* sp. GDG-102 (GenBank accession number: KU645984), obtained from the leaves of the *Sophora tonkinensis*, which were collected from Hechi, Guangxi Province, China. The two compounds were active against *E. coli* with MIC of 25 and 12.5 µg/mL, respectively [81]. These compounds have already been reported in the literature, being isolated from the marine endophytic fungus No. dz17 and *Xylaria* sp. [81].

Pinheiro and collaborators [82], studying the plant *Bauhinia guianensis* collected in the Embrapa Eastern Amazon -Belém, Brazil, isolated the endophytic fungus *Exserohilum rostratum* (ER1.1). Successive chromatographic processing of the methanolic extract yielded the compound monocerin **(105)**, with antibacterial activity showing MIC values of 15.62 µg/mL against strains of *E. coli* and *P. aeruginosa*, and MIC of 31.25 µg/mL against *Salmonella typhimurium*.

### 4.26. Naphthoquinones

Jiang and co-workers [55] isolated four naphthoquinones from the extract of the endophytic fungus *Fusarium proliferatum* AF-04 (GenBank accession no. MF426031), grown in a solid rice medium. The endophyte was isolated from the Chinese green onion. The compounds were tested for their antibacterial activity and were active against an *E. coli* strain; compounds 5-O methylsolaniol **(106)**, 5-O-methyljavanicin, and **(107)** e anhydrojavanicin **(109)** showed MIC of 25 µg/mL, while methyl ether fusarubin **(108)** presented an MIC of 50 µg/mL.

### 4.27. Phenalenone Derivative

Among the great variability of polyketides produced as fungal metabolites, phenalenones are a class of hexa- or heptaketides composed of a tricyclic system of the type perinaftenone [112]. The great structural variation in these compounds has attracted significant interest, with their bioactivities, among others, being antimicrobial, anticancer, and cytotoxic [113].

The endophytic fungus *Aspergillus* sp. A-WG-1 obtained from the tubers *Pinellia ternate*, which were collected from the suburb of Nanjing, Jiangsu Province, China, was grown in a rice medium and extracted with ethyl acetate, resulting in the identification of eleven phenalenone derivatives, aspergillussanone C-L **(110–119)** and one analog **(120)**. The compounds were evaluated for their antimicrobial bioactivity, and they exhibited the following MIC values against strains of *E. coli* and *P. aeruginosa*, respectively: compound **(110)** exhibited MIC of >50 µg/mL for both strains, **(111)** >50 and 38.48 µg/mL, **(112)** 7.83 and >50 µg/mL, **(113**) 3.93 and 26.56 µg/mL, **(114)** >50 and 24.46 µg/mL, **(115)** 5.87 and 8.59 µg/mL, **(116)** >50 and 12.0 µg/mL, **(117)** 5.34 and 28.50 µg/mL, **(118)** >50 and 6.55 µg/mL, **(119)** >50 and 1, 87 µg/mL, and compound **(120)** 1.88 and 19.07 µg/mL [83].

### 4.28. Phenolic Compounds

The 4-(2,4,7-trioxa-bicyclo [4.1.0] heptan-3-yl) phenol **(121)** isolated from the endophytic fungus *Pestalotiopsis mangiferae* (GenBank accession number HM802302), obtained from the leaves of the Mangifera indica Linn., was active against *E. coli*, *K. pneumoniae*, and *P. aeruginosa*, with MIC of 1.25 µg/mL, 0.039 µg/mL, and 5.0 µg/mL, respectively [84].

Compounds derived from phenolic sulfates are not commonly isolated from microorganisms. Zhou and collaborators [85] reported three phenolic sulfate derivatives from the endophytic fungus *Stemphylium* sp. 33231, named stemphol A **(122)**, stemphol B **(123)**, and stemphol **(124)**. The three compounds were obtained from fermentation in potato broth and glucose of the endophyte isolated from fresh leaves of *Bruguiera sexangula* var. rhynchopetala, which were collected in the China Sea. The antimicrobial activity was determined against pathogenic bacteria, with compound **(122)** showing an MIC of 5.0 µg/mL, while the other two compounds **(123)** and **(124)** presented an MIC of 0.6 µg/mL against the *E. coli* strain.

### 4.29. Phomosine Derivatides

Sousa and collaborators [86] isolated two phomosine derivative from the leaves of fungus endophytic *Diaporthe* sp. F2934, isolated from the plant *Siparuna gesnerioides* (Kunth) A. DC (Siparunaceae). This species of the plant is used to treat gastrointestinal disorders, fever, and rheumatism. Crude extract was obtained from the mycelium using ethyl acetate and sonication, and was fractioned using classic chromatography and HPLC. The isolated compound Phomosine A **(125)** was active against *Enterococcus cloacae*, showing 11 mm of inhibition zone diameters, while phomosine C **(126)** presented 8 mm of inhibition zone.

### 4.30. Phthalate

Silva et al. [54] isolated a Di (2-ethylhexyl) phthalate (DEHP) **(127)** from the endophytic fungus *Diaporthe phaseolorum* (8S) found in the Amazonian plant *Paullinia cupana* var. *sorbilis* (Mart.), which was collected in the Embrapa, located in the cities of Manaus (2°53′29.14″ S and 59°58′39.90″ W, 99 m high) and Maue’s (3°22′ 54″ S and 57°42′55″ W, 18 m high), State of Amazonas, Brazil. Compound **(127)** showed antibacterial activity against multi-resistant and sensitive *E. coli* strains, as well as multi-resistant and sensitive *P. aeruginosa* strains, with MIC values of >30 µg/mL for *E. coli* and 0.23 µg/mL for *P. aeruginosa*. This was the first report on the isolation of DEHP from the genus *Diaporthe*.

The (DEHP) **(127**) is a plasticizer widely used as a softening agent in the production of plastic, e.g., polyvinyl chloride (PVC), and its overuse has turned it into an environmental problem [114]. This compound is present in tubes and resins used in the laboratory for extraction of natural products, and its isolation by researchers often raises the question of whether DEHP is, in fact, a natural product, or a contaminant from laboratory equipment and packaging [115].

### 4.31. Phthalide Derivative

The endophytic fungus *Xylaria* sp. GDG102 (GenBank accession number: KU645984) was isolated from the leaves of an important medicinal plant used in China, *Sophora tonkinensis*, which was collected from Hechi, Guangxi Province, China. The endophyte was grown in a PDB medium for 50 days, and it generated the phthalide derivative named xylarphthalide A **(128)**, which was active against *E. coli* with MIC of 12.5 µg/mL [81].

### 4.32. Polyketide and Derivative

A polyketide derived from benzofuranone was isolated from the endophytic fungus *Epicoccum nigrum* SCNU-F0002 (Gen Bank with accession no. MN096740), found in the plant *Acanthus ilicifolius* L., which was collected from the Qi’ao island Mangrove Nature Reserve in Guangdong Province, China. The compound 1- (4-hydroxy-2-methoxybenzofuran-5-yl) butan-1-one **(129)** was active against *E. coli* and *P. aeruginosa*, with MIC of 50 and >100 µg/mL, respectively [87].

Wang and co-workers [88] investigated specialized metabolites from the endophytic fungus *Colletotrichum* sp. BS4 (EMBL-Bank accession number LN552210), obtained from the leaves of *Buxus sinica* (Buxaceae), a plant traditionally known in Chinese medicine for its antibacterial activity, which was collected from Guangzhou, Guangdong Province, People’s Republic of China. The authors isolated four polyketides from the solid rice medium, which were active against gram-negative bacteria *E. coli* and *P. aeruginosa*. The compound colletotrichone A **(130)** showed MIC of 1.0 µg/mL and >10 µg/mL against *E. coli* and *P. aeruginosa*, respectively, the compound colletotrichone B **(131)** showed MIC of >10 µg/mL for both for strains; the compound colletotrichone C **(132)** exhibited MIC of 5.0 µg/mL and >10 µg/mL for *E. coli* and *P. aeruginosa*, respectively; and the compound chermesinone B **(133)** showed MIC of >10 µg/mL for both bacteria.

*Penicillium* species are known for their ability to produce diverse metabolites [116,117], in addition to being sources of polyketides, such as griseofulvin [118] and mevastatin [119], two polyketides with antifungal properties of extreme importance for the pharmaceutical industry. Jouda et al. [89] isolated three undescribed polyketides, called penealidins A-C **(134–136)**, from the endophytic fungus *Penicillium* sp. CAMMC64, found in the leaves of *Garcinia nobilis* (Clusiaceae), which were collected in Mount Etinde, Southwest region, Cameroon. The compounds were active against *Acinetobacter* sp. and showed MIC of >10.0 for the three compounds. The compound **(134)** presented MIC of >10 µg/mL against *E. coli* DSM1116, and MIC of 10.0 µg/mL for the compounds **(135** and **136)** against strain *E. coli.* DSM1116. For the *E. coli* DSM682, the compound **(134)** showed MIC of >10.0 µg/mL and MIC of 10.0 µg/mL for the compounds **(135** and **136)**.

### 4.33. Polysaccharides

Polysaccharides from microorganisms have attracted attention for their properties, being used the food industry as a thickener, stabilizer, and gelling agent [120], in addition to their biological activities as an antioxidant, antitumoral [121], and anti-inflammatory [122]. Importantly, they are easier to obtain when compared to polysaccharides from plants, due to the rapid growth of microorganisms, easy cultivation, low cost, and environment-friendliness [123].

Zeng and co-workers [90] isolated two polysaccharides not previously described, which were produced by the endophytic fungus *Fusarium solani* DO7, named DY1 **(137)** and DY2 **(138)**, which were evaluated for antibacterial activity. Compound **(137)** showed MIC of 20 and 25 µg/mL against strains of *E. coli* and *Salmonella*, respectively, while compound **(138)** exhibited an MIC of 15 and 20 µg/mL for *E. coli* and *Salmonella*, respectively. The endophytic was isolated of the *Dendrobium officinale* (Dendrobium officinale Kimura et Migo) and stored in the China Center for Type Culture Collection (CCTCC, No. M2017145) [90].

The polysaccharides produced by microorganisms are classified according to their location in the cell. Cytosolic polysaccharides provide carbon and energy to the cells. Polysaccharides that are present in the cell wall include those of the peptidoglycan and lipopolysaccharide type, and the group of polysaccharides that are exuded in the form of capsules or biofilms, known as exopolysaccharides (EPSs) [120].

Endophytic fungi are capable of producing a variety of structurally different polysaccharides. Orlandelli et al. [124] observed the production of polysaccharides by species of endophytic fungi present in the leaves of the medicinal plant *Piper hispidum.* The fungi are of the genus *Diaporthe*, *Marasmius*, *Phlebia*, *Phoma*, *Phyllosticta*, and *Schizophyllum*. The authors found that the endophytes were capable of producing polysaccharides, awarding special mention to the genus *Diaporthe*, which had the highest polysaccharide production in a 96 h-period. This was interesting mainly because it is economically viable.

### 4.34. Pyrones

A derivative of radicinol **(139)** was isolated from the fungus *Epicoccum nigrum* SCNU-F0002 (Gen Bank with accession no. MN096740), an endophytic found in the mangrove plant *Acanthus ilicifolius* L., which was collected in January 2018 from the Qi’ao island Mangrove Nature Reserve in Guangdong Province, China. The compound was active against strains of *E. coli* and *P. aeruginosa* with MIC >100 µg/mL [87].

Pyrones of the type solanapyrones were isolated from the endophytic *Alternaria tenuissima* SP-07 (GenBank accession no. KF919124) from the roots of *Salvia przewalskii* Maxim (Lamiaceae), which were collected randomly from Longxi County, Gansu Province of China. The genus *Alternaria* is a type of ascomycete and is the most well-known plant pathogen. From the ethyl acetate extract, it was possible to obtain solanopyrones P, Q, and R **(140–142)** and solanopyrones A, B, and C **(143–145)**, all possessing antibacterial activity against *E. coli* [63].

### 4.35. Sesquiterpenoids and Terpenes Derivatives

Two undescribed serquiterpenoids, leptosphins A **(146)** and B **(147)**, were identified in the solid-state fermentation culture of the endophytic fungus *Leptosphaeria* sp. XL026, deposited at GenBank with accession no. MK603060. These were isolated from *Panax notoginseng* leaves, which were collected in September 2015 from Shijiazhuang, Hebei province, P. R. China. These molecules showed antibacterial activity against *E. coli*, *P. aeruginosa*, and *Salmonella typhimurium* strains, with MIC ranging from 12.5 to >100 µg/mL. Compound **(146)** represents the first sulfur-containing sesquiterpene eremophyllane [67].

Jiang and co-workers [55] characterized three sesquiterpenoids and one sestertepene from the fungus *Fusarium proliferatum* AF-04 (GenBank accession no. MF426031), isolated from green Chinese onions. All five compounds showed antibacterial activity against *E. coli*. The sesquiterpenoids epicyclonerodiol oxide **(148)**, cyclonerodiol lactone **(149)**, and 3β-hydroxy-β-acorenol **(150)** exhibited MIC >50, >100 and >100 µg/mL, respectively. The serterterpene fusaproliferin **(151)** both exhibited MIC above 100 µg/mL against *E. coli*.

### 4.36. Sirenin Derivatives

Two sirenin derivatives, eupenicisirenin A **(152)** and eupenicisirenin B **(153)**, were isolated from the endophytic fungus *Eupenicillium* sp. LG41 EMBL-Bank (accession number LN626295), which was found in the roots of *Xanthium sibiricum* (Asteraceae) collected from Taian, People’s Republic of China. The compounds were evaluated against *Acinetobacter* sp. BD4 (DSM 586) and *E. coli* (DSM 1116). Compound **(152)** presented MIC of 5.0 µg/mL for both strains, while compound **(153)** exhibited MIC of 5.0 and 10.0 µg/mL for *Acinetobacter* sp. and *E. coli*, respectively [71].

### 4.37. Triterpenoid

A tetracyclic triterpenoid with an aromatic B-ring system was isolated from the endophytic fungus *Diaporthe* sp. LG23. The sequence of the identified was deposited at the EMBL-Bank (accession number LN552209). The endophytic was isolated from the leaves of the medicinal plant *Mahonia fortune* (Berberidaceae), which was collected in Shanghai, People’s Republic of China. The compound 19-norlanosta-5 (10), 6,8,24-tetraene-1α, 3β, 12β, 22S-tetraol **(154)** represents a rare fungal derivative of the tetracyclic triterpenoid class. This triterpenoid was evaluated for antibacterial activity against strains of *E. coli* and *P. aeruginosa*, and demonstrated pronounced efficacy against the microorganisms tested with MIC 5.0 and 2.0 µg/mL, respectively [75].

### 4.38. Xanthones

Shang and collaborators [61] isolated the endophyte *Nigrospora* sp. MA75 (GenBank with accession No. HQ891662) from the marine plant *Pongamia pinnata*, which was collected from the Guangxi Zhuang Autonomous Region of China. After fermentation and ethyl acetate extraction, it was possible to obtain two compounds, the xanthone 3,6,8-trihydroxy-1-methylxanthone **(155)**. The compound was active against *E. coli*, showing MIC of 32 µg/mL.

### 4.39. α-Pyrone and Derivatives and γ-Pyrones Derivatives

Cai and collaborators [91] reported the isolation of α-pyrones phomopyrone A **(156)**, acropyrone **(157)**, and ampelanol **(158)** from the endophytic fungus *Phomopsis* sp. HNY29-2B (GenBank accession No. KF387574) from a mangrove plant, Ac*anthus ilicifolius*, which was collected from South China Sea in Hainan Province, China. Compounds **(156)** and **(158)** showed MIC above 100 µg/mL, while **(157)** showed an MIC of 50 µg/mL against *P. aeruginosa.*

Three α-pyrone derivatives not previously described, called 6-(2′R-hydroxy-3′E, 5′E-diene-1′-heptyl)-4 hydroxy-3-methyl-2H-pyran-2-one **(159)**, 6-(2′S-hydroxy-5′E-ene 1′-heptyl)-4 hydroxy 3-methyl-2H-pyran-2-one **(160)**, and 6-(2′S-hydroxy-1′-heptyl)-4 -hydroxy-3-methyl 2Hpyran-2-one **(161)**, as well as trichodermic acid **(162)**, were reported in solid-state fermentation of *Penicillium ochrochloronth* MPT-163 (GenBank Accession No. MG818953) [92]. This endophyte lives in association with the roots of *Taxus media* collected in Mountain, Chongqing, China. The compounds showed MIC values between 25 and 100 µg/mL against *Enterobacter aerogenes*, *E. coli*, *P. aeruginosa*, *Salmonella enterica*, and *Salmonella typhi* strains.

Zhou and collaborators [93] isolated two α-pyrone derivatives, infectopyrone A and B **(163** and **164)**, both showing potent antimicrobial activity against *E. coli* (MIC of 2.5 µg/mL). The compounds were isolated of the extract crude of the EtOAc from the endophytic fungus *Stemphylium* sp. 33231 (GenBank, with an accession number KF479349) from the leaves of *Bruguiera sexangula* var. *rhynchopetala*, which was collected in the South China Sea.

Infectopyrone is an α-pyrones derivative originally isolated from the fungus *Alternaria infectoria.* The difference between infectopyrone and **(163)** is the lack of a methyl signal in infectopyrone and the presence of an oxygenated methylene signal in (**164)** [125].

Zhou and co-workers [76] isolated two γ-pyrone derivatives, Fusalanones A and B **(165** and **166)** from the endophytic fungus *Fusarum solani* HDN15-410 (GenBank accession number: MH383099), isolated from the root of *Rhizophora apiculata Blume* (Rhizophoraceae), which was collected from Sanya Bailu Park of Hainan Province, China. The two compounds were the first reports of γ-pyrone isolated from *F. solani*. These metabolites showed activity against strains of *P. aeruginosa* and *Vibrio parahaemolyticus*; however, it was the compound **(166)** that demonstrated the best antimicrobial activity, with a MIC value of 6.25 µg/mL for the *V. parahaemolyticus* strain and 12.5 µg/mL for *P. aeruginosa*.

Endophytic microorganisms have played a very important role over the life span of plants through the production of secondary metabolites. They contribute in different ways, whether in the adaptation of plants to environmental stresses such as changes in temperature, scarcity of nutrients, water deficit, and radiation sunlight, or against biotic stresses such as the attack of herbivores, insects, or other microorganisms [34].

**Figure 2:**
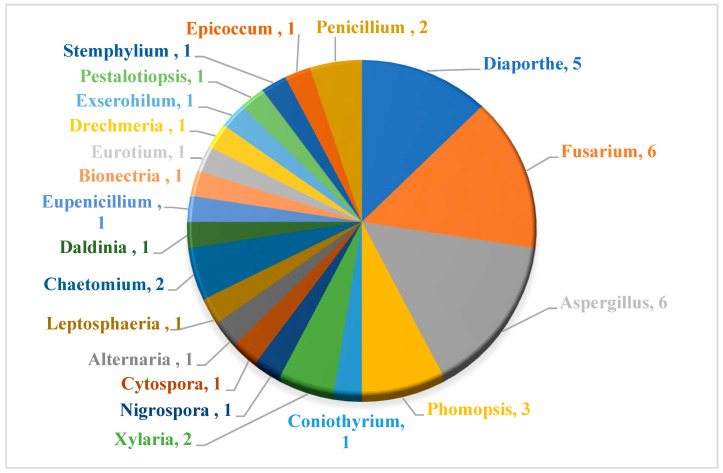
General graphic of the fungal endophytic genera surveyed in this review.

**Figure 3:**
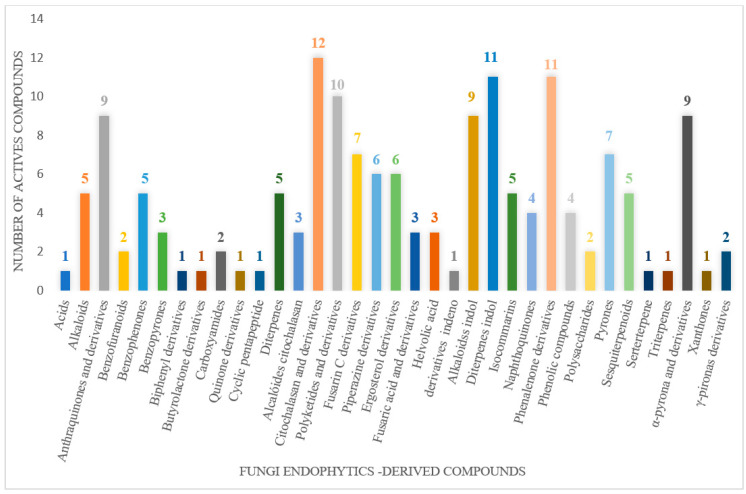
Number of compounds actives derivatives of fungal endophytic contemplated in this review.

The wide variety of molecules produced by endophytic microorganisms is impressive. In this bibliographic survey, it was possible to identify the endophytic fungal species isolated, which tissue the endophytic was isolated, and the region where the plant parts were collected, as well as the chemical classes, the molecule, and its MIC values against gram-negative bacteria. Thus, 21 genera of fungi were described as shown in Figure 2, with *Aspergillus* (6), *Fusarium* (6), and *Diaporthe* (5) being the most described in this review. Of the 126 articles surveyed, 43 chemical classes were categorized and a total of 166 active molecules were described, as shown in Figure 3.

The most varied chemical classes were observed, including (i) acids; (ii) alkaloids; (iii) anthraquinones and derivatives; (iv) benzofuranoid; (v) benzophenone and derivatives; (vi) benzopyrones; (vii) biphenyl derivative; (viii) butyrolactone derivative; (ix) carboxamidas; (x) cochlioquinone derivative; (xi) cyclic pentapeptide; (xii) cyclopiane-type diterpene; (xiii) cytochalasan alkaloids; (xiv) cytochalasin and derivatives; (xv) decalin polyketides; (xvi) depsipeptide; (xvii) fusarin C derivatives; (xviii) diketopiperazine; (xix) epipolythiodioxopiperazine; (xx) ergosterol derivatives; (xxi) fusaric acid e derivatives; (xxii) helvolic acid derivative; (xxiii) Indene derivative; (xxiv) indole alkaloids; (xxv) indole diterpenoids; (xxvi) isocoumarin and derivatives; (xxvii) naphthoquinones; (xxviii) phenalenone derivatives; (xxix) phenolic compound; (xxx) Phenolic sulfate derivatives; (xxxi) phomosine derivatives; (xxxii) phthalate; (xxxiii) phthalide derivative; (xxxiv) polyketide; (xxxv) polysaccharides; (xxxvi) Pyrones; (xxxvii) sesquiterpenoids; (xxxviii) sesterterpene; (xxxix) sirenin derivatives; (xl) triterpenoid; (xli) xanthones; (xlii) α-pyrone and derivatives; and (xliii) γ-pyrones derivatives.

**Figure 4:**
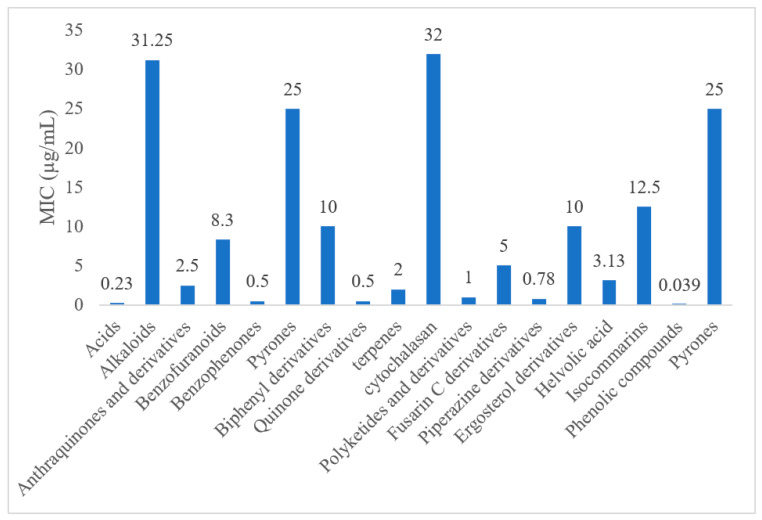
Major chemical classes with the lowest MIC values against gram-negative bacteria.

The most abundant chemical components were anthraquinones, cytochalasans, polyketides, alkaloids, terpenes, phenalenones, and pyrones. The MIC values of the isolated compounds showed encouraging values, as the vast majority presented MIC values below 50 µg/mL. The figure below (Figure 4) shows the compounds that presented the lowest MIC values against Gram-negative bacteria, with emphasis on the chemical classes of acids, anthraquinones, benzophenones, quinones, terpenes, polyketides, piperazines, helvolic acid, and compound phenolics with values of MIC lower of 5 µg/mL.

Given the MIC values found in this review, we sought to compare them with the MIC values of the main groups of antibiotics against the main groups of gram-negative bacteria. As shown in Table 2 below, we list the MIC values of the main antimicrobial agents used in clinical practice against some of the gram-negative bacterial families.

As can be seen in Table 2 above and comparing their values with the MIC values in Figure 4, it is possible to observe that some chemical classes found in this review presented MIC values well below the MICs of the antibiotics recommended by the Clinical and Laboratory Standards Institute (CLSI). We highlight the metabolites of the chemical classes benzophenones and quinones derivatives, which exhibited MICs of 0.5 µg/mL. When comparing this value with most of the MICs of the main antibiotics in Table 2, it shows how promising the metabolites of these classes of chemical compounds are. Therefore, the antibiotic potential of secondary metabolites obtained from endophytic fungi is well known.

## 5. Conclusions

The ability of endophytic microorganisms, and in particular, endophytic fungi, to produce specialized metabolites is astonishing. The great structural variability of molecules, as well as their bioactivity, is what has made endophytic fungi so promising as sources of molecules potentially active against a multitude of targets, from antimicrobials to anticancer.

The complex fungus–host plant interaction promotes the production of an extraordinary variety of molecules, often with unique characteristics. The basis for this interaction is believed to be the mutual exchange between the two organisms, in which the plant offers a nutrient-rich favorable environment for the endophyte, while the endophytic metabolites play a role in protection against external attacks, adaptive environmental resistance, and plant growth support, among others. It is important to note that there are other hypotheses for the great structural variability of the molecules found, including possible horizontal transfer of genes from the plant to the endophyte, in addition to the independent evolution of each species metabolism.

In many cases, traditional medicine guides research to isolate molecules with biotechnological potential. Several plants used in traditional folk medicine, when investigated for bioactive molecules against different diseases, yield results that corroborate their use by the population.

It is evident that not only plants can produce specialized metabolites of biological interest. As shown in this review, the vast diverse molecules isolated from endophytic microorganisms found in association with a range of plant and algae species reinforce the biotechnological potential of these endophytes. In addition to the numerous advantages of using endophytic microorganisms as sources in the search for new drugs, it is also important to emphasize that they are constantly exposed to environmental conditions that make them increasingly effective in terms of biomolecules differentiated, making them potential sources of new molecules for medicine. In addition, the low cost of work, ease of maintenance that does not require the use of sophisticated equipment, the possibility of optimizing controlled fermentation conditions and the biomass required for testing, and of favoring reproducibility for commercial scale. These conditions make such molecules an advantageous source in the search for new antibacterial drugs, reducing morbidity and mortality associated with nosocomial infections.

The increasing cases of microbial resistance, especially in gram-negative bacteria, have been drawing attention in recent years due to their impact on public health, considering the number of deaths and complications associated with microbial infections. This scenario exacerbates the need for new molecules and drug development in order to circumvent the mechanisms responsible for bacterial resistance. It is necessary to use new techniques and solutions that involve the bioprospecting of natural products; tools such as bioinformatics, chemical analysis, and biological synthesis are being increasingly sought as a solution. The genomic era has contributed greatly to the search for bioactive molecules, this is possible, through the sequencing of these microorganisms, to efficiently identify which clusters of genes are responsible for biosynthesizing certain groups of molecules. It is important to rethink the way a single molecule is effective in combating microbial resistance, for example, and to expand the idea of combining molecules that act on bacterial virulence factors.

In conclusion, there are good reasons to believe and emphasize the importance of research on the chemical diversity of natural products from endophytic microorganisms, an expanding and promising field in the discovery of new classes of antibiotics. Microbial metabolites, with their penetrating power and affinity to bacterial targets, represent a new paragon for drug development.

## Figures and Tables

**Table 1:** Molecules isolated from endophytic fungi with antibacterial activity against gram-negative species. (To be continued).

Endophyte Species (Source Plant/Tissue)	Chemical Class	Compound Name (All Name Were Kept as They Appear in the Original Article (*m*/*z*)	Structural Formula	Target Bacterium	Minimum Inhibitory Concentration (MIC) *	Reference
*Diaporthe phaseolorum* (8S) (seed)	Acids	**(1)** 3-hydroxypropionic acid (3-HPA)—C_3_H_6_O_3_/91.03897 [M + H]^+^	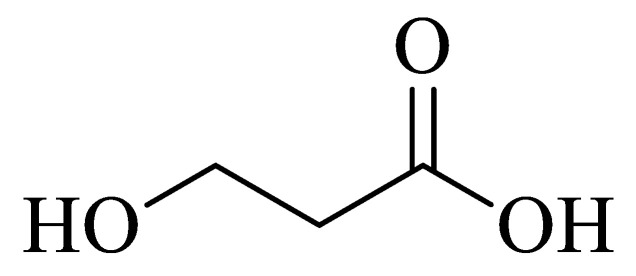	*Escherichia coli* Multiresistant strain *E. coli* Susceptible strain *Pseudomonas aeruginosa* Multiresistant strain *P. aeruginosa* Susceptible strain	>30 µg/mL >30 µg/mL 0.23 µg/mL 0.23 µg/mL	[54]
*Fusarium proliferatum* AF-04 (onion)	Alkaloids	**(2)** Indol-3-acetic acid-C_10_H_9_NO_2_/176.070605 [M + H]^+^	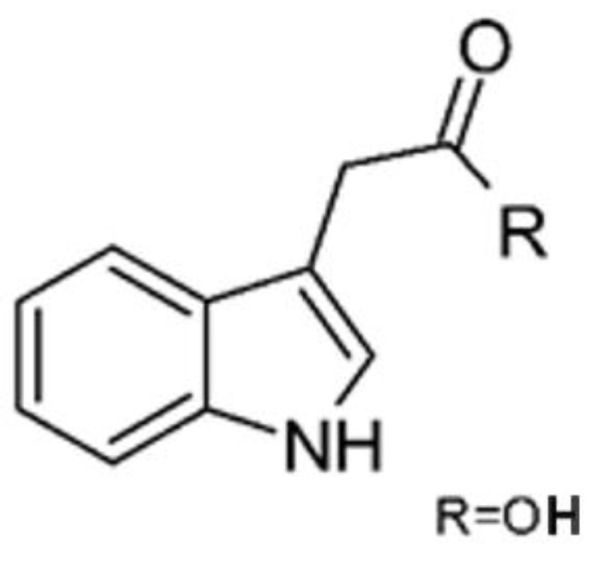	*E. coli*	>100 μg/mL	[55]
*F. proliferatum* AF-04 (onion)	Alkaloids	**(3)** Methyl indolyl-3- Acetate—C_11_H_11_NO_2_/190.086255 [M + H]^+^	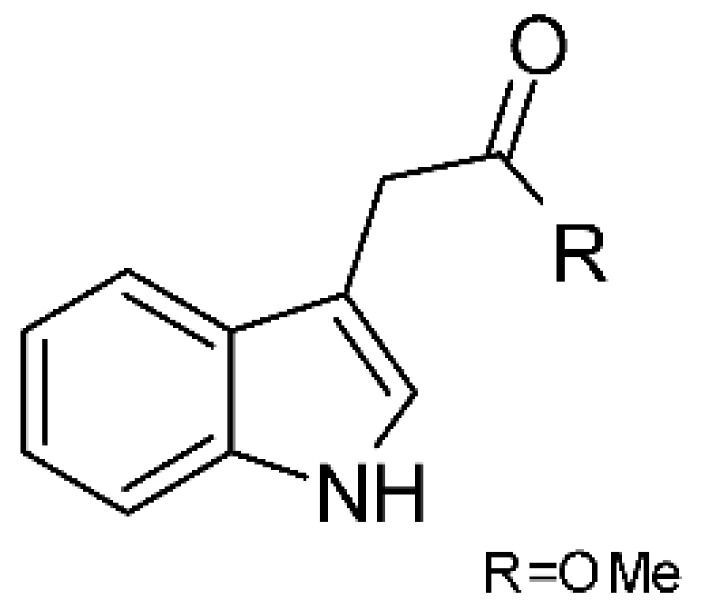	*E. coli*	>100 μg/mL	[55]
*F. proliferatum* AF-04 (onion)	Alkaloids	**(4)** Bassiatin—C_15_H_19_NO_3_/262.14377 [M + H]^+^	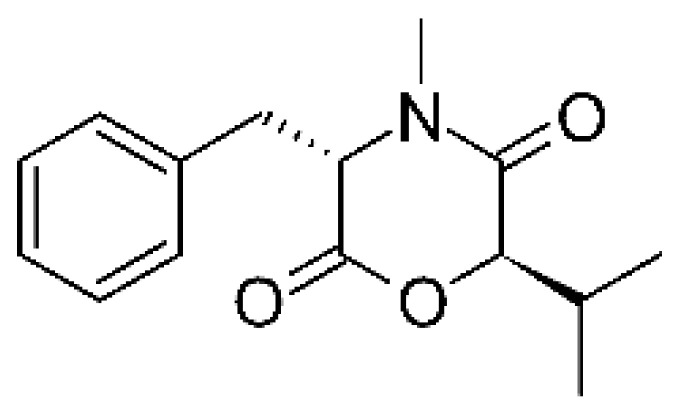	*E. coli*	>100 μg/mL	[55]
*Aspergillus* sp. EJC08	Alkaloids	**(5)** Pseurotin A—C_23_H_26_O_8_/432 [M + H]^+^	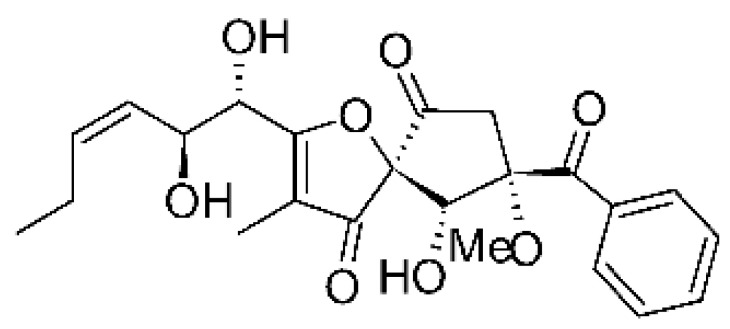	*E. coli* ATCC 25922 *P. aeruginosa* ATCC 27853	31.25 μg/mL 31.25 μg/mL	[56]
*Aspergillus* sp. EJC08	Alkaloids	**(6)** Fumigaclavine C—C_23_H_30_N_2_O_2_/367 [M + H]^+^	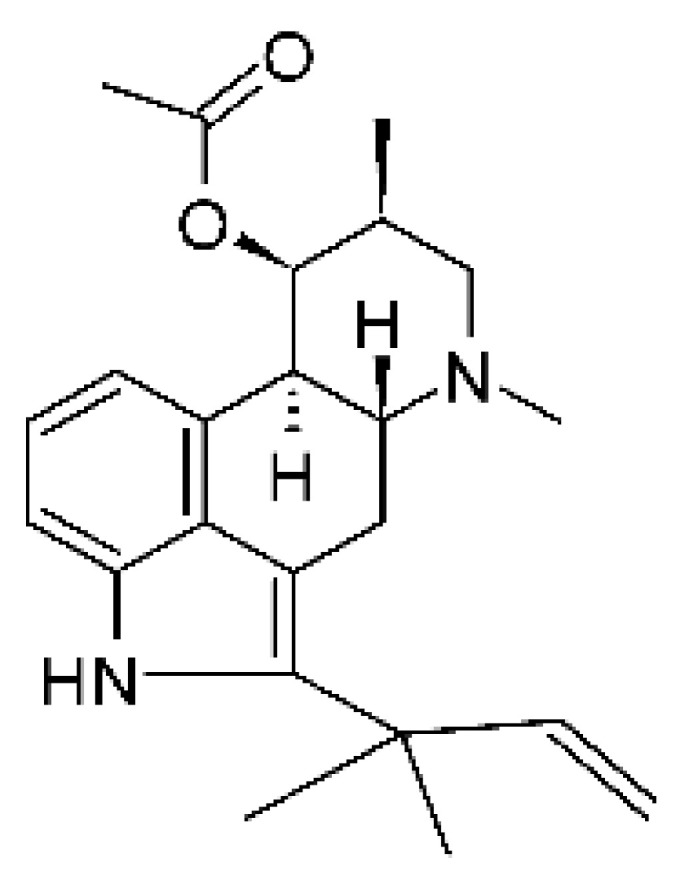	*E. coli* ATCC 25922 *P. aeruginosa* ATCC 27853	62.50 μg/mL 31.25 μg/mL	[56]
*Phomopsislongicolla* HL-2232 (mangrove)	Anthraquinones	**(7)** Altersolanol B—C_16_H_16_O_6_/305.101965 [M + H]^+^	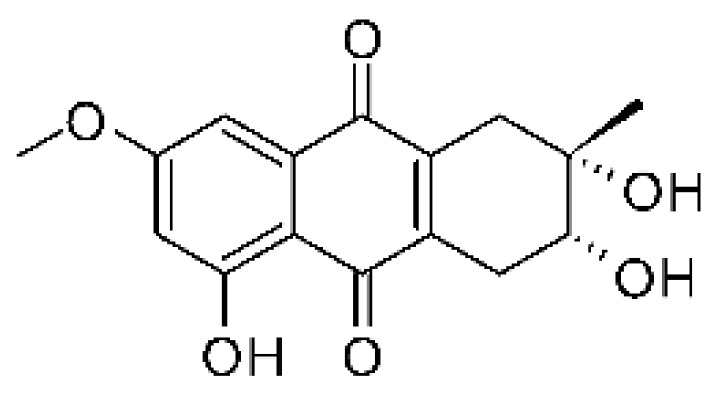	*Vibrio parahaemolyticus* *Vibrio anguillarum*	2.5 μg/mL 5 μg/mL	[57]
*Coniothyrium* sp. (internal strain No. zw86)	Anthraquinones	**(8)** 1,7-dihydroxy-3-methyl-9,10-anthraquinone—254 [M]^+^	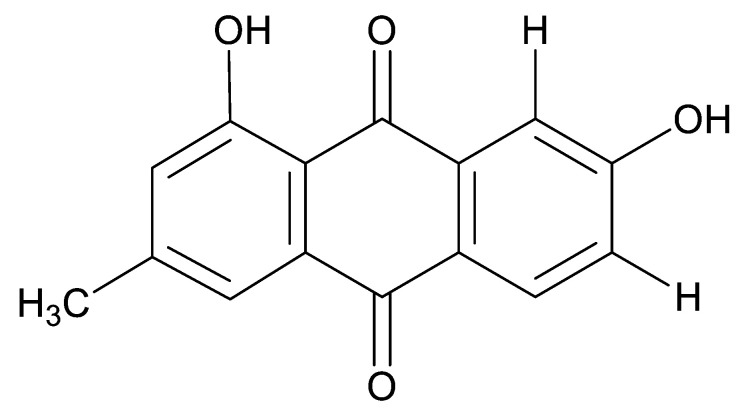	*E. coli*	11 mm	[58]
*Coniothyrium* sp. (internal strain No. zw86)	Anthraquinones	**(9)** Phomarin—C_15_H_10_O_4_/254 [M]^+^	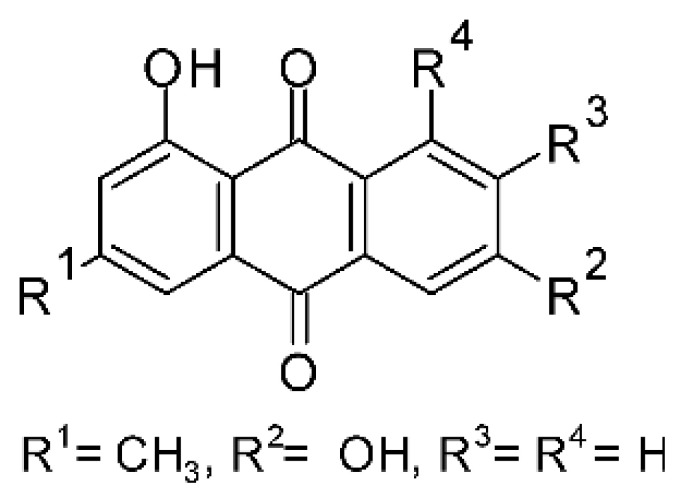	*E. coli*	11 mm	[58]
*Coniothyrium* sp. (internal strain No. zw86)	Anthraquinones	**(10)** 1-Hydroxy-3-hydroxymethyl-9,10-anthraquinone—C_15_H_10_O_4_/254 [M]^+^	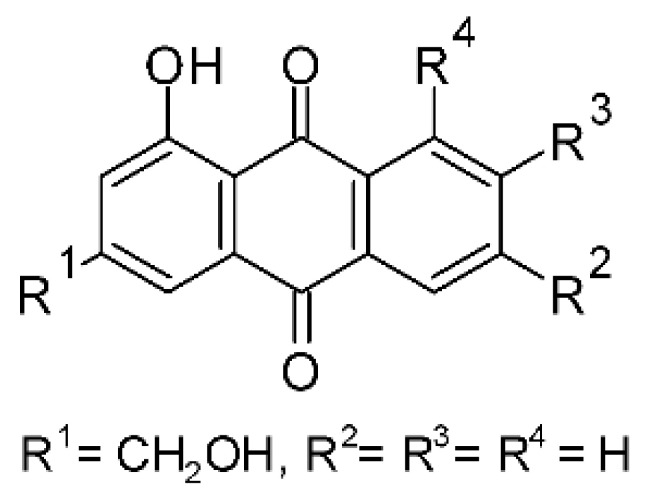	*E. coli*	15 mm	[58]
*Xylaria* sp. JK50	Anthraquinones derivatives	**(11)** Nalgiovensin—C_18_H_16_O_6_/329.101965 [M + H]^+^	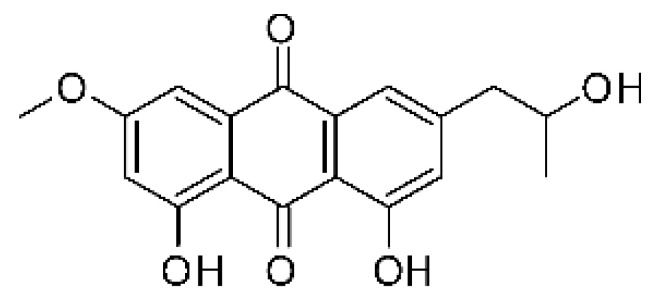	*E. coli*	50 μg/mL	[59]
*Coniothyrium* sp. (internal strain No. zw86)	Anthraquinones derivatives	**(12)** Coniothyrinones A—C_15_H_16_O_5_/276.0998 [M]^+^	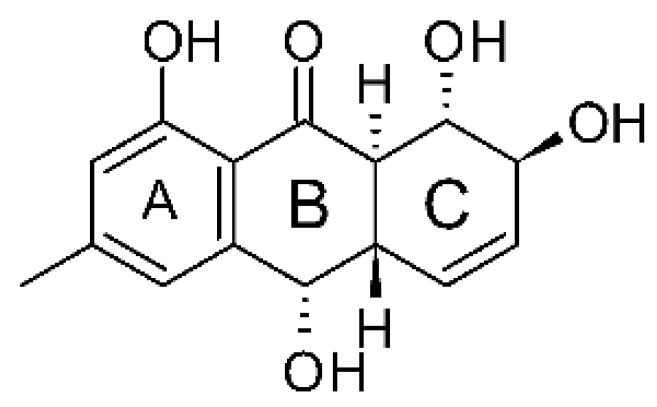	*E. coli*	7.5 mm	[58]
*Coniothyrium* sp. (internal strain No. zw86)	Anthraquinones derivatives	**(13)** Coniothyrinones B C_15_H_18_O_4_/261.1128 [M-H]^−^	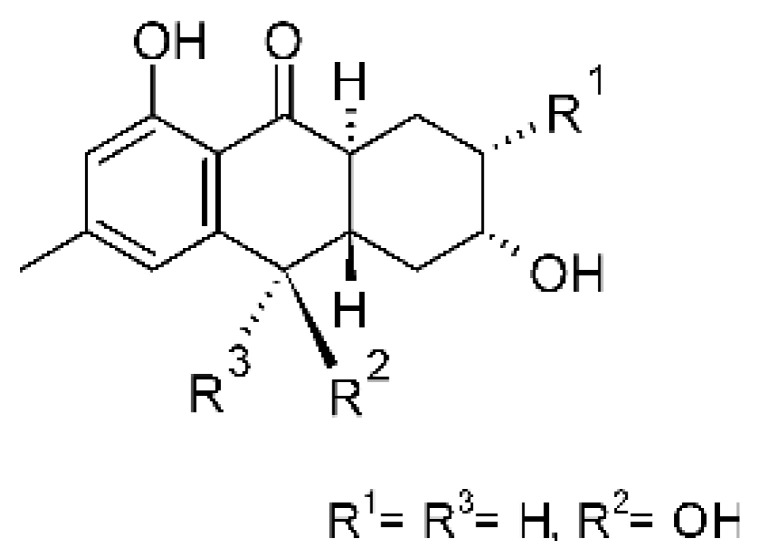	*E. coli*	6 mm	[58]
*Coniothyrium* sp. (internal strain No. zw86)	Anthraquinones derivatives	**(14)** Coniothyrinones C—C_15_H_18_O_5_/277.1078 [M-H]^−^	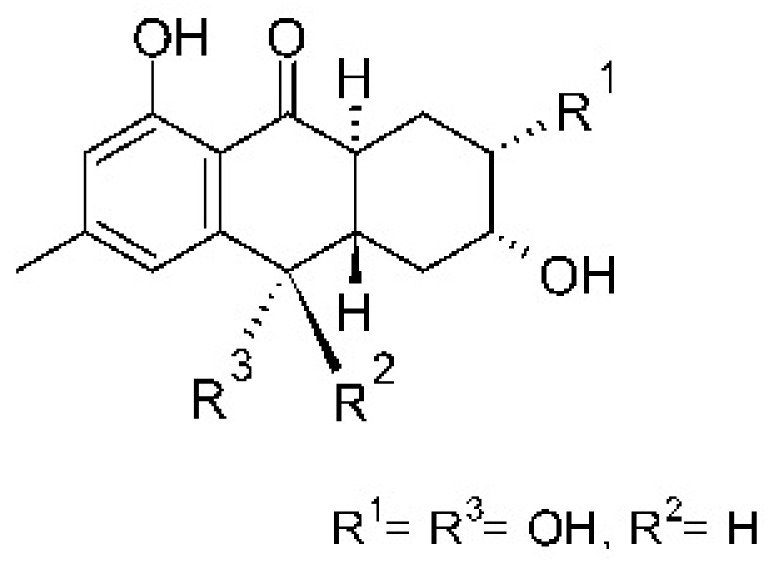	*E. coli*	7.5 mm	[58]
*Coniothyrium* sp. (internal strain No. zw86)	Anthraquinones derivatives	**(15)** Coniothyrinones D—C_15_H_18_O_5_/278.1155 [M]^+^	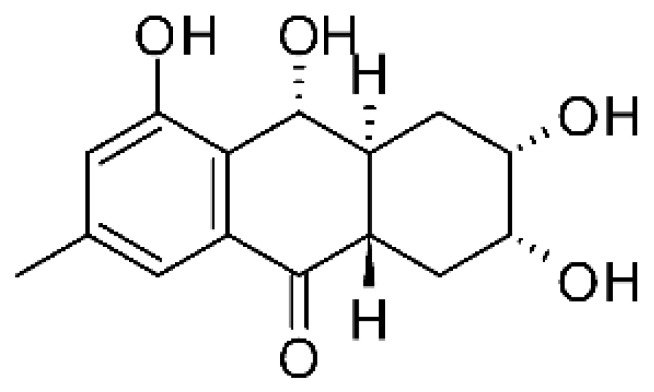	*E. coli*	6 mm	[58]
*Aspergillus* sp. ZJ-68 (leaves)	Benzofuranoid	**(16)** 2-(hydroxymethyl)-3-propylphenol—C_10_H_13_O_2_/175 [M-H]^−^	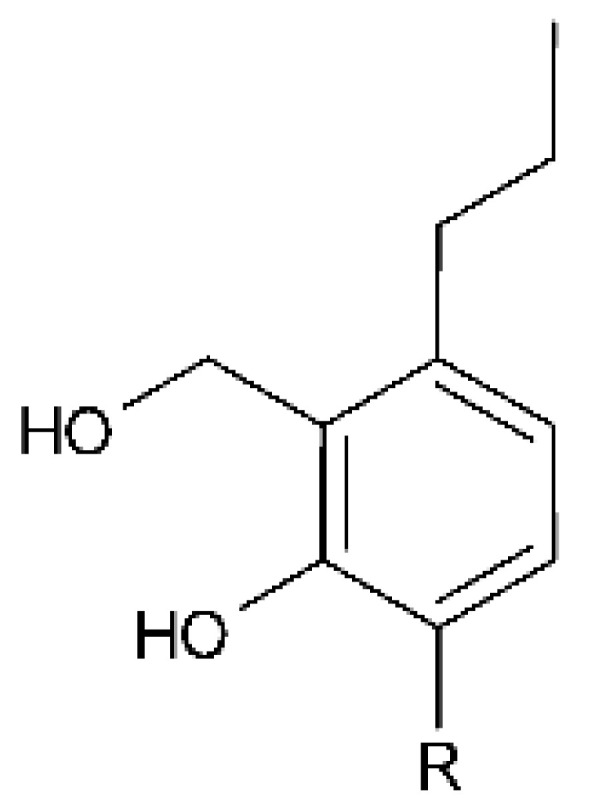	*E.coli* *P. aeruginosa*	8.3 µg/mL >100 μg/mL	[60]
*Aspergillus* sp. ZJ-68 (leaves)	Benzofuranoid	**(17)** (-)-brassicadiol—C_15_H_22_O_3_/246 [M-H]^−^	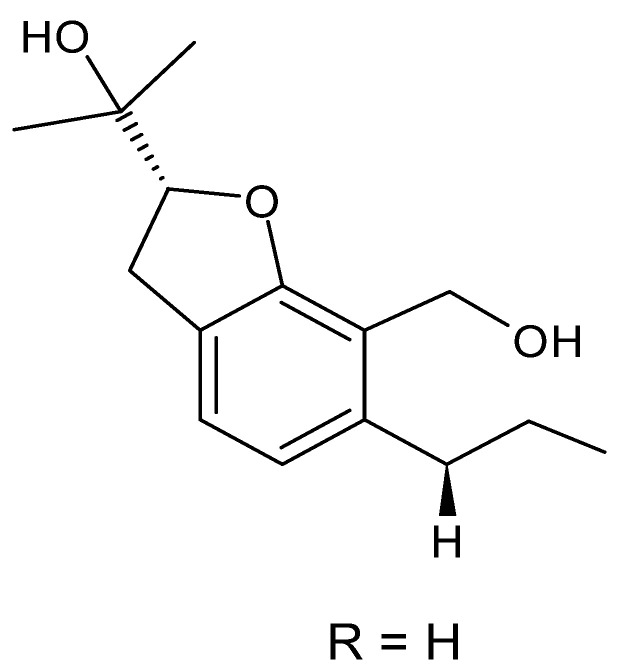	*E. coli* *P. aeruginosa*	12.5 µg/mL >100 μg/mL	[60]
*Nigrospora* sp. MA75 (stem)	Benzophenone	**(18)** Griseophenone C—C_16_H_16_O_6_/305.101965 [M + H]^+^	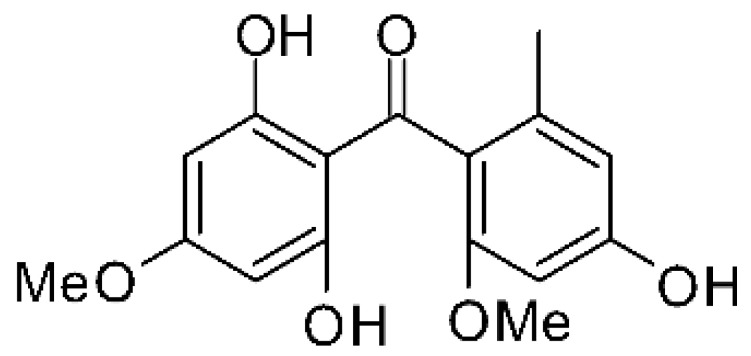	*E. coli* *P. aeruginosa*	2 μg/mL 0.5μg/mL	[61]
*Cytospora rhizophorae* A761	Benzophenone derivatives—meroterpenoide	**(19)** Cytosporins A—C_20_H_22_O_7_/373.1279 [M-H]^−^	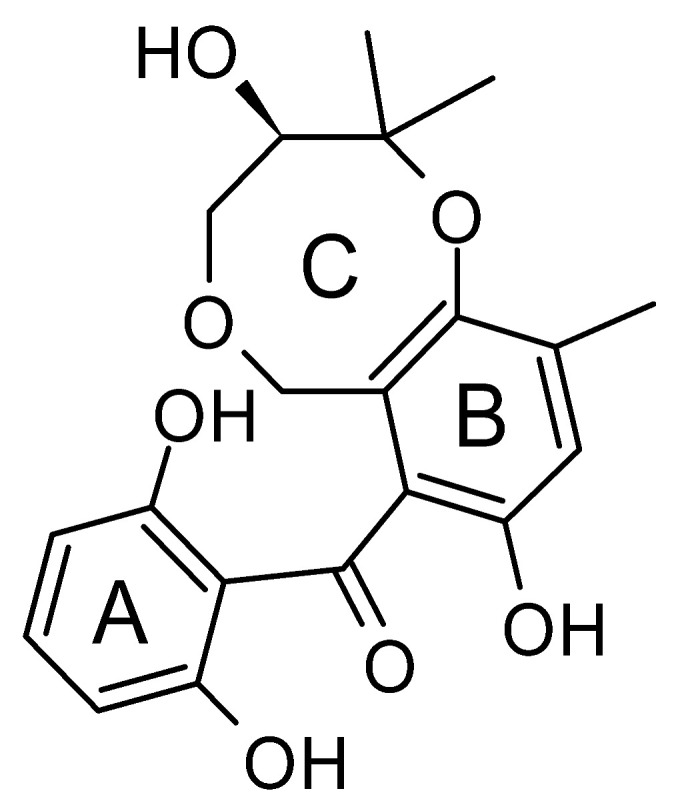	*E. coli*	250 µg/mL	[62]
*C. rhizophorae* A761	Benzophenone derivatives—meroterpenoide	**(20)** Cytosporin B—C_20_H_22_O_8_/ 387.1068 [M-H]^−^	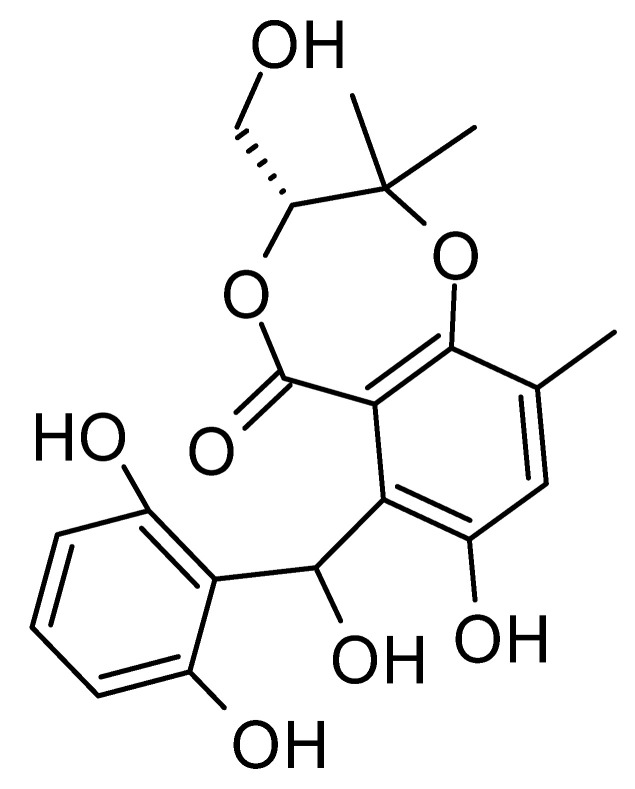	*E. coli*	250 µg/mL	[62]
*C. rhizophorae* A761	Benzophenone derivatives—meroterpenoide	**(21)** Cytosporin C—C_20_H_24_O_7_/ 377.1589 [M + H]^+^	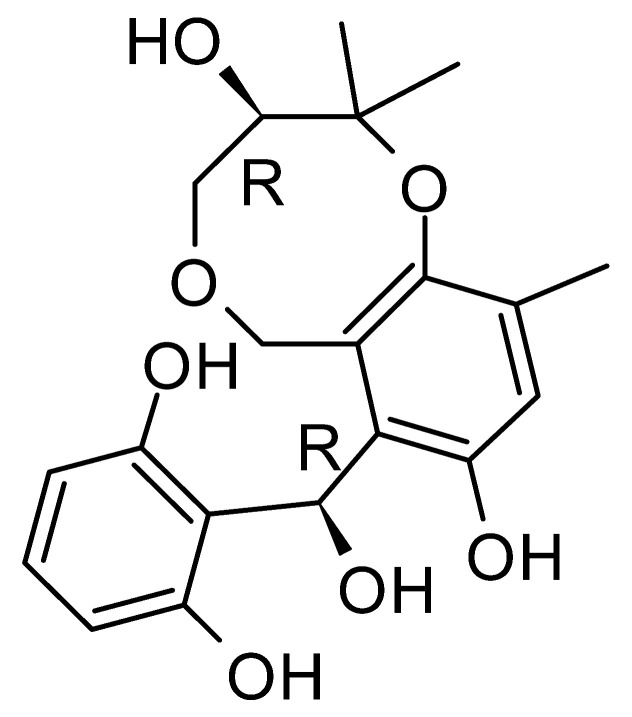	*E. coli*	250 µg/mL	[62]
*C. rhizophorae* A761	Benzophenone derivatives—meroterpenoide	**(22)** Cytosporin D—C_20_H_24_O_7_/ 377.1600 [M + H]^+^	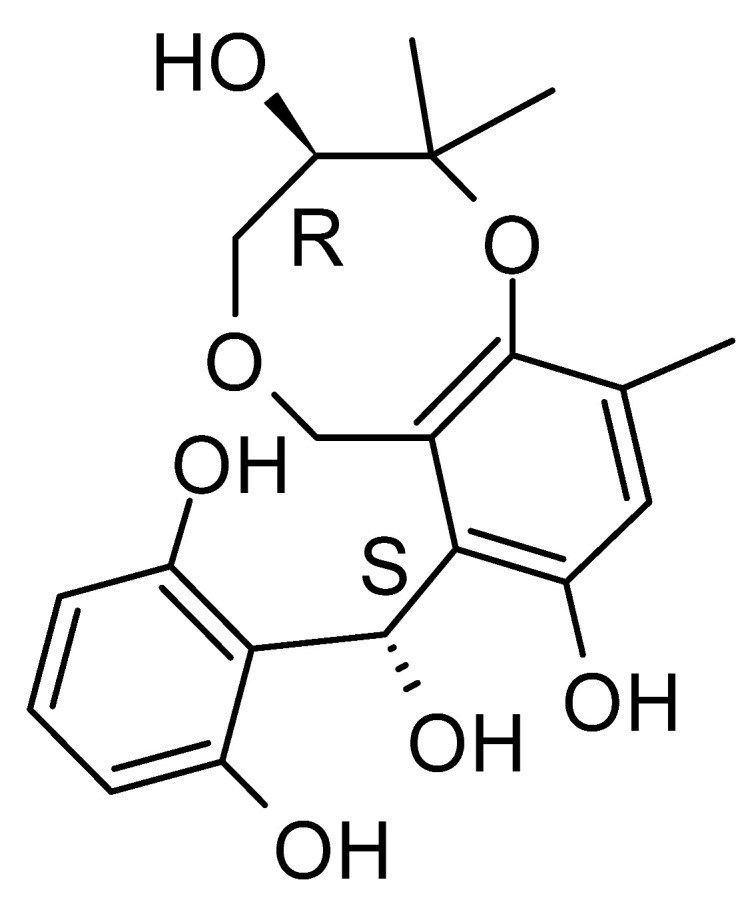	*E. coli*	250 µg/mL	[62]
*Alternaria tenuissima* SP-07	Benzopyrones	**(23)** Alternariol—C_14_H_10_O_5_/259.0601 [M + H]^+^	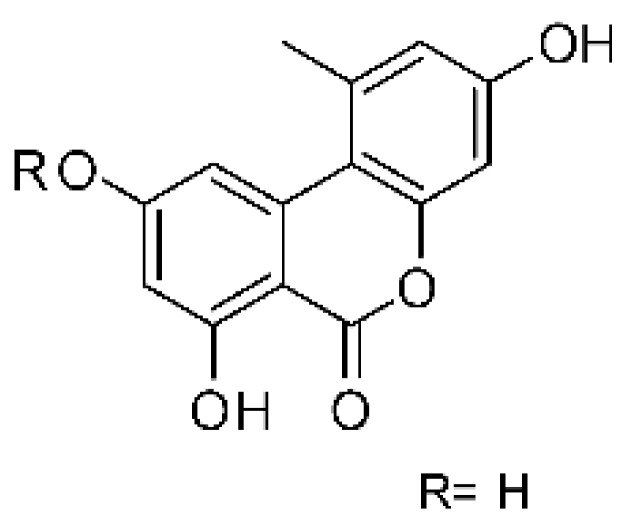	*E. coli*	100 μg/mL	[63]
*A. tenuissima* SP-07	Benzopyrones	**(24)** Alternariol methyl ether—C_15_H_12_O_5_/273.07575 [M + H]^+^	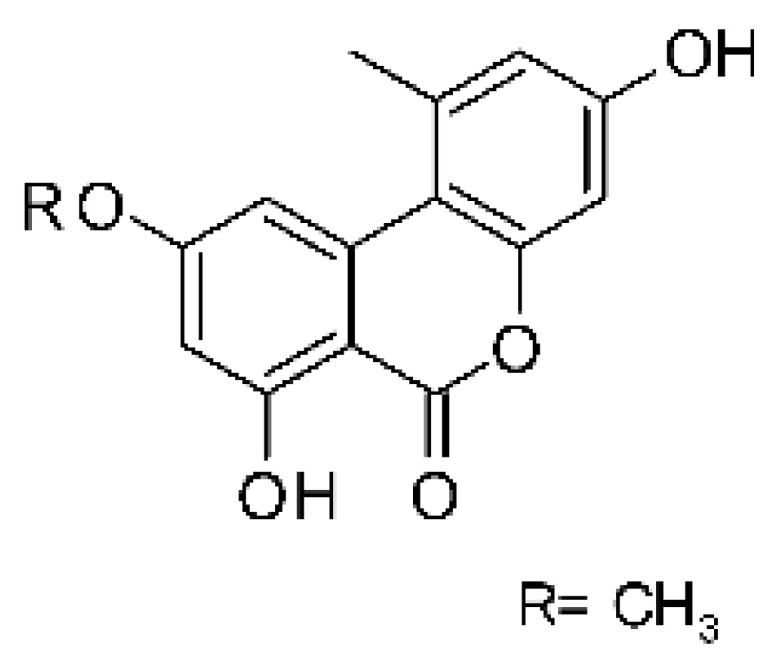	*E. coli*	>100 μg/mL	[63]
*A. tenuissima* SP-07	Benzopyrones	**(25)** Altenuene—C_15_H_16_O_6_/293.101965 [M + H]^+^	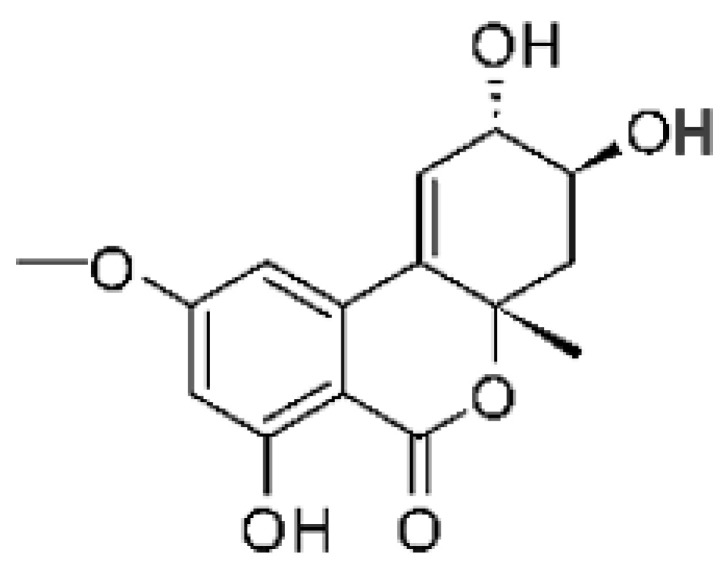	*E. coli*	100 μg/mL	[63]
*Phomopsis longicolla* HL-2232 (mangrove)	Biphenyl derivative	**(26)** 5,5′-dimethoxybiphenyl-2,2′-diol—C_14_H_14_O_4_/245.0808 [M−H]^−^	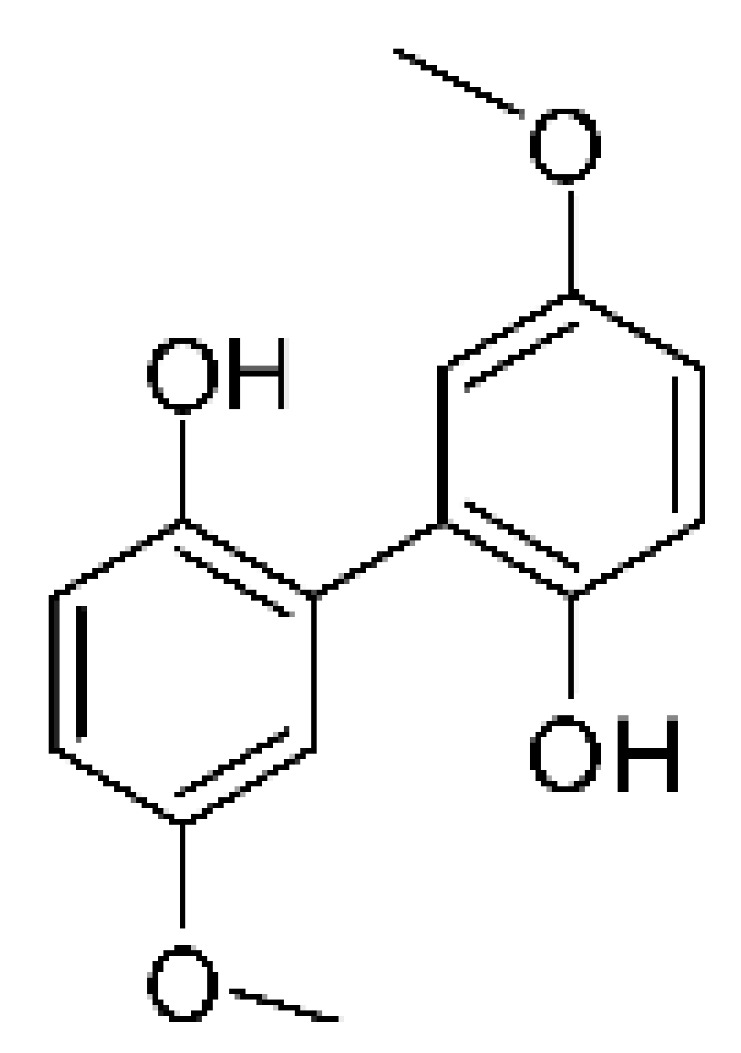	*Vibrio parahaemolyticus*	10 μg/mL	[57]
*Aspergillus flavipes* Y-62 (stems)	Butyrolactone derivative	**(27)** 2-O-methylbutyrolactone 1—C_25_H_28_O_7_/441.19078 [M + H]^+^	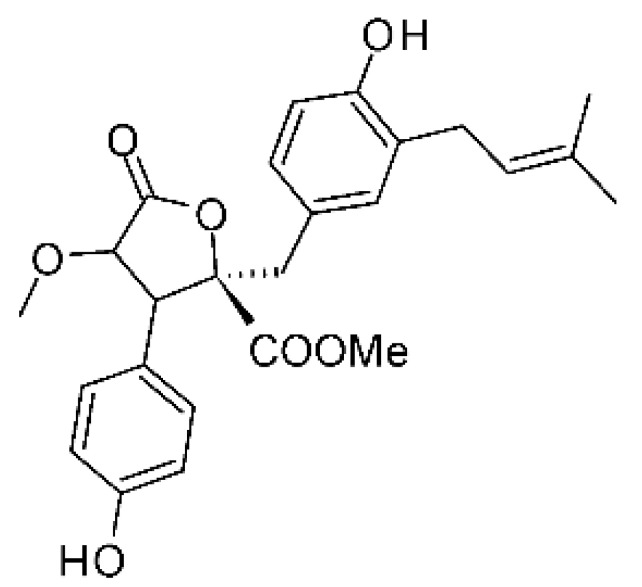	*Escherichia coli* CMCC(B) 44102	32 μg/mL	[64]
*Diaporthe vochysiae* LGMF1583 (leaves)	Carboxamidas	**(28)** Vochysiamide A—C_8_H_13_NO_4_/186 [M-H]-	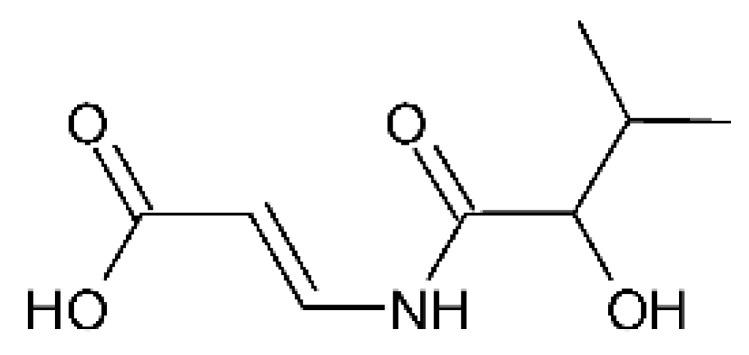	*Klebsiella pneumoniae carbapenemase-producing (KPC)*	1.0 mg/mL	[65]
*D. vochysiae* LGMF1583 (leaves)	Carboxamidas	**(29)** Vochysiamide B—C_15_H_21_O_3_/226 [M-H]^−^	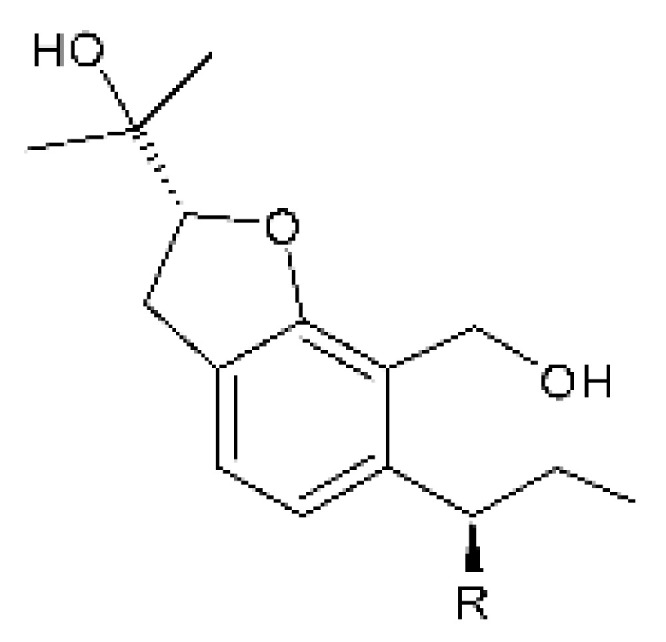	*Klebsiella pneumoniae carbapenemase-producing (KPC)*	0.08 mg/mL	[65]
*Nigrospora* sp. MA75 (stem)	Cochlioquinone derivative	**(30)** 2,3-didehydro-19ahydroxy-14-epicochlioquinone B—C_28_H_40_O_7_/509.2512 [M+Na]^+^	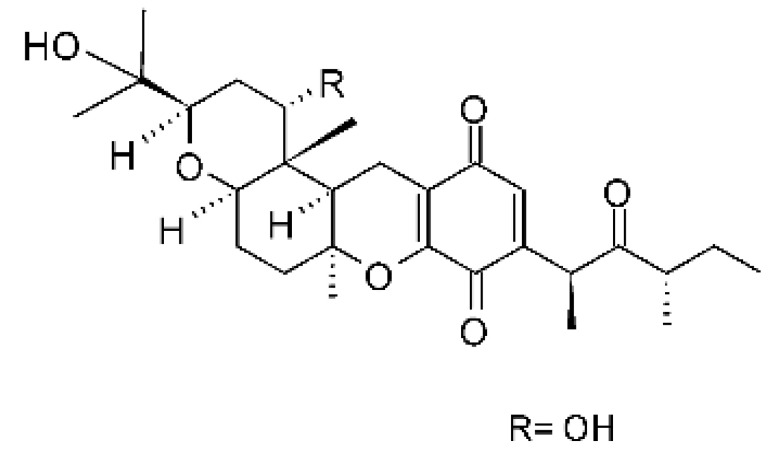	*E. coli* *P. aeruginosa* *P. fluorescens*	4 μg/mL 4 µg/mL 0.5 µg/mL	[61]
*Aspergillus tamarii* FR02 (roots)	Cyclic pentapeptide	**(31)** Malformin E—C_23_H_39_N_5_O_5_S_2_/552.2291 [M+Na]^+^	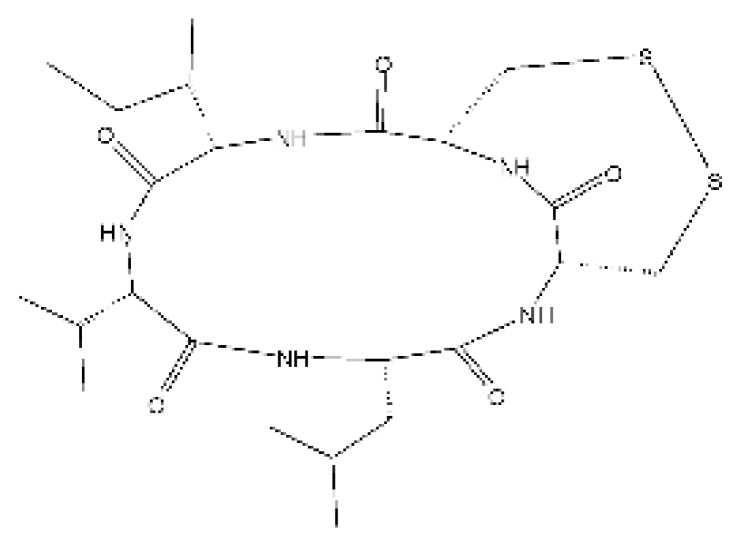	*Candida albicans* *E. coli* *P. aeruginosa*	7.24 µM 0.91 µM 1.82 µM	[66]
*Leptosphaeria* sp. XL026 (leaves)	Cyclopiane-type diterpene	**(32)** Leptosphin C—C_20_H_28_O_2_/299.2018 [M−H]^−^	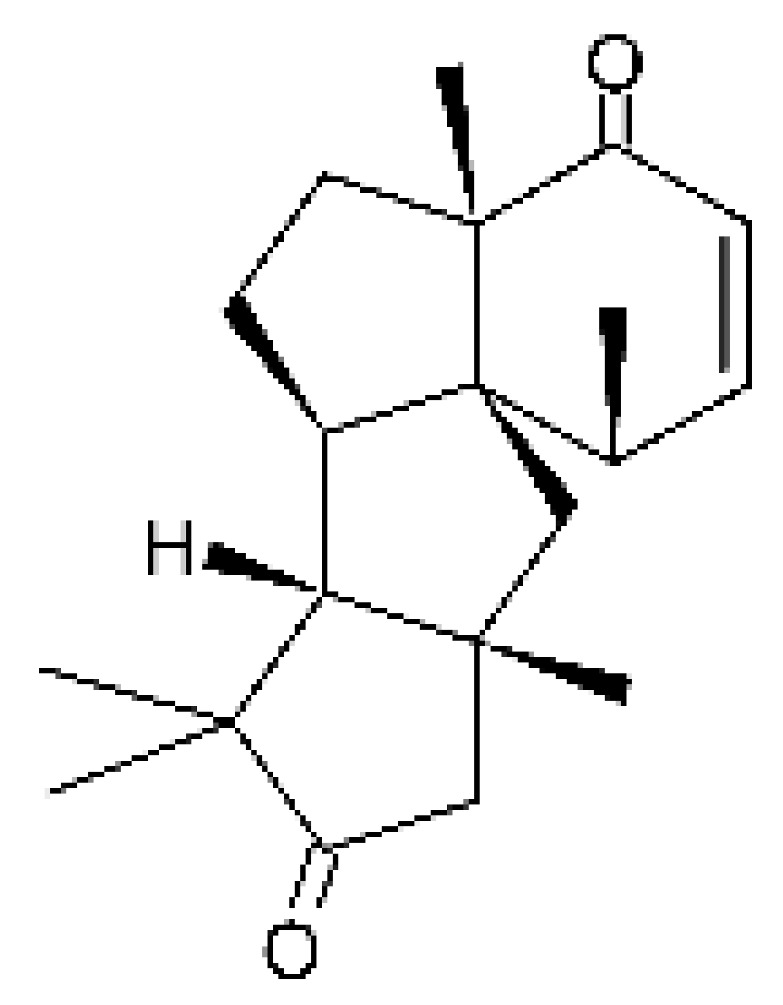	*E. coli* *P. aeruginosa* *Salmonella typhimurium*	>100 µg/mL >100 µg/mL 100 µg/mL	[67]
*Leptosphaeria* sp. XL026 (leaves)	Cyclopiane-type diterpene	**(33)** Conidiogenone F—C_20_H_30_O_2_/303.231857 [M + H]^+^	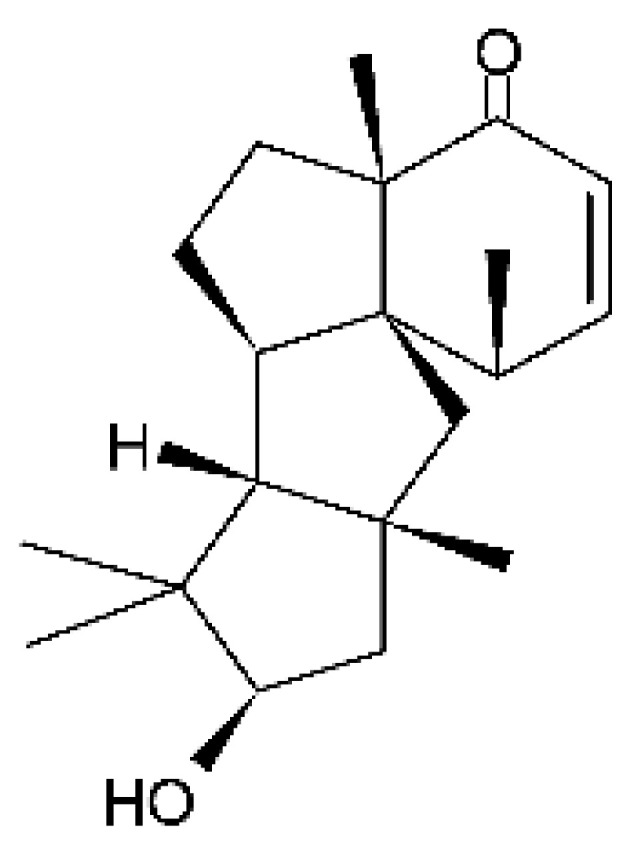	*E. coli* *P. aeruginosa* *Salmonella typhimurium*	50 µg/mL 50 µg/mL >100 µg/mL	[67]
*Leptosphaeria* sp. XL026 (leaves)	Cyclopiane-type diterpene	**(34)** Conidiogenone C—C_24_H_22_N_4_O_3_S_2_/479.120607 [M + H]^+^	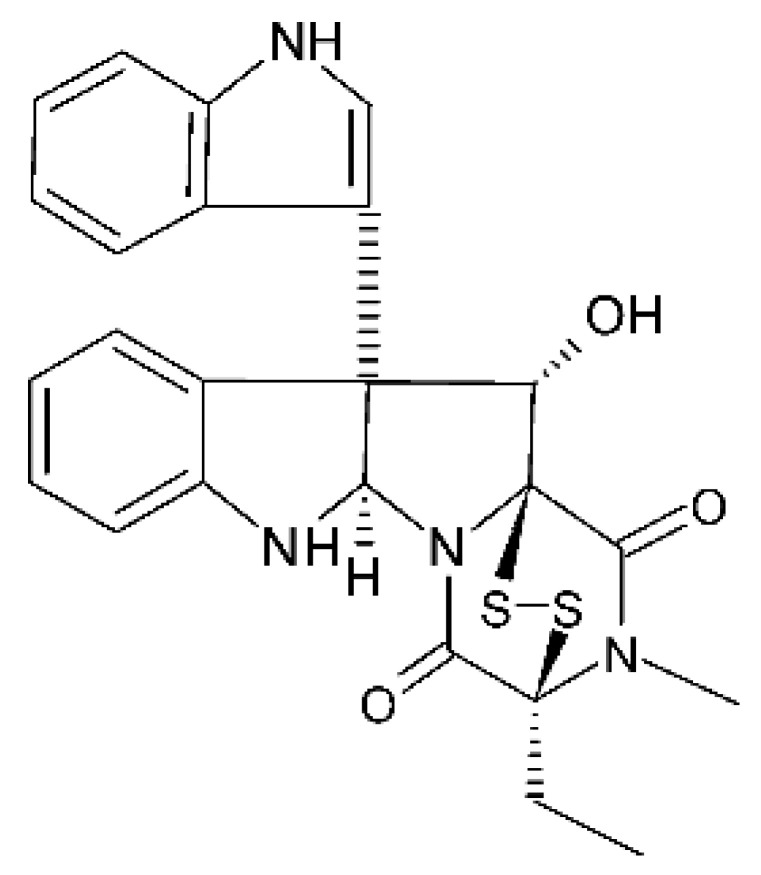	*E. coli* *P. aeruginosa* *Salmonella typhimurium*	50 µg/mL 25 µg/mL >100 µg/mL	[67]
*Leptosphaeria* sp. XL026 (leaves)	Cyclopiane-type diterpene	**(35)** Conidiogenone D—C_21_H_34_O_2_/303.231857 [M + H]^+^	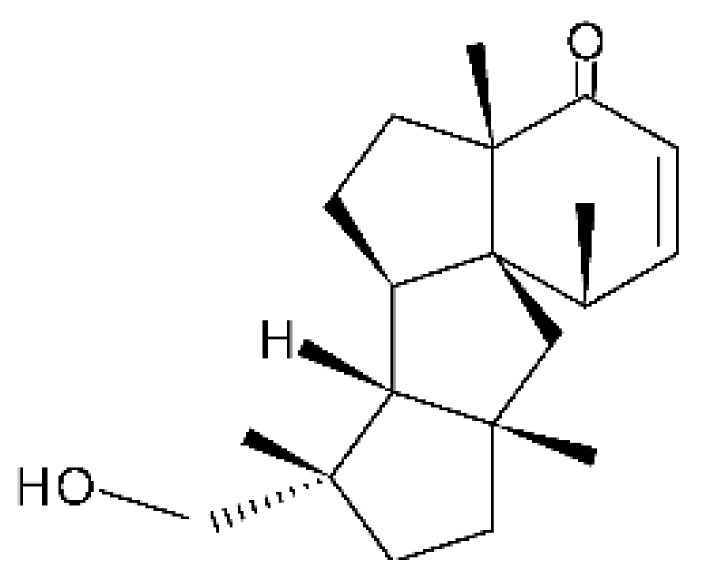	*E. coli* *P. aeruginosa* *Salmonella typhimurium*	25 µg/mL 12.5 µg/mL 100 µg/mL	[67]
*Leptosphaeria* sp. XL026 (leaves)	Cyclopiane-type diterpene	**(36)** Conidiogenone G—C_20_H_30_O_2_/303.231857 [M + H]^+^	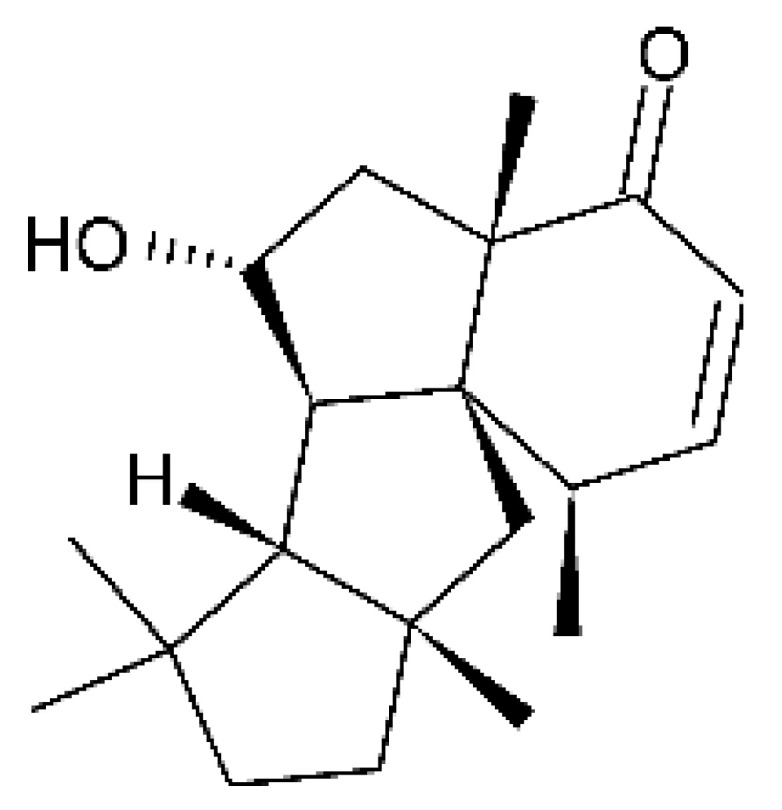	*E. coli* *P. aeruginosa* *Salmonella typhimurium*	50 µg/mL 25 µg/mL >100 µg/mL	[67]
*Chaetomium globosum* NM0066 (leaves)	Cytochalasan alkaloids	**(37)** Chaetoglobosin Vb—C_32_H_36_N_2_O_5_/529.2709 [M + H]^+^	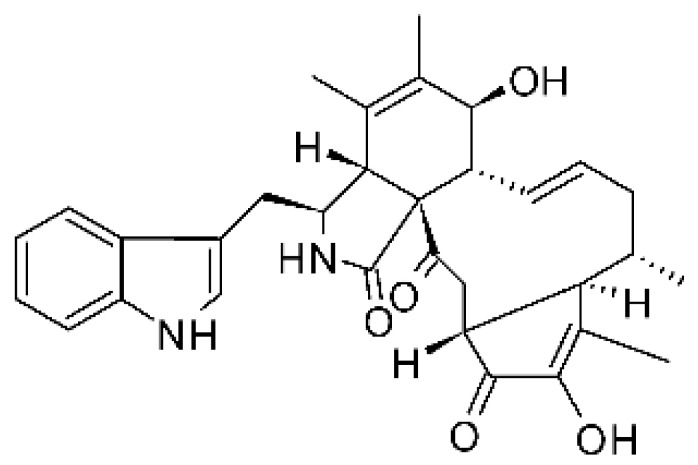	*E. coli* *P. aeruginosa*	>100 μg/mL >100 µg/mL	[68]
*C. globosum* NM0066 (leaves)	Cytochalasan alkaloids	**(38)** Chaetoglobosin V—C_32_H_36_N_2_O_5_/447.2507 [M]^+^	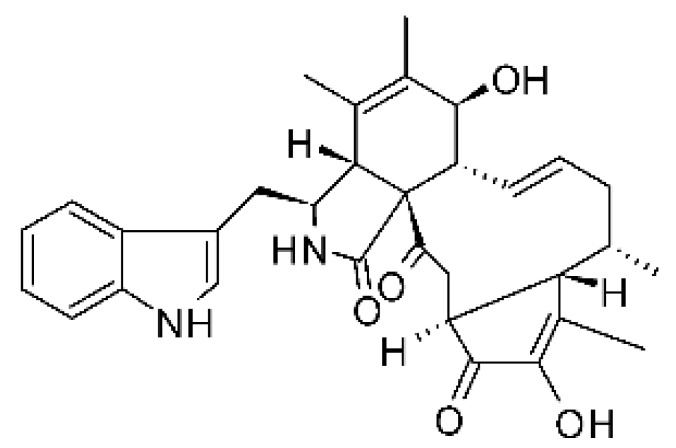	*E. coli* *P. aeruginosa*	>100 µg/mL 50 µg/mL	[68]
*C. globosum* NM0066 (leaves)	Cytochalasan alkaloids	**(39)** Chaetoglobosin G—C_32_H_38_N_2_O_5_/531.285349 [M + H]^+^	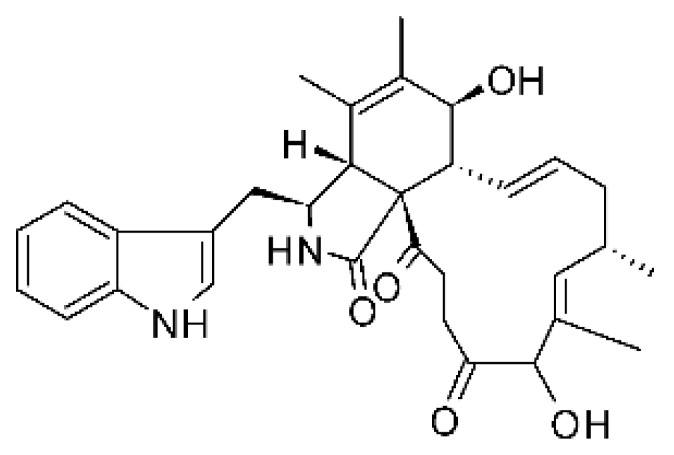	*E. coli* *P. aeruginosa*	> 100 μg/mL 50 µg/mL	[68]
*Daldinia eschscholtzii* HJ001 (mangrove)	Cytochalasin	**(40)** [11]-cytochalasa5(6), 13-diene-1,21-dione-7,18-dihydroxy 16,18-dimethyl-10-phenyl -(7S*,13E,16S*,18R*)—C_29_H_41_NO_4_/452.2791 [M + H]^+^	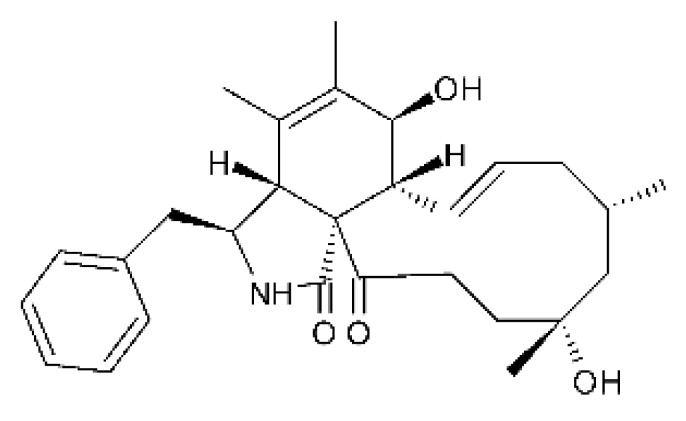	*E. coli* ATCC 25922 *Vibrio parahaemolyticus* ATCC 17802 *Vibrio alginolyticus* ATCC 17749	50 µg/mL 50 µg/mL 50 µg/mL	[69]
*Phomopsis* sp. CAM240 (seed)	Cytochalasins	**(41)** 18-metoxycytochalasin J—C_29_H_39_NO_4_/466.29587 [M + H]^+^	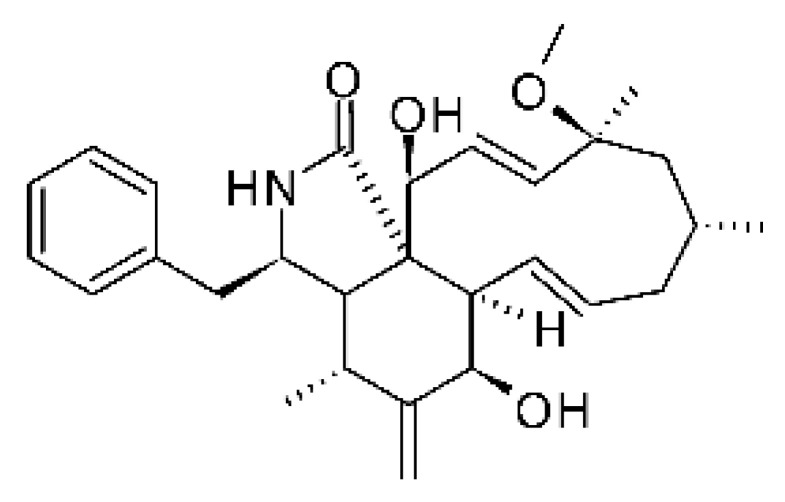	*Vibrio cholerae NB2* *Vibrio cholerae PC2* *Shigella flexneri SDINT*	512 μg/mL >512 μg/mL 128 μg/mL	[70]
*Phomopsis* sp. CAM240 (seed)	Cytochalasins	**(42)** Cytochalasins H—C_30_H_39_NO_5_/494.28949 [M + H]^+^	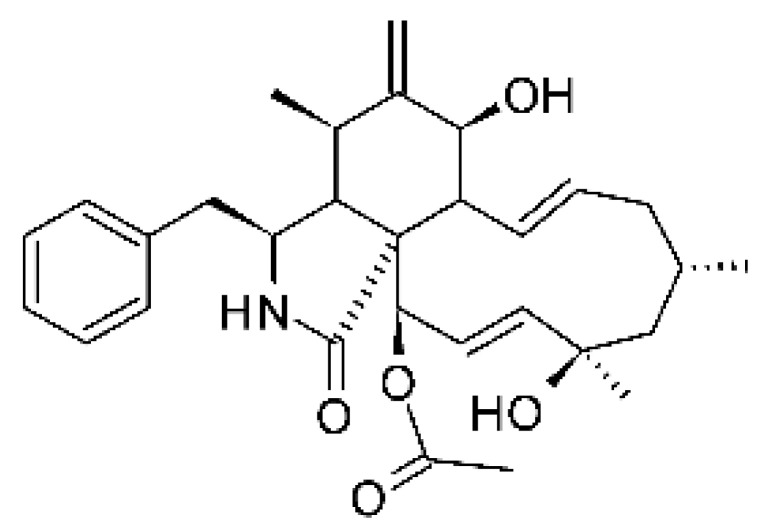	*Vibrio cholerae NB2* *Vibrio cholerae PC2* *Shigella flexneri SDINT*	512 μg/mL 256 μg/mL 128 μg/mL	[70]
*Phomopsis* sp. CAM240 (seed)	Cytochalasins	**(43)** Cytochalasins J—C_28_H_37_NO_4_/452.28052 [M + H]^+^	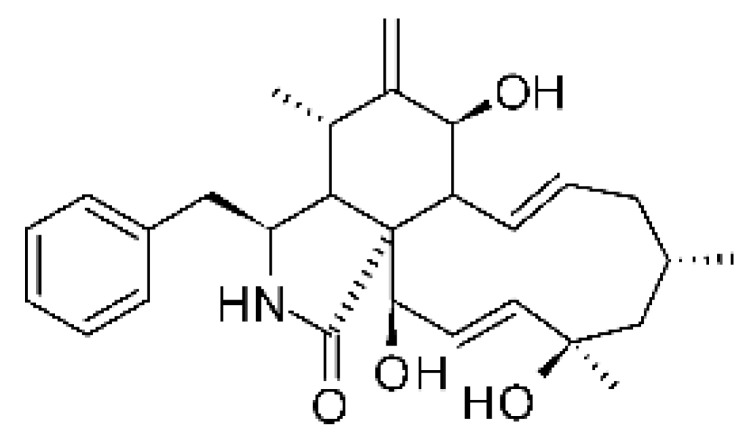	*Vibrio cholerae NB2* *Vibrio cholerae PC2* *Shigella flexneri SDINT*	512 μg/mL >512 μg/mL 128 μg/mL	[70]
*Aspergillus flavipes* Y-62 (stems)	Cytochalasin derivatives	**(44)** Cytochalasin Z16—C_28_H_31_NO_5_/462.2275 [M + H]^+^	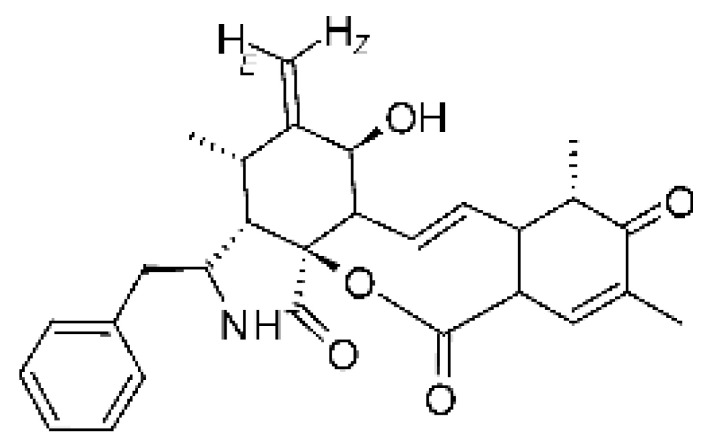	*E. coli CMCC(B) 44102* *K.pneumoniae CMCC(B) 46117* *P. aeruginosa* CMCC(B) 10104	32 μg/mL 32–64 μg/mL 32 μg/mL	[64]
*A. flavipes* Y-62 (stems)	Cytochalasin derivatives	**(45)** Cytochalasin Z7—C_28_H_33_NO_5_/464.24315 [M + H]^+^	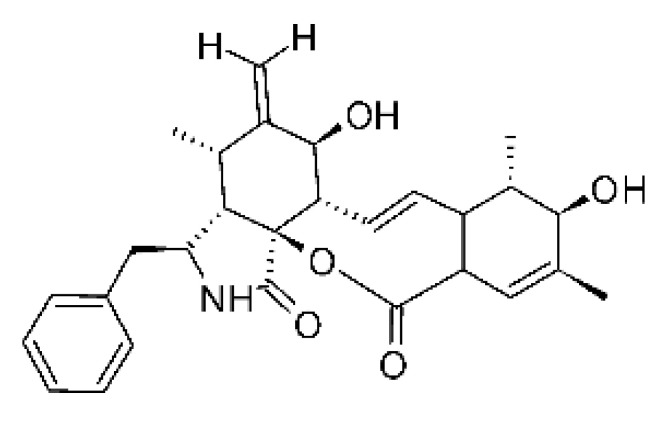	*E. coli* CMCC(B) 44102 *K. pneumoniae* CMCC(B) 46117 *P. aeruginosa* CMCC(B) 10104	128 μg/mL 64 μg/mL 64 μg/mL	[64]
*A. flavipes* Y-62 (stems)	Cytochalasin derivatives	**(46)** Cytochalasin Z17—C_28_H_33_NO_5_/464.24315 [M + H]^+^	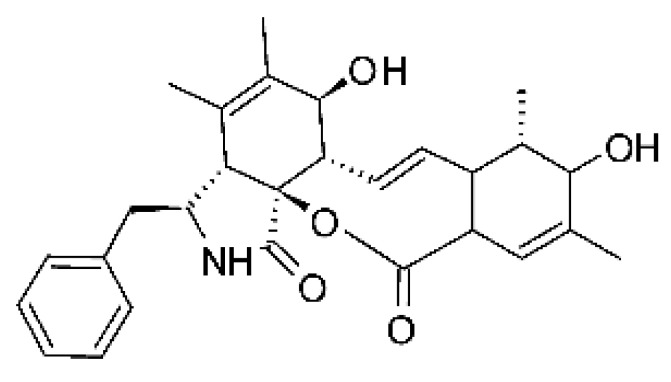	*E. coli* CMCC(B) 44102 *K.pneumoniae* CMCC(B) 46117 *P. aeruginosa* CMCC(B) 10104	16 μg/mL 32 μg/mL 32 μg/mL	[64]
*A. flavipes* Y-62 (stems)	Cytochalasin derivatives	**(47)** Rosellichalasin—C_28_H_31_NO_5_/462.2275 [M + H]^+^	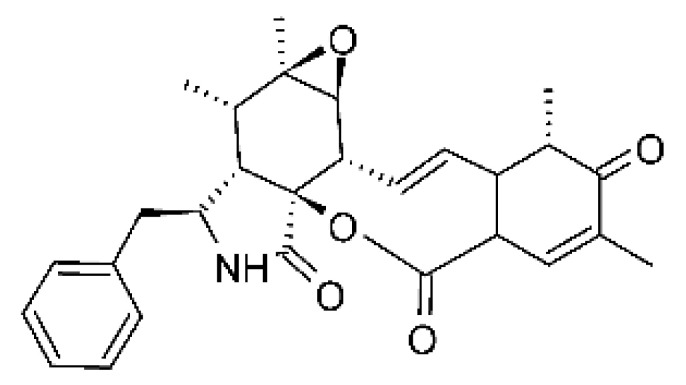	*E. coli* CMCC(B) 44102 *K. pneumoniae* CMCC(B) 46117 *P. aeruginosa* CMCC(B) 10104	32 μg/mL 32 µg/mL 32 µg/mL	[64]
*A. flavipes* Y-62 (stems)	Cytochalasin derivatives	**(48)** Cytochalasin Z13—C_25_H_33_NO_5_/428.24315 [M + H]^+^	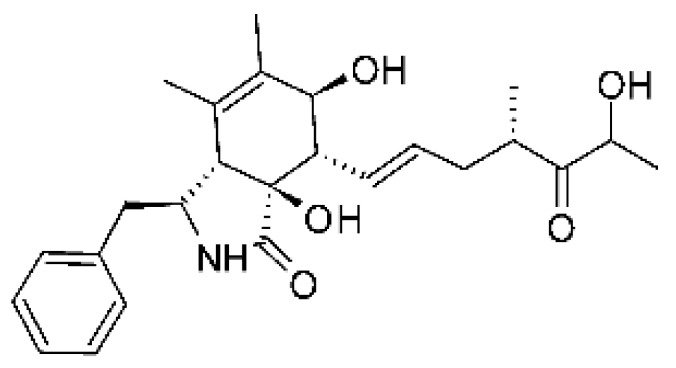	*E. coli* CMCC(B) 44102	64 µg/mL	[64]
*A. flavipes* Y-62 (stems)	Cytochalasin derivatives	**(49)** Dipeptide aspergillazine A—C_20_H_22_N_2_O_8_S/451.116962 [M + H]^+^	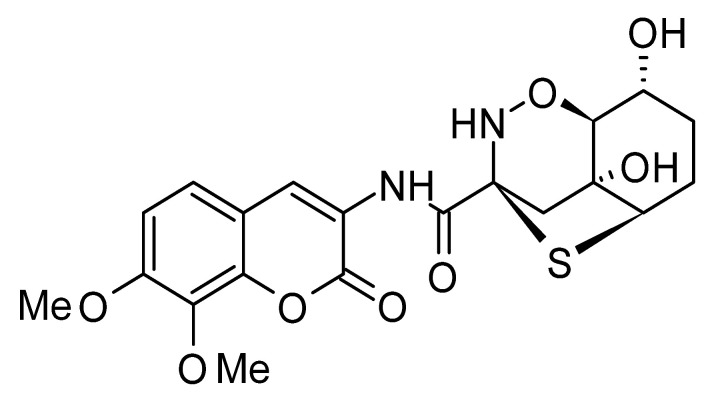	*P. aeruginosa* CMCC(B) 10104	64 µg/mL	[64]
*A. flavipes* Y-62 (stems)	Cytochalasin derivatives	**(50)** Flavipin—C_9_H_8_O_5_/197.04445 [M + H]^+^	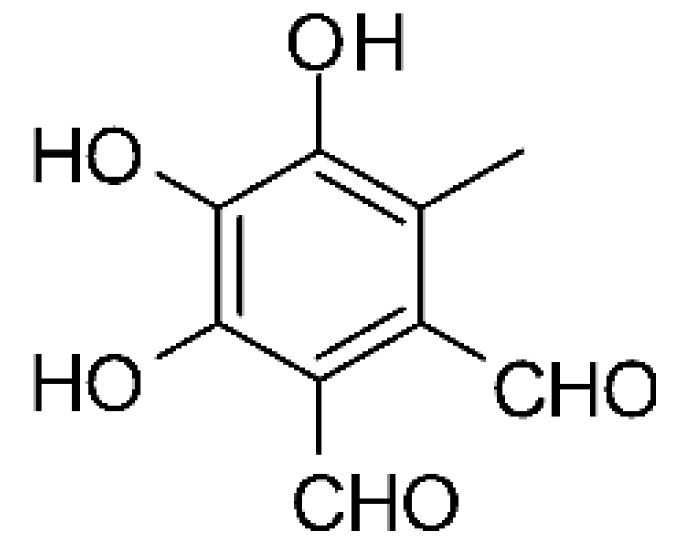	*E. coli* CMCC(B) 44102	64 µg/mL	[64]
*A. flavipes* Y-62 (stems)	Cytochalasin derivatives	**(51)** N-benzoyl-L-phenyalaninol—C_16_H_17_NO_2_/256.133205 [M + H]^+^	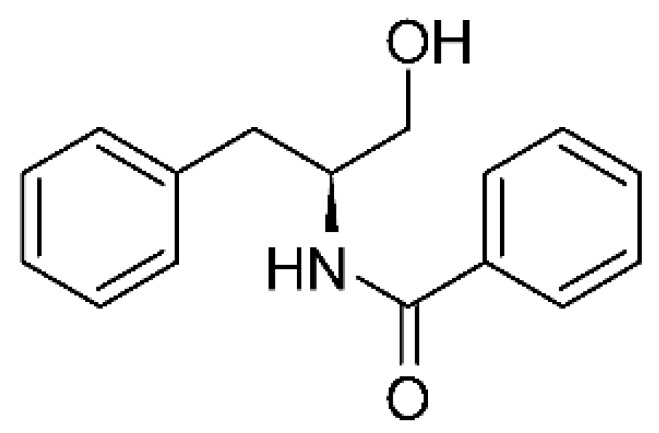	*E.coli* CMCC(B) 44102 *K. pneumoniae* CMCC(B) 46117 *P. aeruginosa* CMCC(B) 10104	64 µg/mL 64 µg/mL 64 µg/mL	[64]
*Eupenicillium* sp. LG41 (roots)	Decalin polyketides	**(52)** Eupenicinicol A—C_19_H_30_O_4_/323.2219 [M + H]^+^	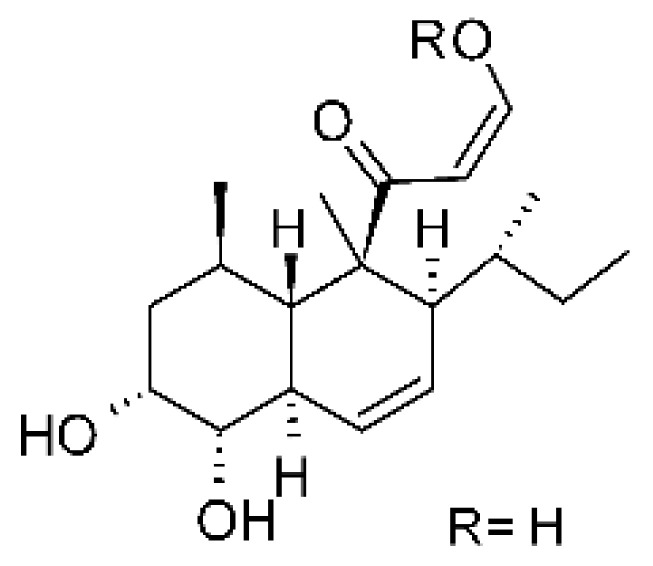	*Acinetobacter* sp. BD4 DSM 586 *E. coli* DSM 1116	10 µg/mL 5.0 µg/mL	[71]
*Eupenicillium* sp. LG41 (roots)	Decalin polyketides	**(53)** Eupenicinicol B—C_20_H_32_O_4_/337.2373 [M + H]^+^	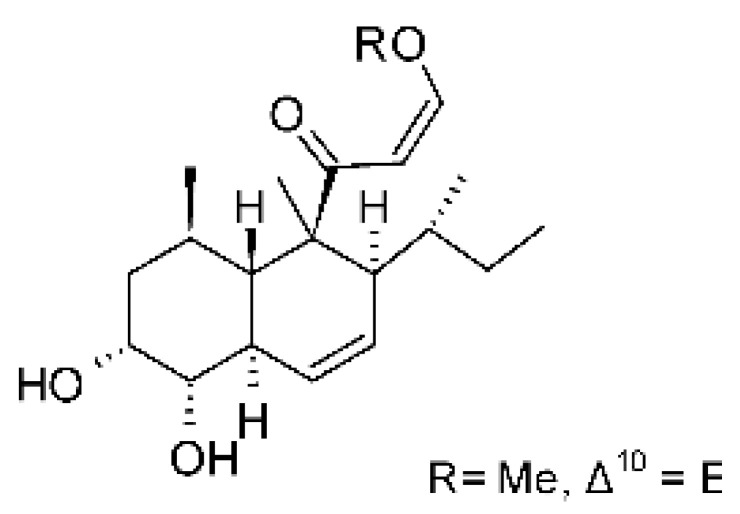	*Acinetobacter* sp. BD4 DSM 586 *E. coli* DSM 1116	>10 μg/mL 5.0 µg/mL	[71]
*F. proliferatum* AF-04 (onion)	Depsipeptide	**(54)** Beauvericin—C_45_H_59_N_3_O_9_/786.432407 [M + H]^+^	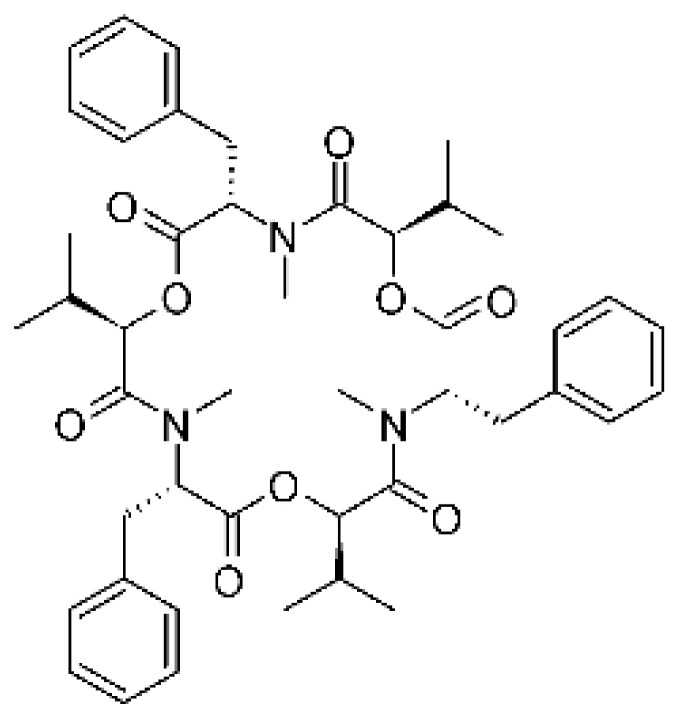	*E. coli*	>100 μg/mL	[55]
*Fusarium solani* JK10 (roots)	7–desmethyl fusarin C derivatives	**(55)** NG-391—C_21_H_27_NO_6_/ 390.1911 [M + H]^+^	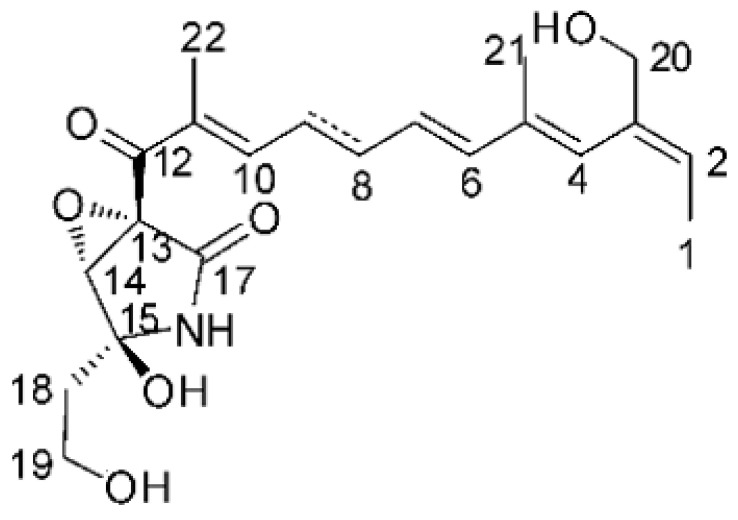	*Acinetobacter* sp. DSM 586 *E. coli* DSM 1116	>10.0 µg/mL 10.0 µg/mL	[72]
*F. solani* JK10 (roots)	7–desmethyl fusarin C derivatives	**(56)** NG-393—C_21_H_27_NO_6_/390.1911 [M + H]^+^	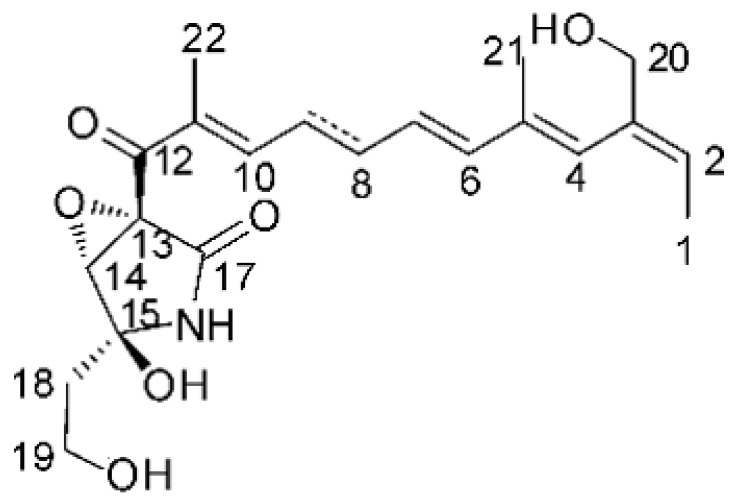	*Acinetobacter* sp. DSM 586 *E. coli* DSM 1116	>10.0 µg/mL 10.0 µg/mL	[72]
*F. solani* JK10 (roots)	7–desmethyl fusarin C derivatives	**(57)** 406.1860 [M + H]^+^ C_21_H_27_NO_7_	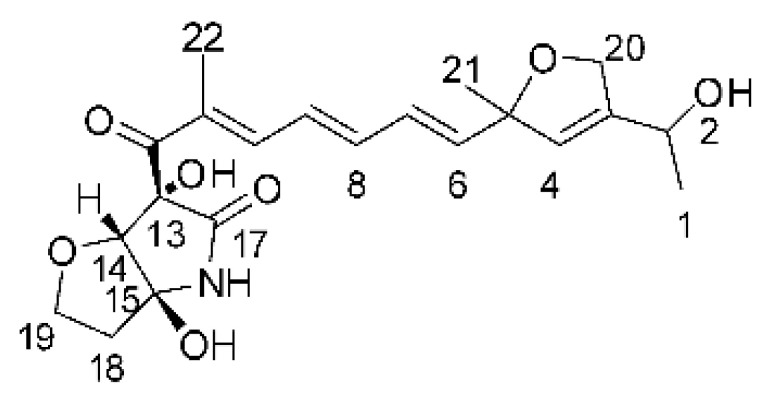	*Acinetobacter* sp. DSM 586 *E. coli* DSM 1116	10.0 µg/mL 10.0 µg/mL	[72]
*F. solani* JK10 (roots)	7–desmethyl fusarin C derivatives	**(58)** 406.1860 [M + H]^+^ C_21_H_27_NO_7_	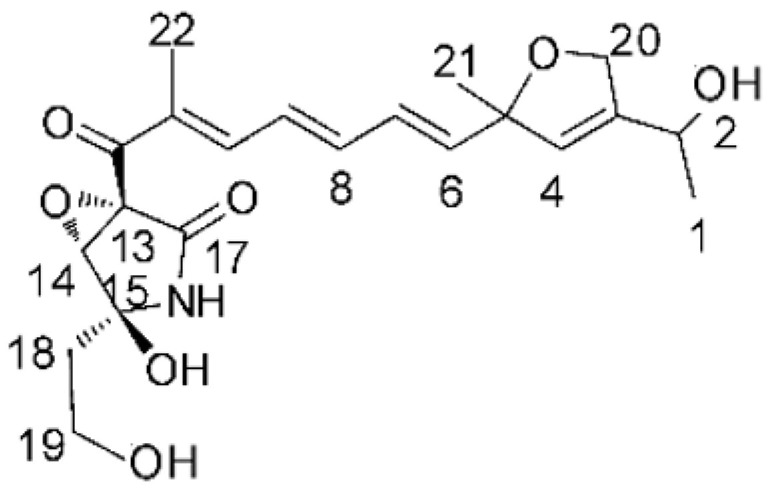	*Acinetobacter* sp. DSM 586 *E. coli* DSM 1116	10.0 µg/mL 10.0 µg/mL	[72]
*F. solani* JK10 (roots)	7–desmethyl fusarin C derivatives	**(59)** 420.1656 [M + H]^+^ C_21_H_25_NO_8_	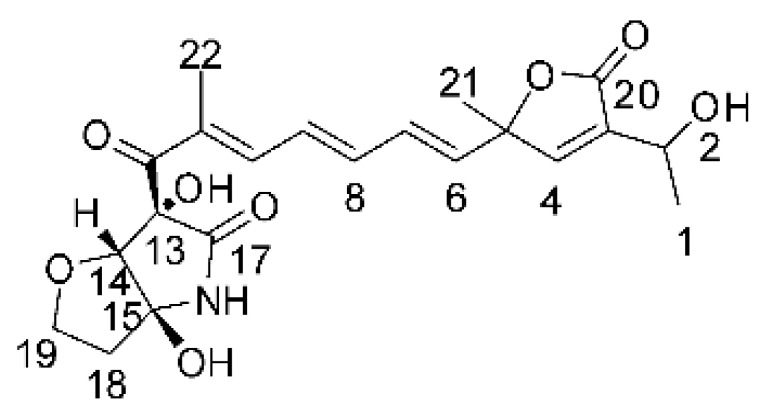	*Acinetobacter* sp. DSM 586 *E. coli* DSM 1116	>10.0 µg/mL 5 µg/mL	[72]
*F. solani* JK10 (roots)	7–desmethyl fusarin C derivatives	**(60)** 13α–hydroxylucilactaene—C_22_H_29_NO_7_/420.2017 [M + H]^+^	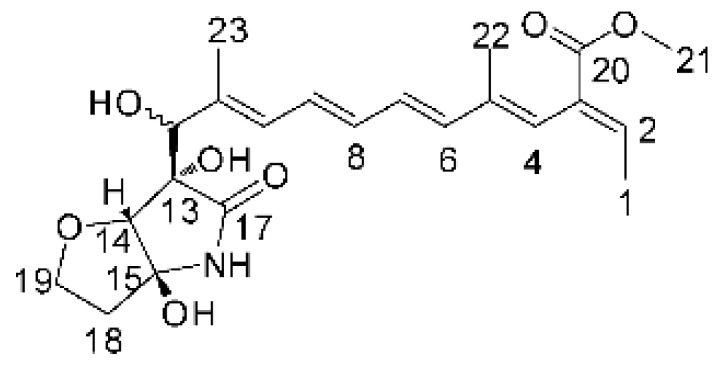	*Acinetobacter* sp. DSM 586 *E. coli* DSM 1116	10.0 µg/mL 10.0 µg/mL	[72]
*F. solani* JK10 (roots)	7–desmethyl fusarin C derivatives	**(61)** 388.2118 [M + H]^+^ C_22_H_29_NO_5_	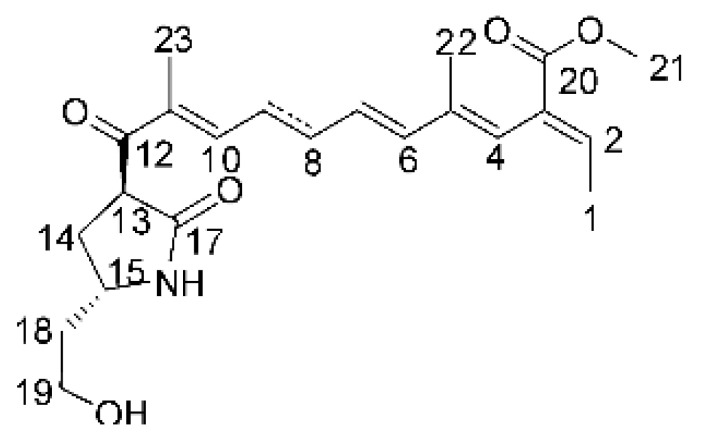	*Acinetobacter* sp. DSM 586 *E. coli* DSM 1116	>10.0 µg/mL 5 µg/mL	[72]
*Bionectria* sp. Y1085	Diketopiperazine	**(62)** Bionectin D—C16H19N3O3S—333 [M]^+^	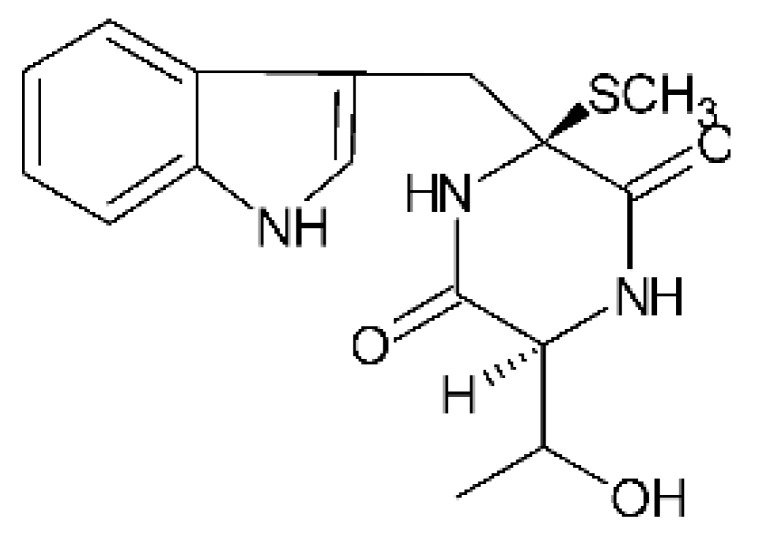	*E. coli* *Salmonella typhimurium* ATCC 6539	25 µg/mL 25 µg/mL	[73]
*Bionectria* sp. Y1085	Diketopiperazine	**(63)** Bionectin E—C_24_H_22_N_4_O_3_S_2_/501 [M+Na]^+^	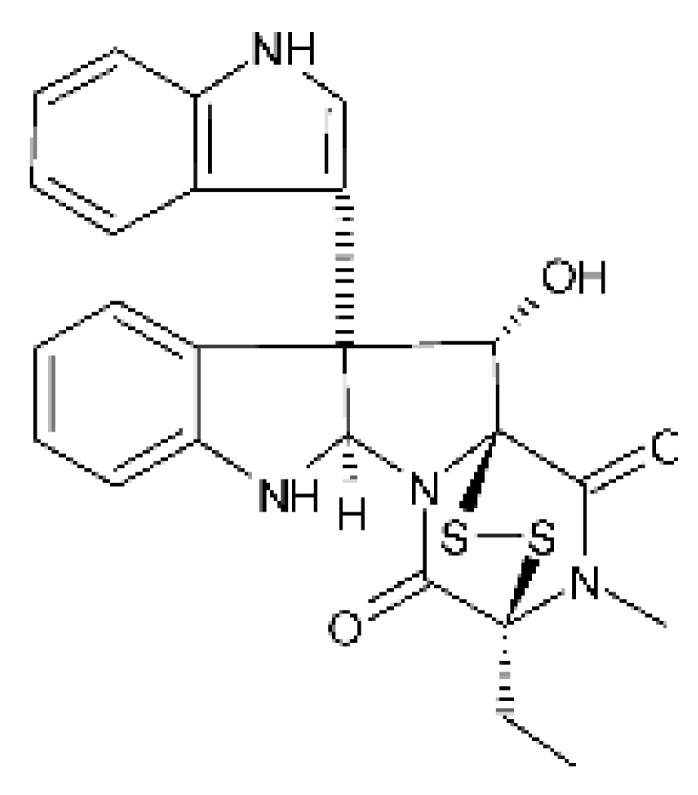	*E. coli* *S. typhimurium* ATCC 6539	>200 µg/mL >200 µg/mL	[73]
*Bionectria* sp. Y1085	Diketopiperazine	**(64)** Verticillin A—C_30_H_28_N_6_O_6_S_4_/697.102589 [M + H]^+^	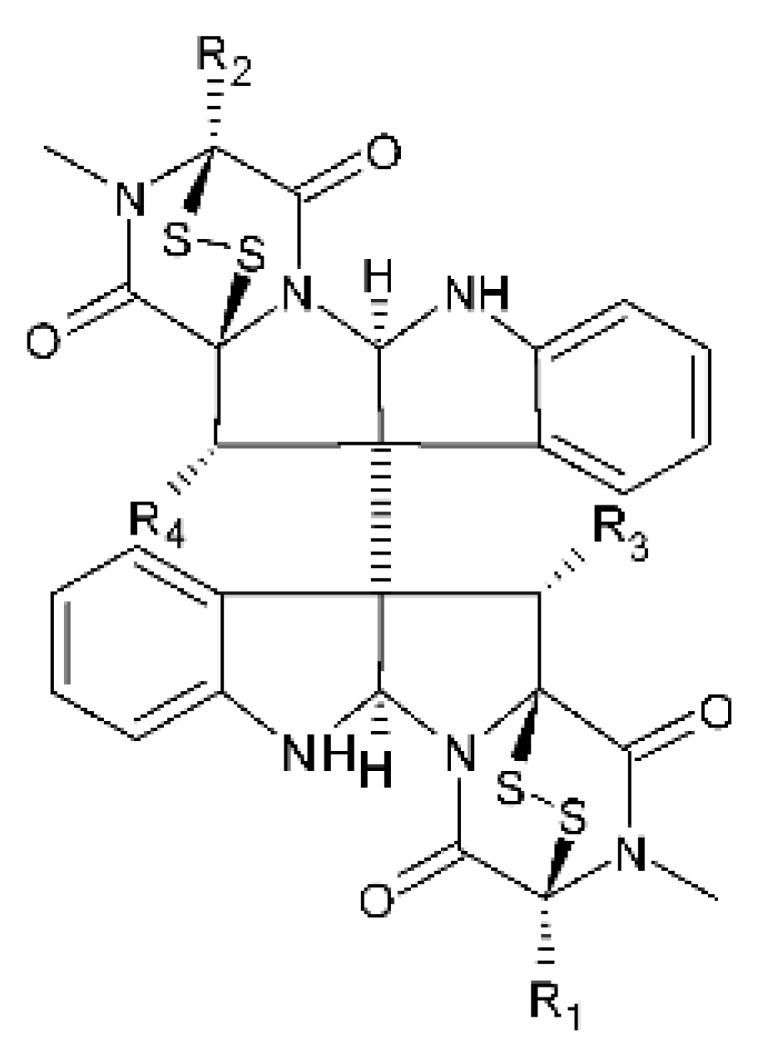	*E. coli* *S. typhimurium* ATCC 6539	12.5 µg/mL 12.5 µg/mL	[73]
*Bionectria* sp. Y1085	Diketopiperazine	**(65)** sch 52901—C_31_H_30_N_6_O_6_S_4_/711.118239 [M + H]^+^	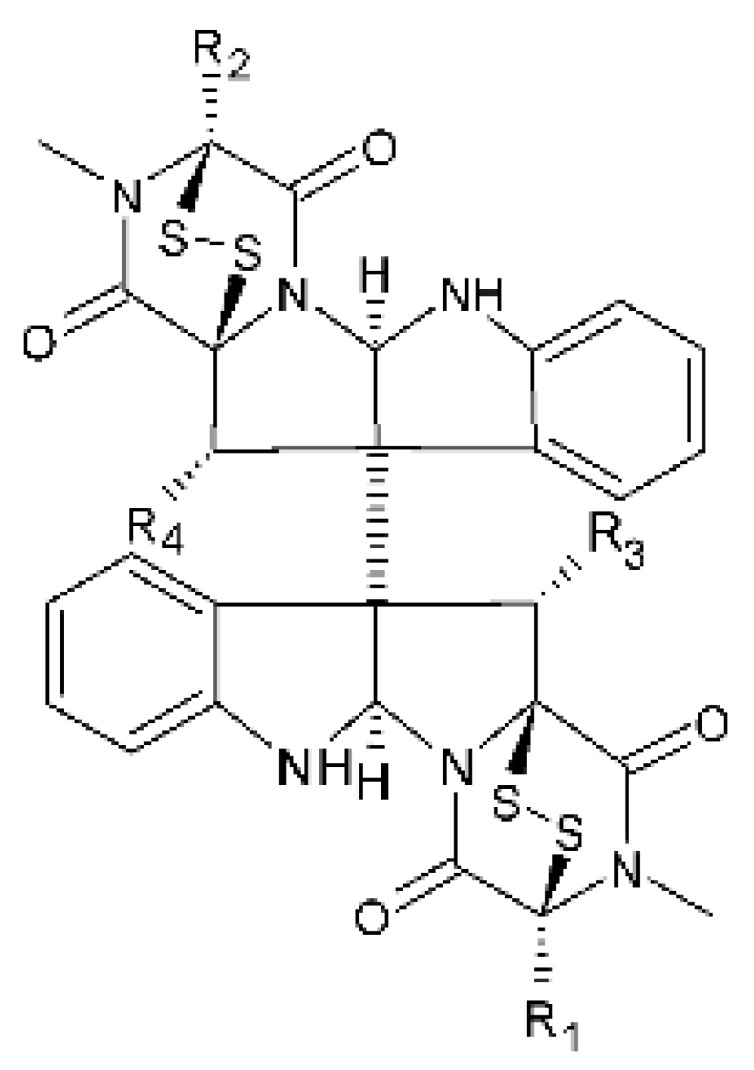	*E. coli* *S. typhimurium* ATCC 6539	6.25 µg/mL 6.25 µg/mL	[73]
*Bionectria* sp. Y1085	Diketopiperazine	**(66)** Gliocladicillin C—C_32_H_32_N_6_O_7_S_4_/741.128804 [M + H]^+^	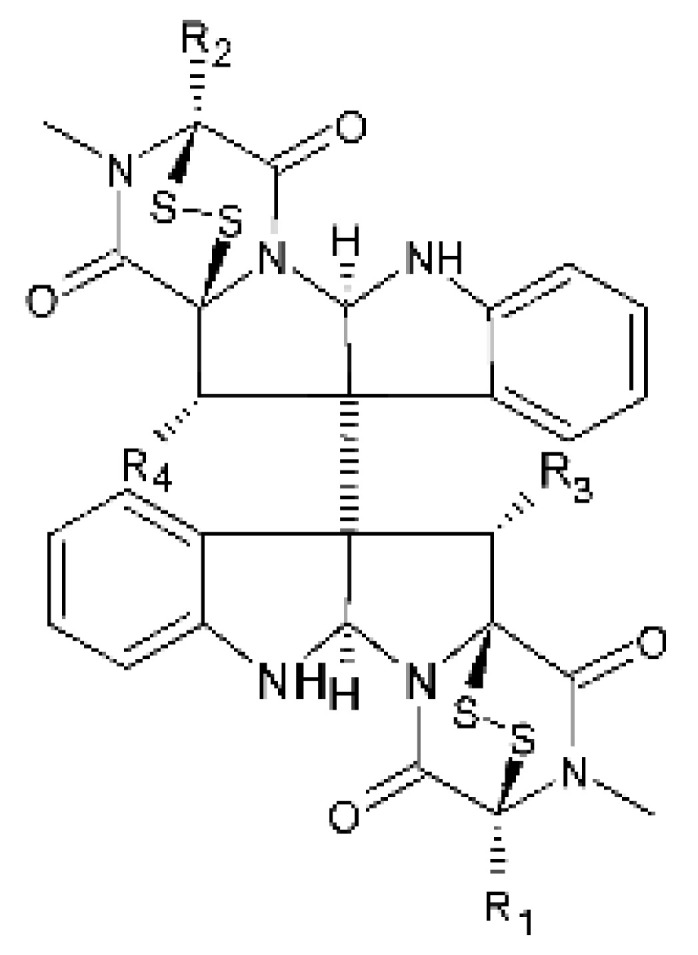	*E. coli* *S. typhimurium* ATCC 6539	6.25 µg/mL 6.25 µg/mL	[73]
*Chaetomium* sp. M336	Epipolythiodioxopiperazine	**(67)** 6-formamide-chetomin—C_32_H_30_N_6_O_7_S_4_/761.0993 [M+Na]^+^	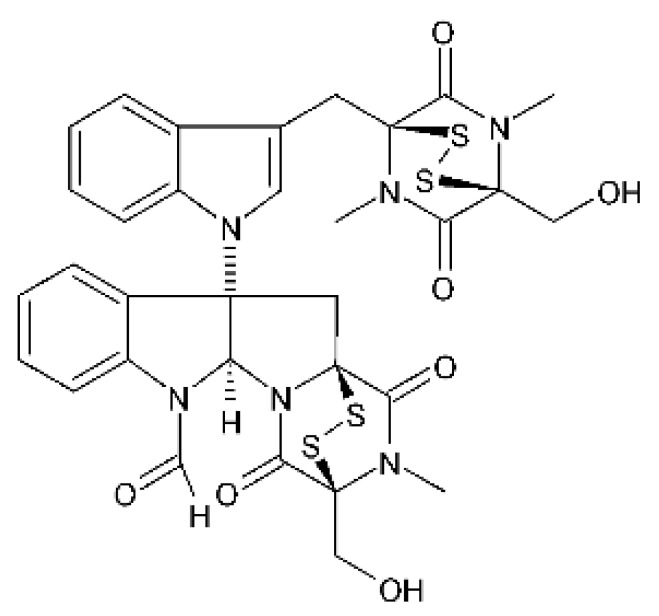	*E. coli* *S. typhimurium* ATCC 6539	0.78 µg/mL 0.78 µg/mL	[74]
*Diaporthe* sp. LG23 (leaves)	Ergosterol derivatives	**(68)** 3β,5α,9α-trihydroxy-(22E,24R)—ergosta-7,22-dien-6-one—C_28_H_44_O_4/_445.331236 [M + H]^+^	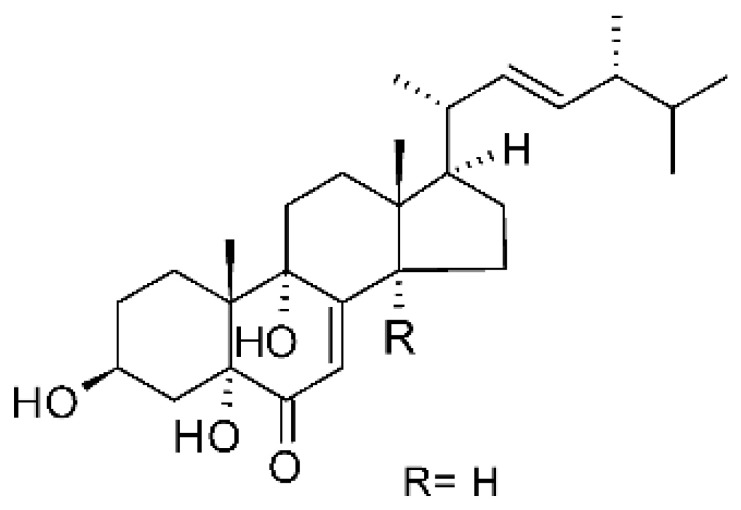	*Escherichia coli* DSM 682 *Pseudomonas aeruginosa* DSM 22644	>10 µg/mL >10 µg/mL	[75]
*Diaporthe* sp. LG23 (leaves)	Ergosterol derivatives	**(69)** 3β,5α,9α,14α-tetrahydroxy-(22E,24R)-ergosta-7,22-dien-6-one—C_28_H_44_O_5_/461.326151 [M + H]^+^	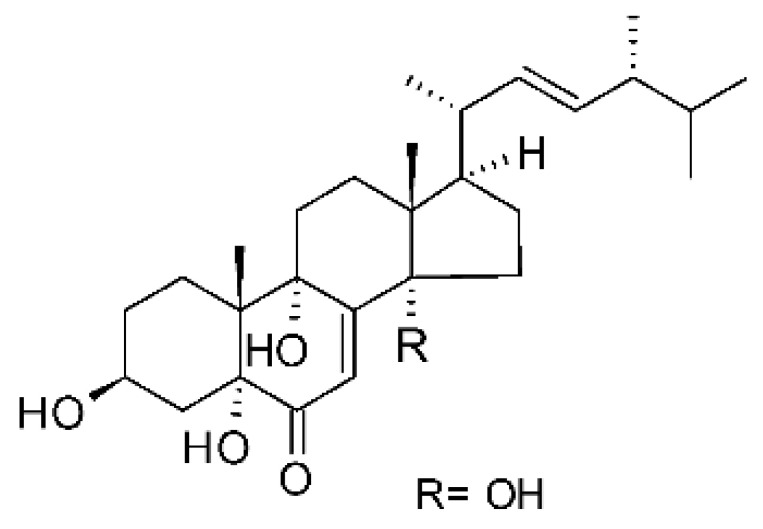	*E. coli* DSM 682 *P. aeruginosa* DSM 22644	>10 µg/mL >10 µg/mL	[75]
*Diaporthe* sp. LG23 (leaves)	Ergosterol derivatives	**(70)** (22E,24R)-ergosta-7,9(11),22-triene-3β,5α,6α-triol—C_28_H_44_O_3_/429.336322 [M + H]^+^	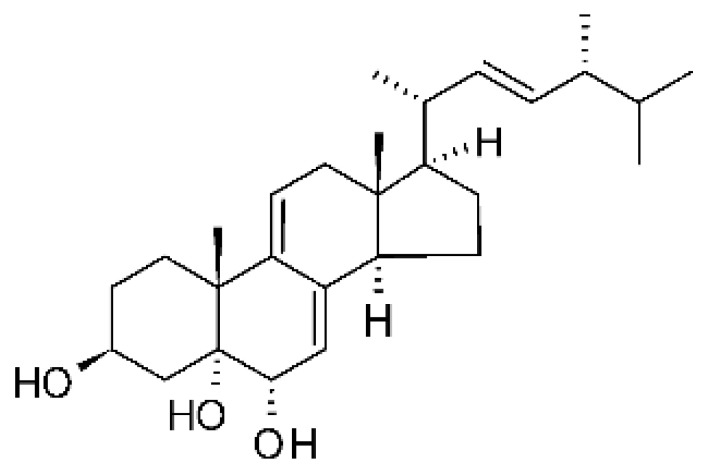	*E. coli* DSM 682 *P. aeruginosa* DSM 22644	>10 µg/mL >10 µg/mL	[75]
*Diaporthe* sp. LG23 (leaves)	Ergosterol derivatives	**(71)** Chaxine C—C_28_H_40_O_4_/441.299936 [M + H]^+^	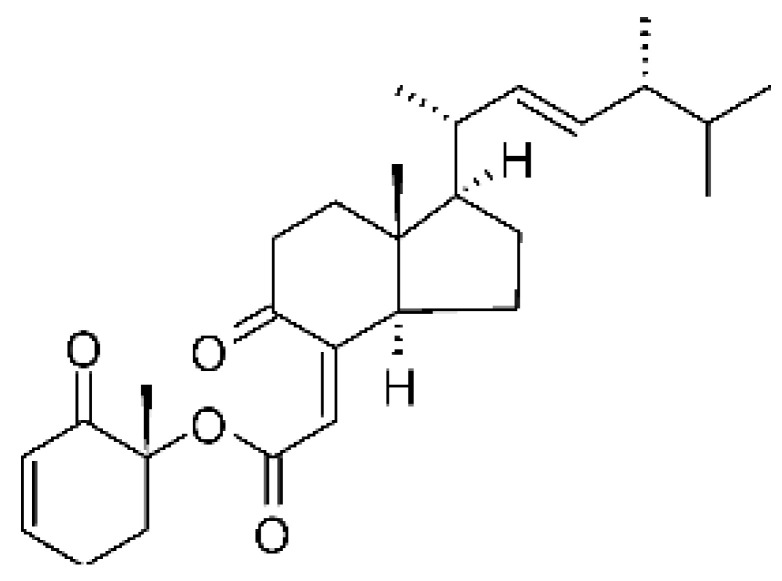	*E. coli* DSM 682 *P. aeruginosa* DSM 22644	>10 µg/mL >10 µg/mL	[75]
*Diaporthe* sp. LG23 (leaves)	Ergosterol derivatives	**(72)** Demethylincisterol A_3_—C_21_H_32_O_3_/333.242421 [M + H]^+^	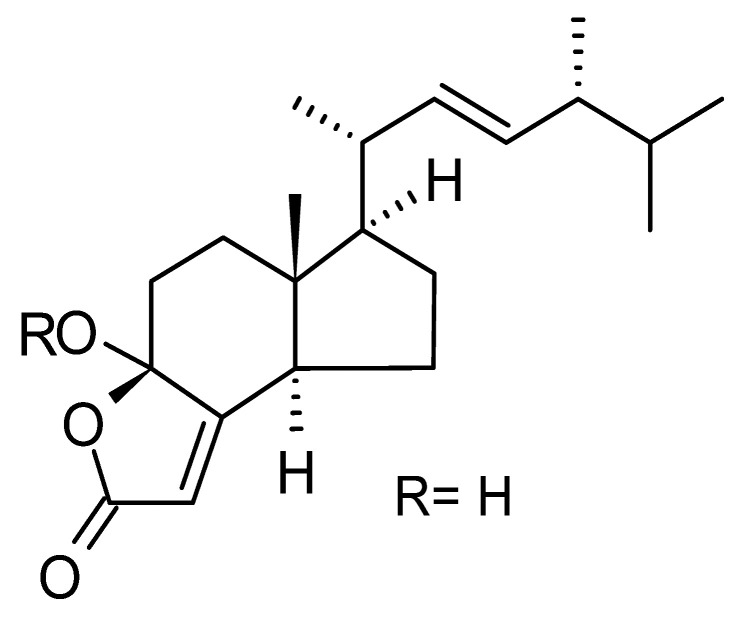	*E. coli* DSM 682 *P. aeruginosa* DSM 22644	>10 μg/mL >10 μg/mL	[75]
*Diaporthe* sp. LG23 (leaves)	Ergosterol derivatives	**(73)** Volemolide—C_22_H_34_O_3_/347.258071 [M + H]^+^	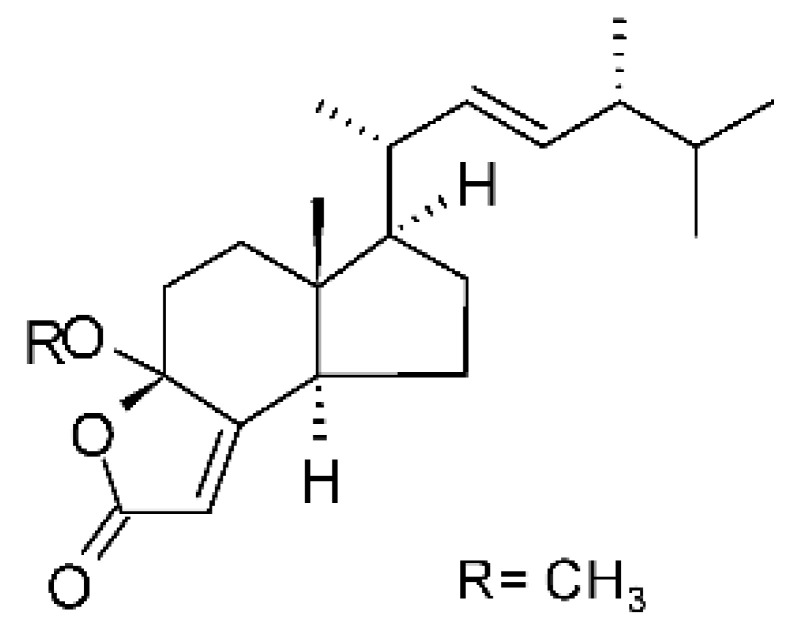	*E. coli* DSM 682 *P. aeruginosa* DSM 22644	>10 μg/mL >10 μg/mL	[75]
*Fusarium solani* HDN15-410 (root)	Fusaric acid	**(74)** Fusaric acid—C_10_H_13_NO_2_/180.101905 [M + H]^+^	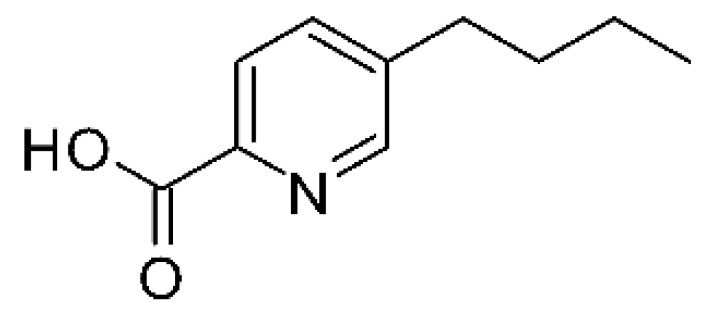	*P. aeruginosa* *Vibrio parahaemolyticus*	35.8 μg/mL >200 μg/mL	[76]
*F. solani* HDN15-410 (root)	Fusaric acid derivatives	**(75)** Fusaricates H—C_14_H_21_NO_3_/252.15942 [M + H]^+^	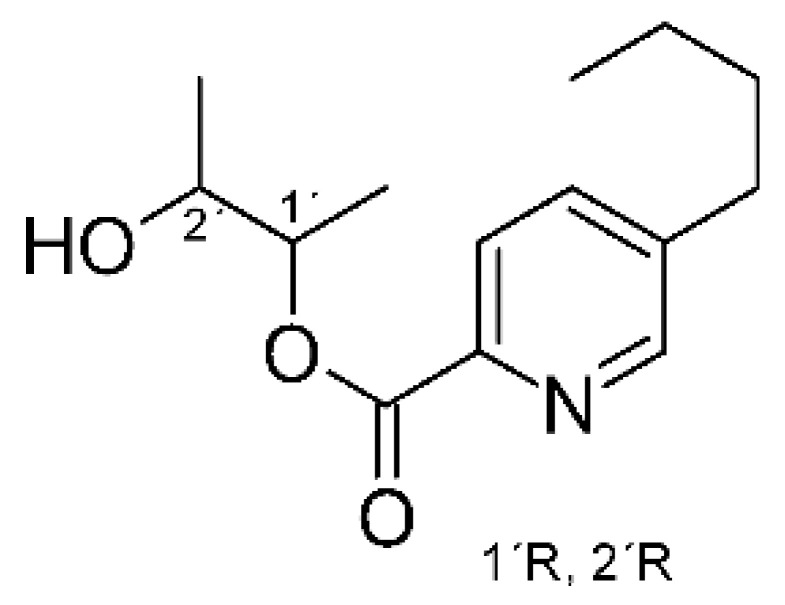	*P. aeruginosa* *V. parahaemolyticus*	>200 µg/mL >200 µg/mL	[76]
*F. solani* HDN15-410 (root)	Fusaric acid derivatives	**(76)** Fusaricates I—C_14_H_21_NO_3_/252.15942 [M + H]^+^	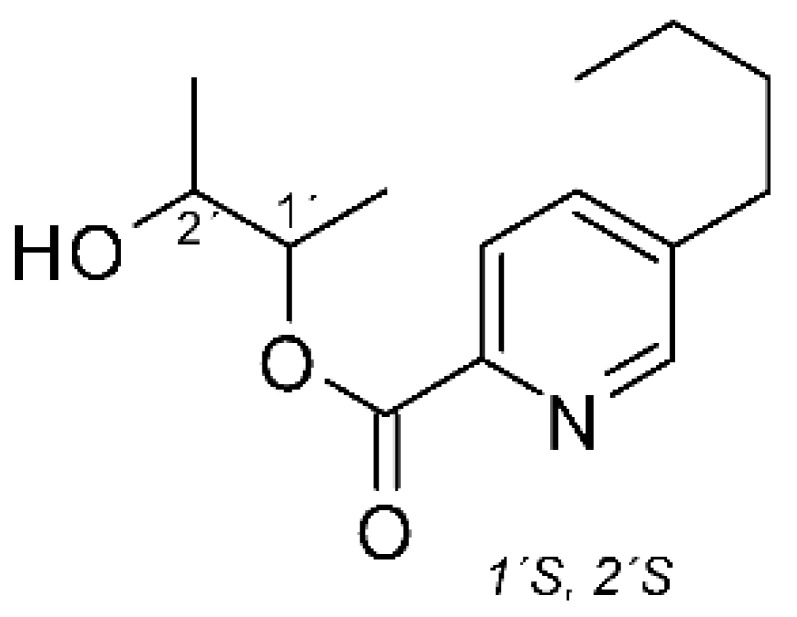	*P. aeruginosa* *V. parahaemolyticus*	>200 µg/mL >200 µg/mL	[76]
*Fusarium* sp. FL10 (leaves)	Helvolic acid derivative	**(77)** Helvolic acid methyl ester—C_34_H_46_O_8_ /583.3284 [M + H]^+^	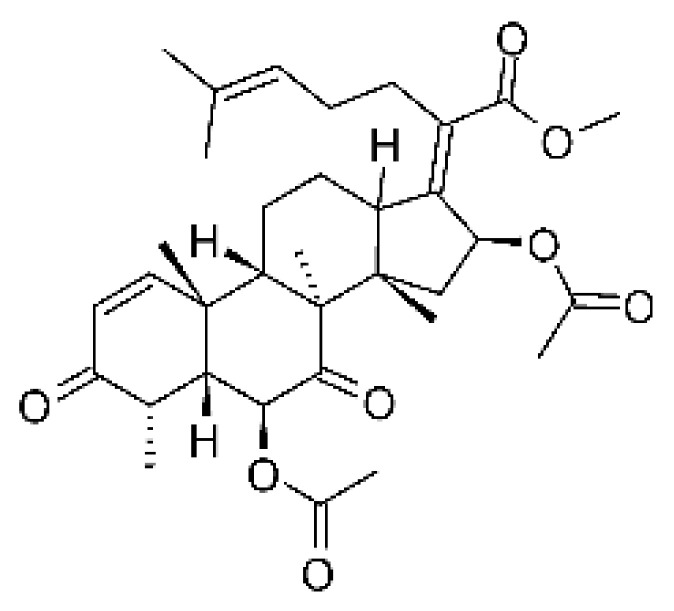	*E. coli* *P. aeruginosa*	6.25 µg/mL 3.13 µg/mL	[77]
*Fusarium* sp. FL10 (leaves)	Helvolic acid derivative	**(78)** Helvolic acid—C_33_H_44_O_8_/569.310895 [M + H]^+^	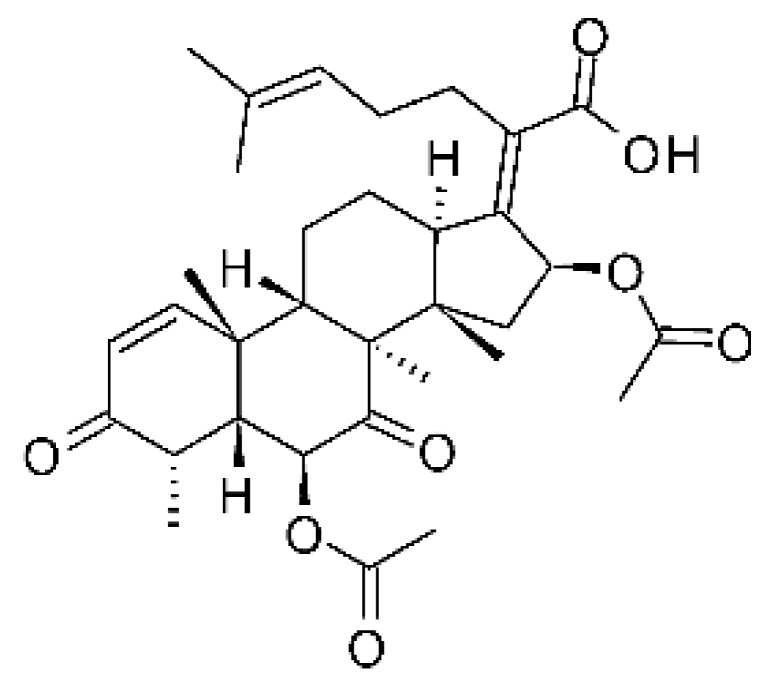	*E. coli* *P. aeruginosa*	6.25 µg/mL 3.13 μg/mL	[77]
*Fusarium* sp. FL10 (leaves)	Helvolic acid derivative	**(79)** Hydrohelvolic acid—C_33_H_46_O_9_/587.321459 [M + H]^+^	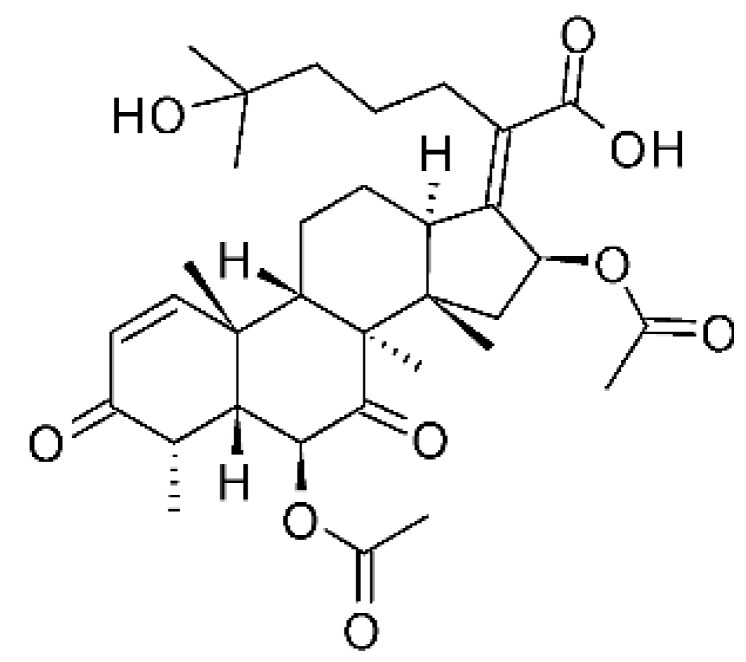	*E. coli* *P. aeruginosa*	6.25 µg/mL 3.13 µg/mL	[77]
*Aspergillus flavipes* Y-62 (stems)	Indene derivative	**(80)** methyl 2-(4-hydroxybenzyl)-1,7-dihydroxy-6-(3-methylbut-2-enyl)-1H-indene-1-carboxylate—C_23_H_24_O_5_/379.1554 [M-H]^−^	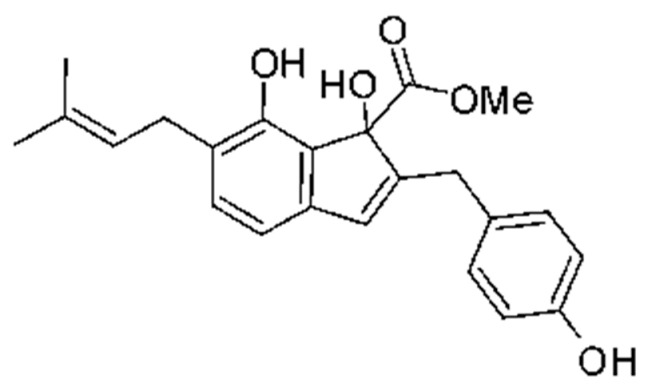	*Klebsiella pneumoniae* CMCC(B) 46117 *P. aeruginosa* CMCC(B) 10104	32 µg/mL 32 µg/mL	[64]
*Eurotium cristatum* EN-220 (marine alga)	Indole alkaloids	**(81)** Cristatumin A—C_19_H_21_N_3_O_3_/340.165568 [M + H]^+^	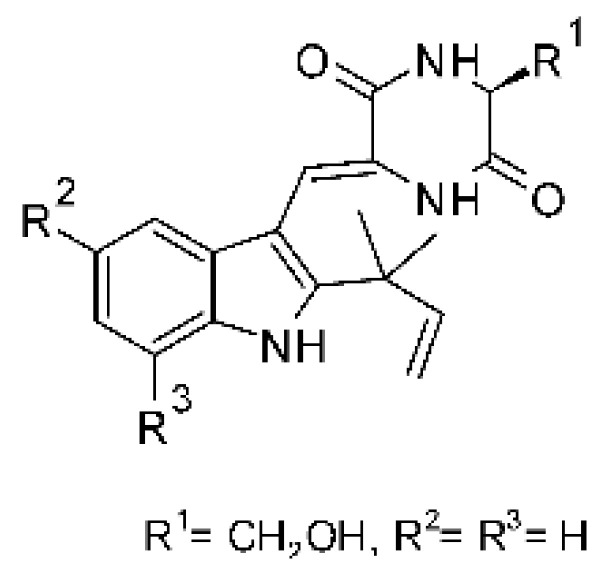	*E. coli*	64 μg/mL	[78]
*E. cristatum* EN-220 (marine alga)	Indole alkaloids	**(82)** Cristatumin B—C_31_H_41_N_3_O_3_/ 504.322069 [M + H]^+^	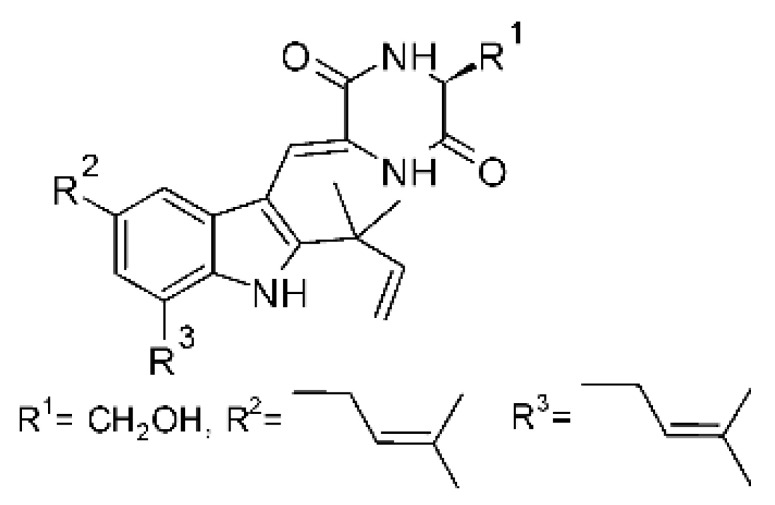	*E. coli*	64 μg/mL	[78]
*E. cristatum* EN-220 (marine alga)	Indole alkaloids	**(83)** Cristatumin C—C_30_H_32_N_6_O_4_/541.25578 [M + H]^+^	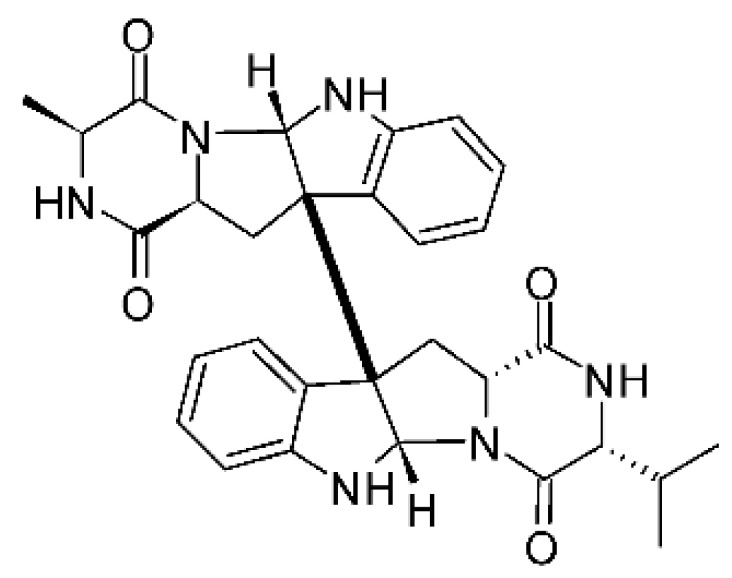	*E. coli*	64 μg/mL	[78]
*E. cristatum* EN-220 (marine alga)	Indole alkaloids	**(84)** Cristatumin D—C_19_H_21_N_3_O_4_/356.160483 [M + H]^+^	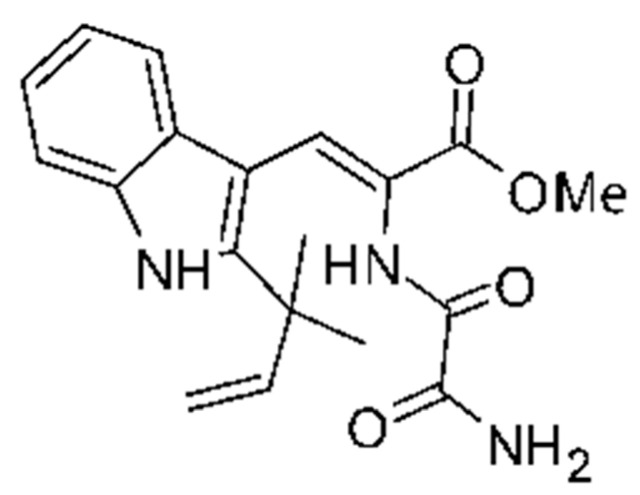	*E. coli*	64 μg/mL	[78]
*E. cristatum* EN-220 (marine alga)	Indole alkaloids	**(85)** Neoechinulin A—C_19_H_21_N_3_O_2_/324.170653 [M + H]^+^	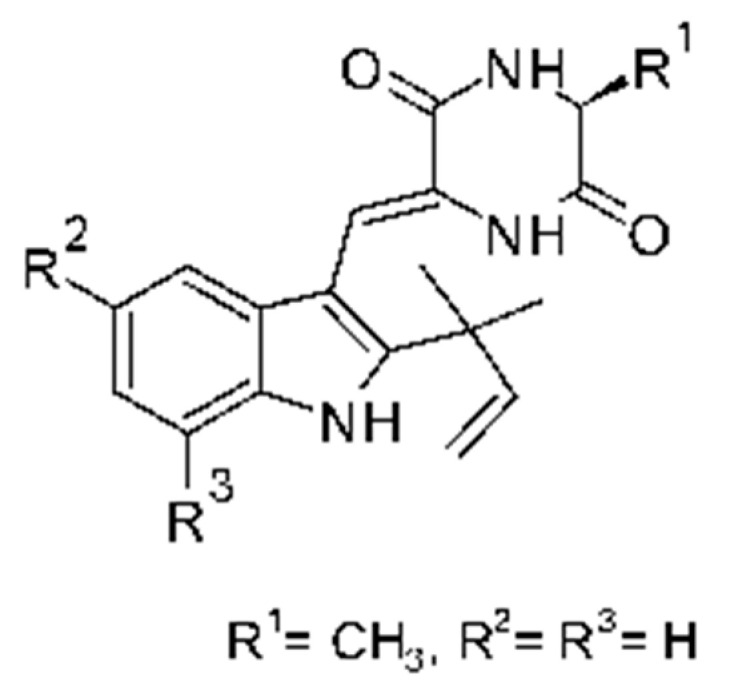	*E. coli*	64 μg/mL	[78]
*E. cristatum* EN-220 (marine alga)	Indole alkaloids	**(86)** Isoechinulin A—C_25_H_31_N_3_O_2_/406.248904 [M + H]^+^	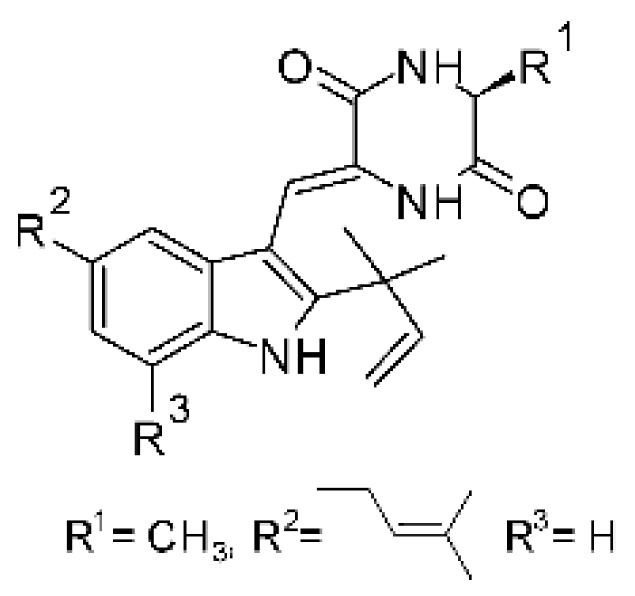	*E. coli*	64 μg/mL	[78]
*E. cristatum* EN-220 (marine alga)	Indole alkaloids	**(87)** Variecolorin G—C_25_H_31_N_3_O_2_/406.248904 [M + H]^+^	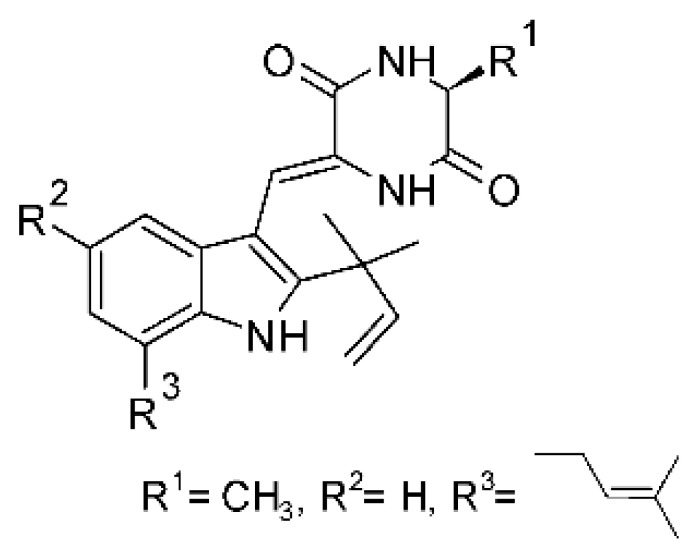	*E. coli*	64 μg/mL	[78]
*E. cristatum* EN-220 (marine alga)	Indole alkaloids	**(88)** Preechinulin—C_19_H_23_N_3_O_2_/326.186303 [M + H]^+^	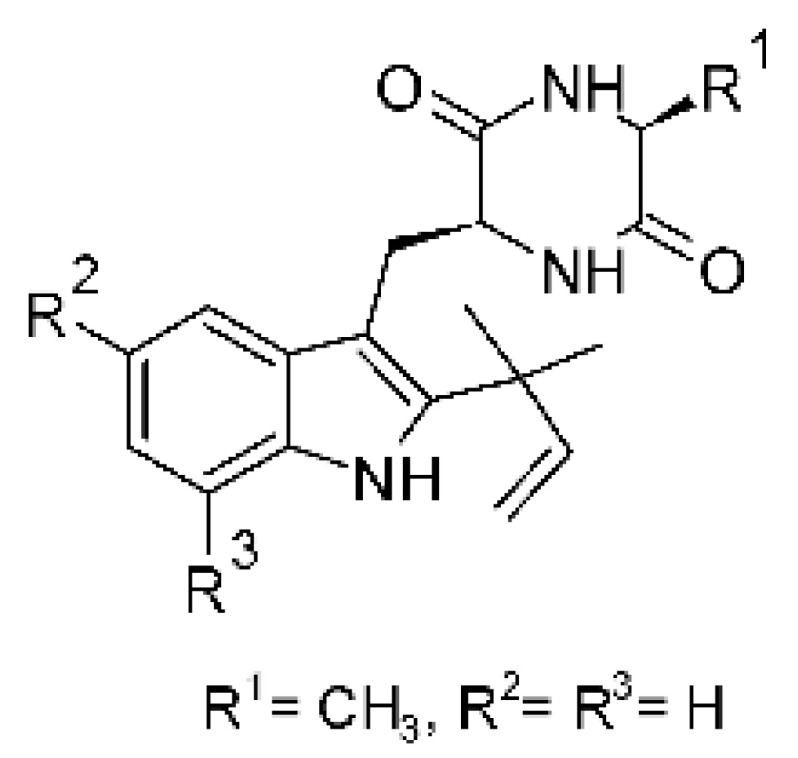	*E. coli*	64 µg/mL	[78]
*E. cristatum* EN-220 (marine alga)	Indole alkaloids	**(89)** Tardioxopiperazine A—C_25_H_33_N_3_O_2_/408.264554 [M + H]^+^	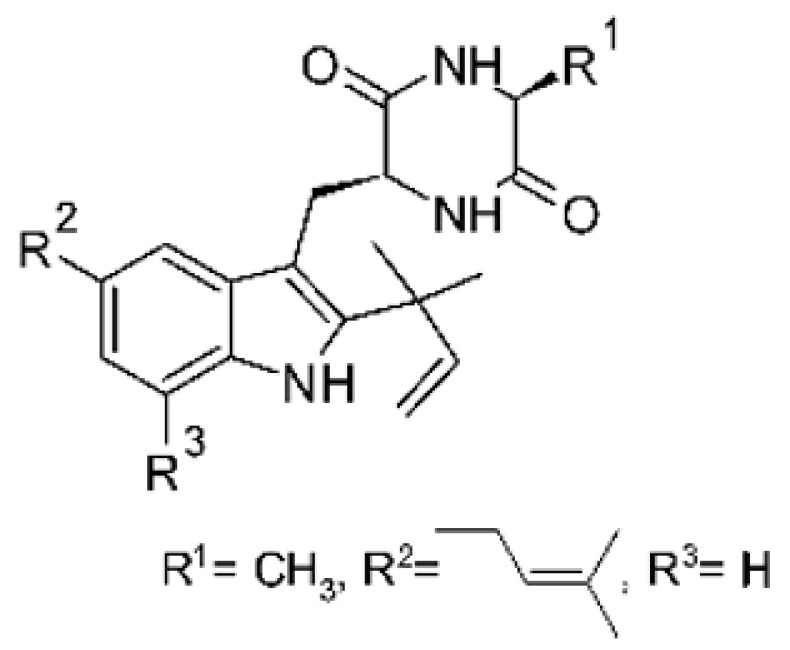	*E. coli*	64 µg/mL	[78]
*Drechmeria* sp. SYPF 8335 (roots)	Indole diterpenoids	**(90)** Drechmerin A—C_28_H_39_NO_3_/438.2998 [M + H]^+^	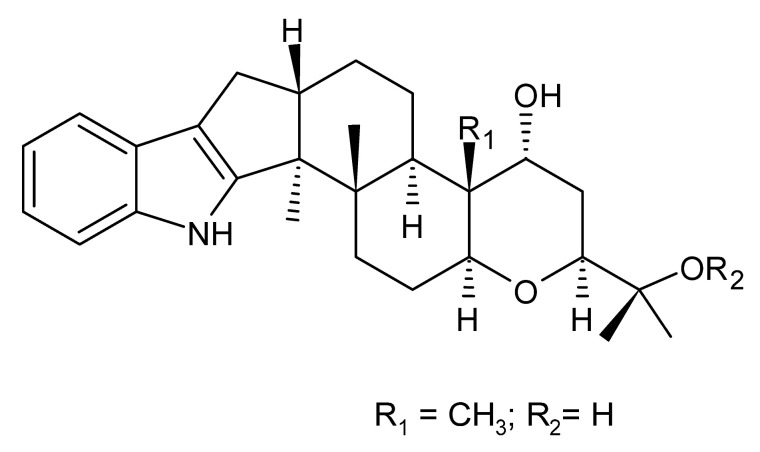	*P. aeruginosa* *K. pneumoniae*	>200 µg/mL >200 µg/mL	[79]
*Drechmeria* sp. SYPF 8335 (roots)	Indole diterpenoids	**(91)** Drechmerin B—C_28_H_37_NO_5_/490.2561 [M+Na]^+^	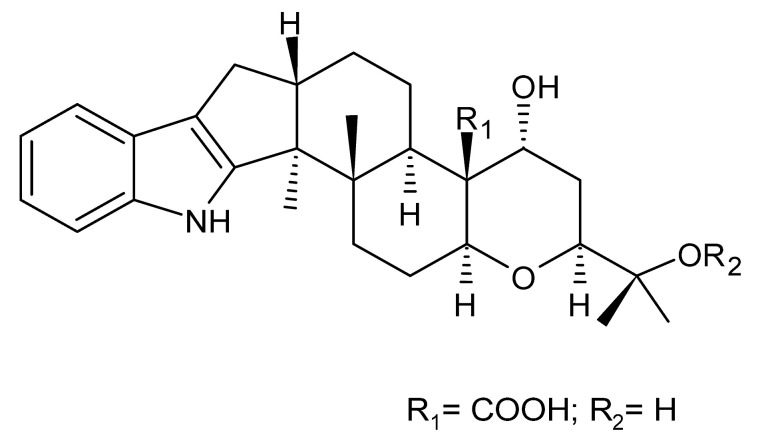	*P. aeruginosa* *K. pneumoniae*	>200 µg/mL >200 µg/mL	[79]
*Drechmeria* sp. SYPF 8335 (roots)	Indole diterpenoids	**(92)** Drechmerin C—C_28_H_37_NO_5_/558.3196 [M+Na]^+^	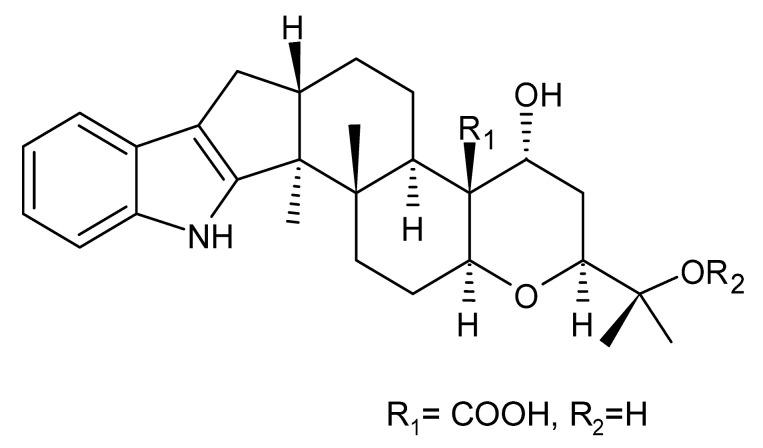	*P. aeruginosa* *K. pneumoniae*	>200 µg/mL >200 µg/mL	[79]
*Drechmeria* sp. SYPF 8335 (roots)	Indole diterpenoids	**(93)** Drechmerin D—C_33_H_45_NO_7_/554.3115 [M + H]^+^	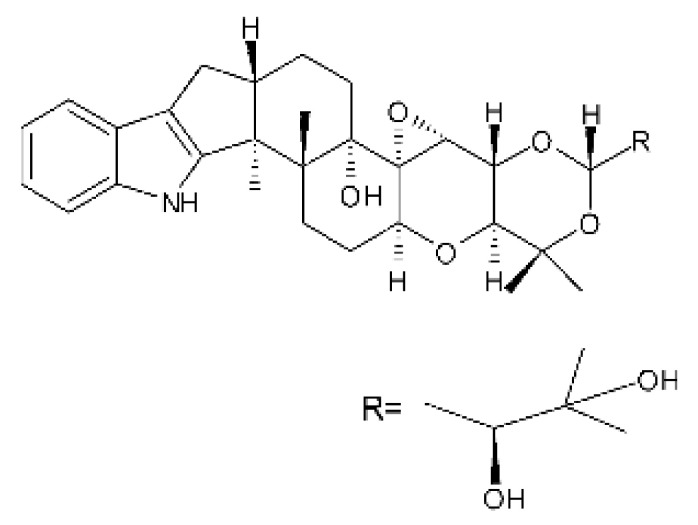	*P. aeruginosa* *K. pneumoniae*	>200 µg/mL >200 µg/mL	[79]
*Drechmeria* sp. SYPF 8335 (roots)	Indole diterpenoids	**(94)** Drechmerin E—C_33_H_45_NO_7_/554.3105 [M + H]^+^	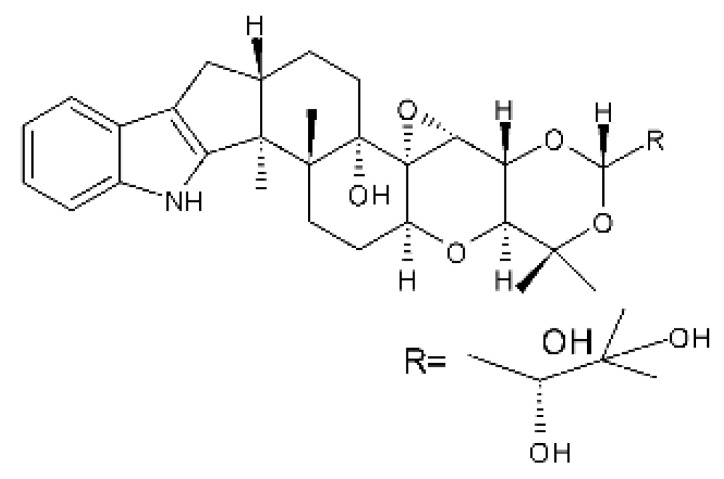	*P. aeruginosa* *K. pneumoniae*	>200µg/mL >200 µg/mL	[79]
*Drechmeria* sp. SYPF 8335 (roots)	Indole diterpenoids	**(95)** Drechmerin F—C_33_H_47_NO_7_/556.3276 [M + H]^+^	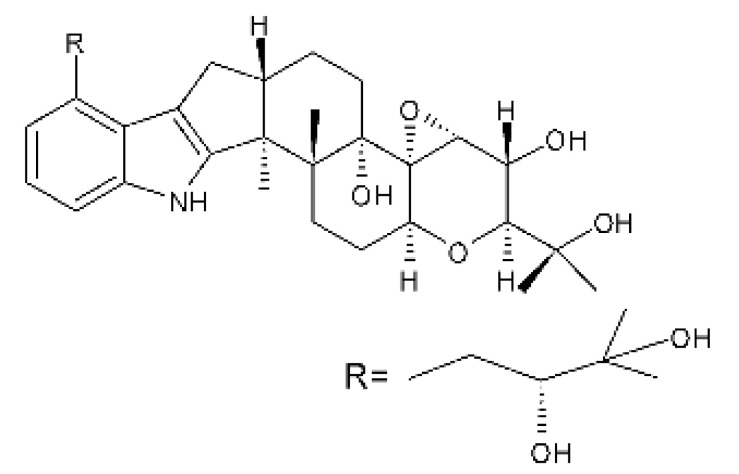	*P. aeruginosa* *K. pneumoniae*	>200 µg/mL >200µg/mL	[79]
*Drechmeria* sp. SYPF 8335 (roots)	Indole diterpenoids	**(96)** Drechmerin G—C_27_H_33_NO_5_/474.2250 [M+Na]^+^	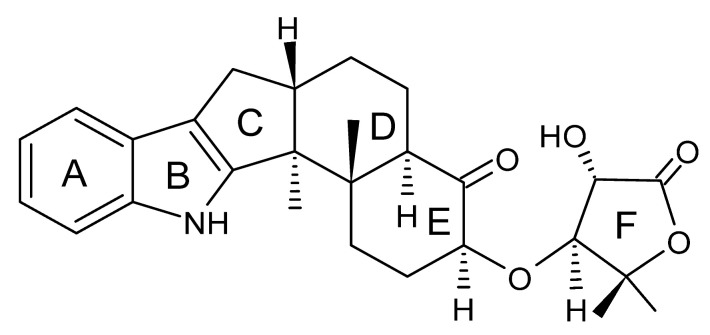	*P. aeruginosa* *K. pneumoniae*	>200 µg/mL >200 µg/mL	[79]
*Drechmeria* sp. SYPF 8335 (roots)	Indole diterpenoids	**(97)** terpendoles A—C_33_H_43_NO_6_/550.316315 [M + H]^+^	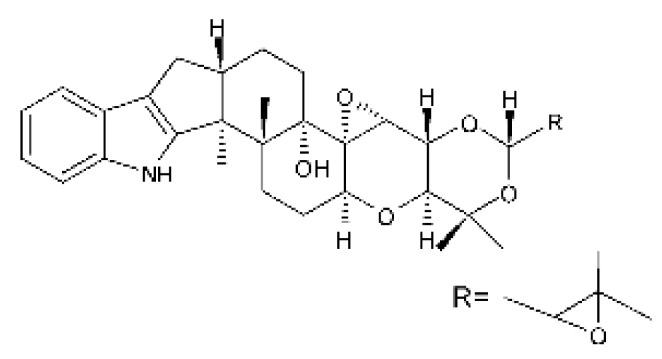	*P. aeruginosa* *K. pneumoniae*	>200 µg/mL >200 µg/mL	[79]
*Drechmeria* sp. SYPF 8335 (roots)	Indole diterpenoids	**(98)** terpendoles C—C_32_H_41_NO_5_/520.30575 [M + H]^+^	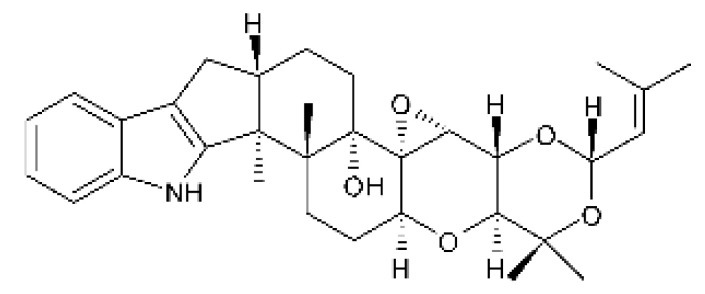	*P. aeruginosa* *K. pneumoniae*	>200 μg/mL >200 μg/mL	[79]
*Drechmeria* sp. SYPF 8335 (roots)	Indole diterpenoids	**(99)** terpendoles I—C_27_H_35_NO_5_/454.2588 [M + H]^+^	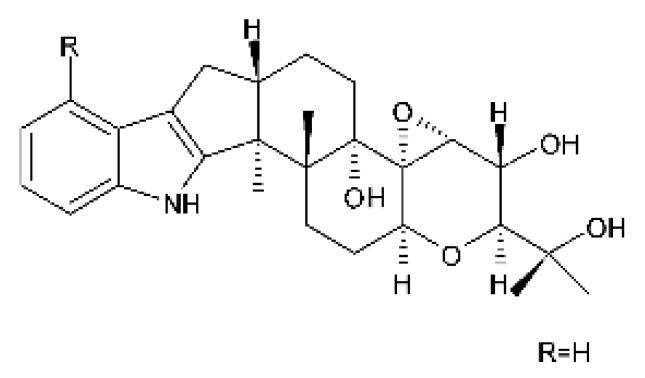	*P. aeruginosa* *K. pneumoniae*	>200 μg/mL >200 μg/mL	[79]
*Drechmeria* sp. SYPF 8335 (roots)	Indole diterpenoids	**(100)** dehydroxypaxilline—C_27_H_33_NO_3_/420.25332 [M + H]^+^	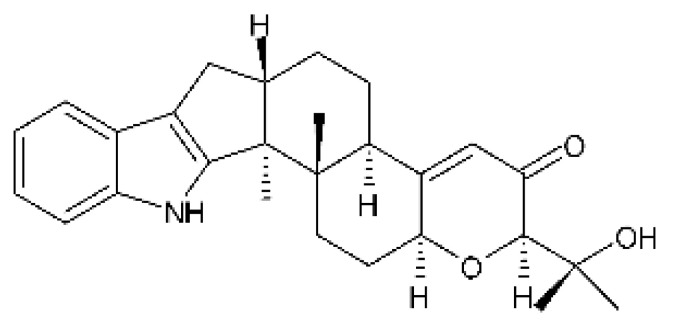	*P. aeruginosa* *K. pneumoniae*	>200 μg/mL >200 μg/mL	[79]
*Diaporthe terebinthifolii* LGMF907 (leaves)	Isocoumarin	**(101)** Diaporthin—C_13_H_14_O_5_/251.0914 [M + H]^+^	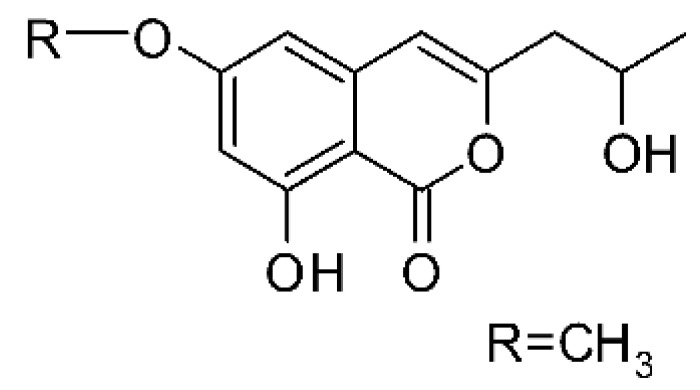	*E. coli* NRRL B-3708	1.73 mm (100 μg/dis)	[80]
*Diaporthe terebinthifolii* LGMF907 (leaves)	Isocoumarin	**(102)** Orthosporin—C_12_H_12_O_5_/237.07575 [M + H]^+^	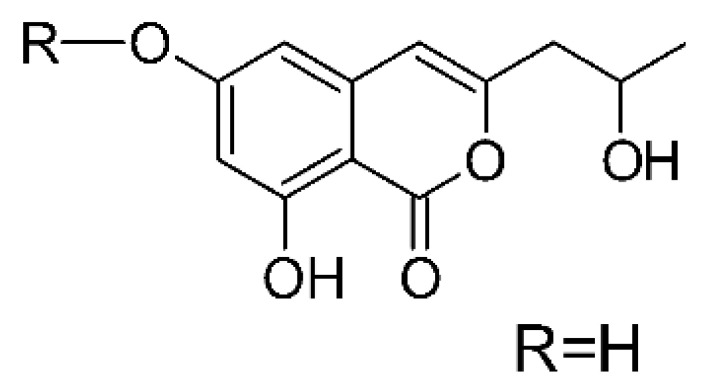	*E. coli* NRRL B-3708	1.03 mm (100 μg/dis)	[80]
*Xylaria* sp. GDG-102 (leaves)	Isocoumarin	**(103)** (-)-5-carboxylmellein—C_11_H_10_O_5_/223.0601 [M + H]^+^	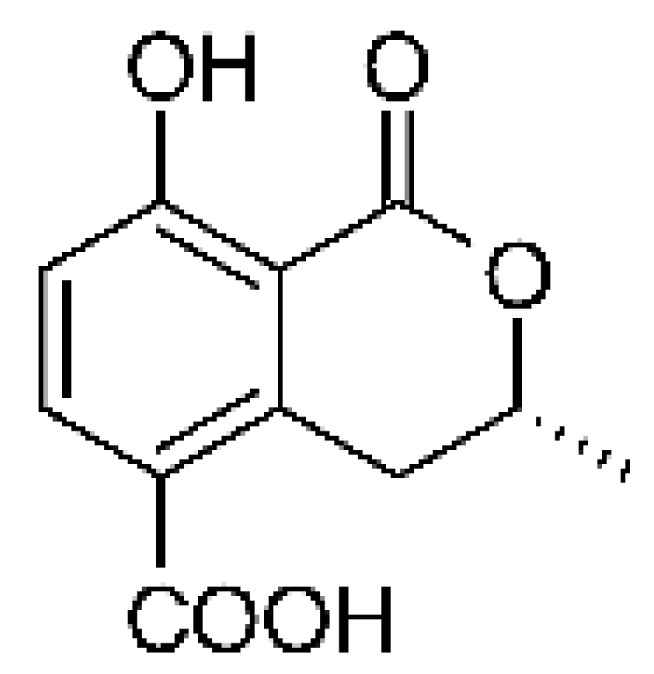	*E. coli*	25 μg/mL	[81]
*Xylaria* sp. GDG-102 (leaves)	Isocoumarin	**(104)** (-)-5-methylmellein—C_11_H_12_O_3_/193.085921 [M + H]^+^	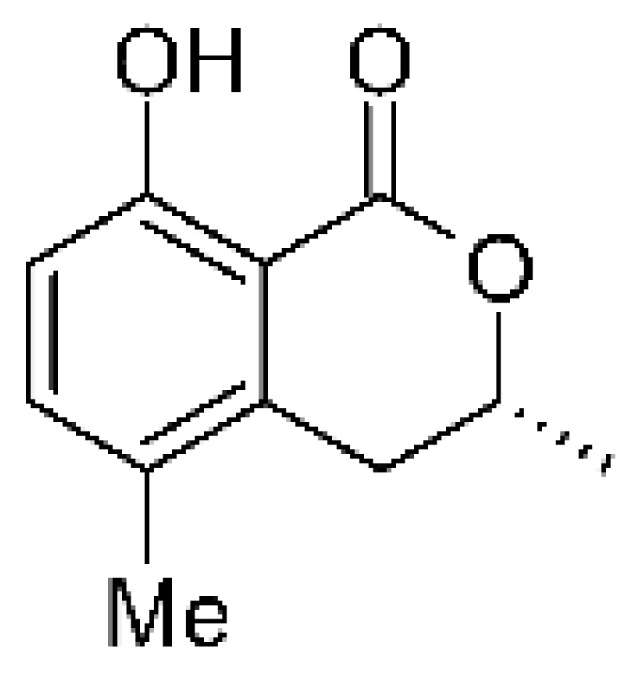	*E. coli*	12.5 μg/mL	[81]
*Exserohilum rostratum* ER1.1	Isocoumarin derivative	**(105)** monocerin—C_14_H_18_O_8/_ 309 [M + H]^+^	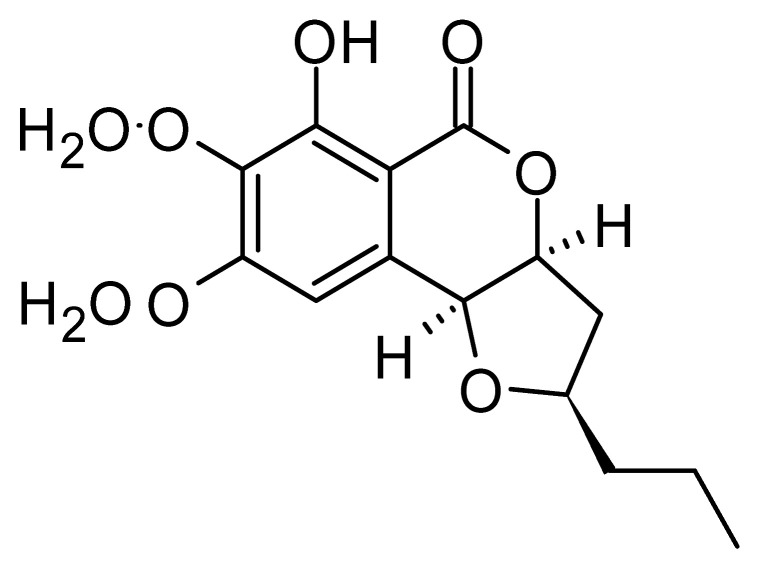	*E.coli* ATCC 25922 *P. aeruginosa* ATCC 27853 *S. typhimurium* ATCC 14028	15.62 μg/mL 15.62 μg/mL 31.25 μg/mL	[82]
*F. proliferatum* AF-04 (onion)	1,4—naphthoquinones	**(106)** 5-O-methylsolaniol—C_16_H_18_O_6_/307.117615 [M + H]^+^	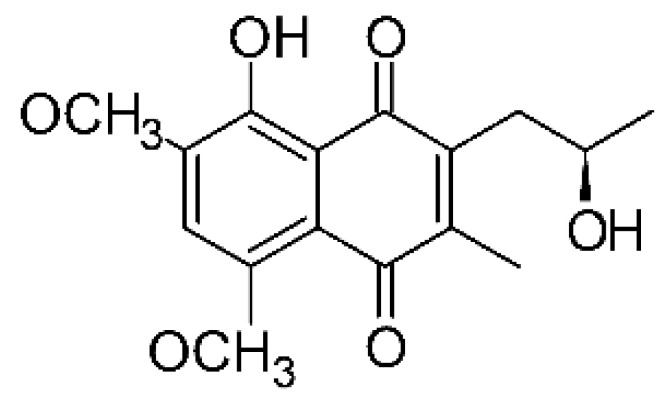	*E. coli*	25 μg/mL	[55]
*F. proliferatum* AF-04 (onion)	1,4—naphthoquinones	**(107)** 5-O-methyljavanicin—C_16_H_16_O_6_/305.101965 [M + H]^+^	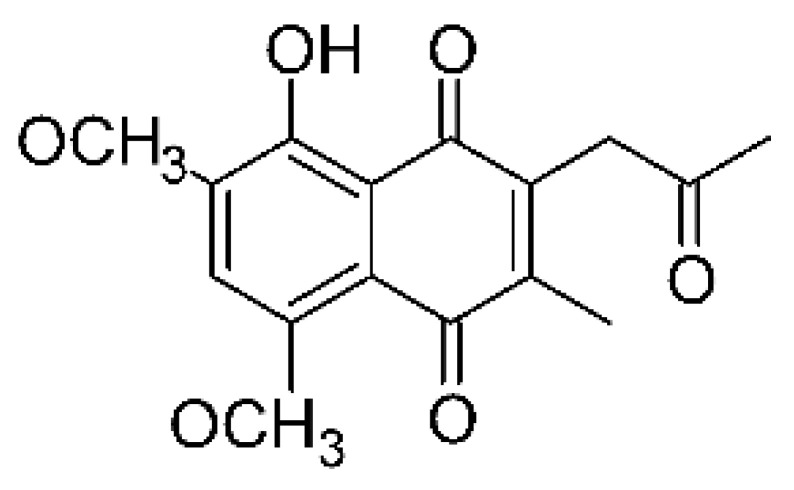	*E. coli*	25 μg/mL	[55]
*F. proliferatum* AF-04 (onion)	1,4—naphthoquinones	**(108)** methyl ether fusarubin—C_17_H_18_O_7_/335.112529 [M + H]^+^	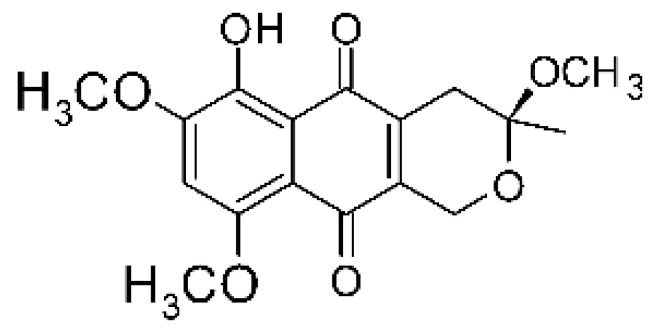	*E. coli*	50 μg/mL	[55]
*F. proliferatum* AF-04 (onion)	1,4—naphthoquinones	**(109)** anhydrojavanicin—C_15_H_14_O_5_/275.0914 [M + H]^+^	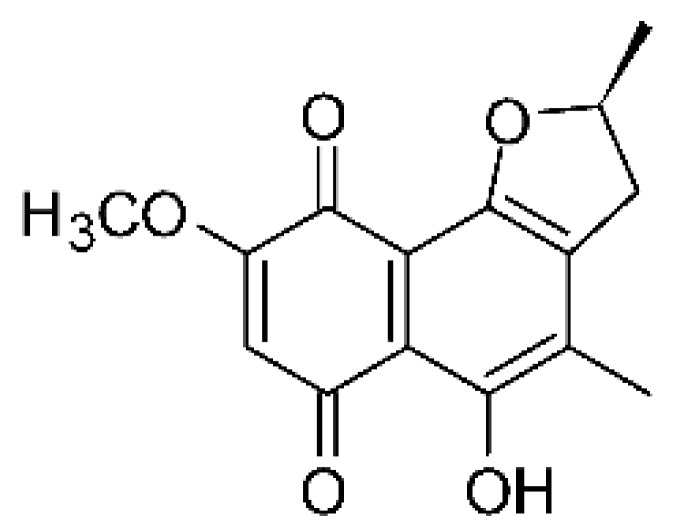	*E. coli*	25 μg/mL	[55]
*Aspergillus* sp. A-WG-1	Phenalenone derivatives	**(110)** Aspergillussanone C—C_35_H_44_O_10_/647.2826 [M+Na]^+^	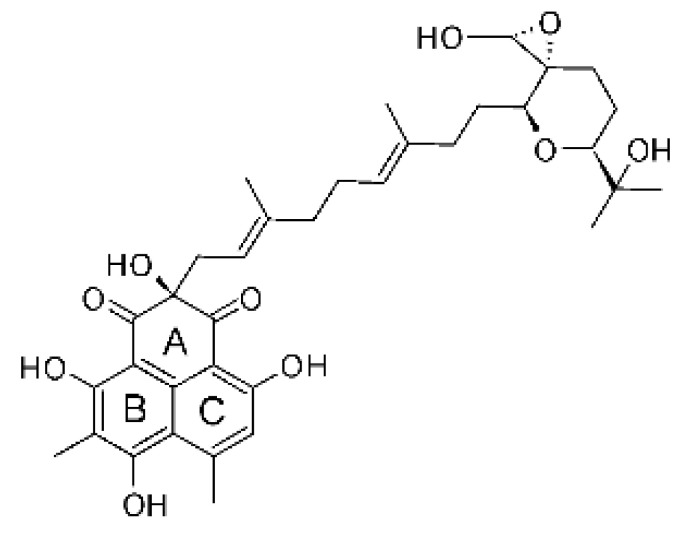	*E. coli* ATCC 25922 *P. aeruginosa* ATCC 9027	> 50.0 µg/mL > 50.0 µg/mL	[83]
*Aspergillus* sp. A-WG-1	Phenalenone derivatives	**(111)** Aspergillussanone D—615.2925 [M+Na]^+^	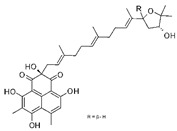	*E. coli* ATCC 25922 *P. aeruginosa* ATCC 9027	> 50.0 µg/mL 38.47 µg/mL	[83]
*Aspergillus* sp. A-WG-1	Phenalenone derivatives	**(112)** Aspergillussanone E—615.2926 [M+Na]^+^	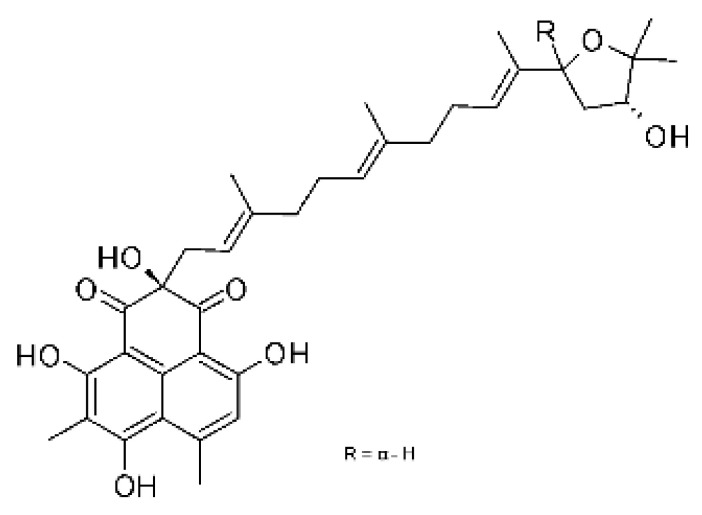	*E. coli* ATCC 25922 *P. aeruginosa* ATCC 9027	7.83 µg/mL > 50.0 µg/mL	[83]
*Aspergillus* sp. A-WG-1	Phenalenone derivatives	**(113)** Aspergillussanone F—C_35_H_46_O_8_/617.3079 [M+Na]^+^	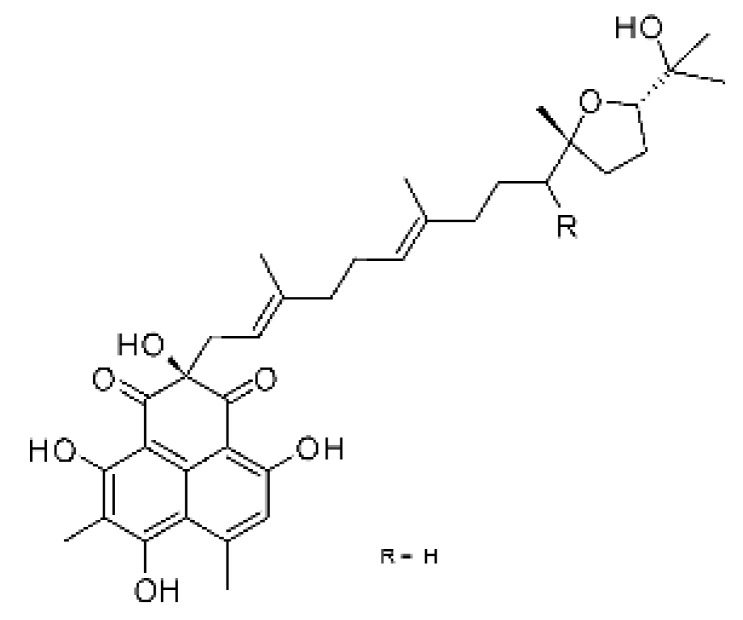	*E. coli* ATCC 25922 *P. aeruginosa* ATCC 9027	3.93 µg/mL 26.56 µg/mL	[83]
*Aspergillus* sp. A-WG-1	Phenalenone derivatives	**(114)** Aspergillussanone G—633.3031 [M+Na]^+^	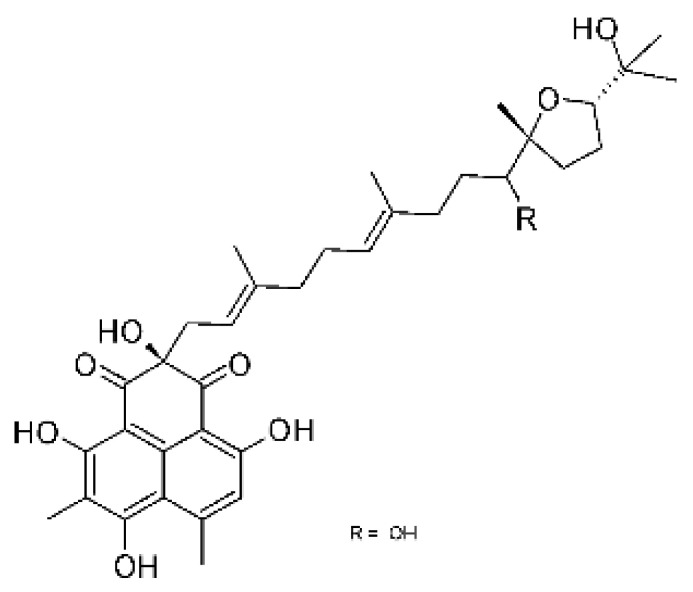	*E. coli* ATCC 25922 *P. aeruginosa* ATCC 9027	>50.0 µg/mL 24.46 µg/mL	[83]
*Aspergillus* sp. A-WG-1	Phenalenone derivatives	**(115)** Aspergillussanone H—C_35_H_42_O_9_/629.2720 [M+Na]^+^	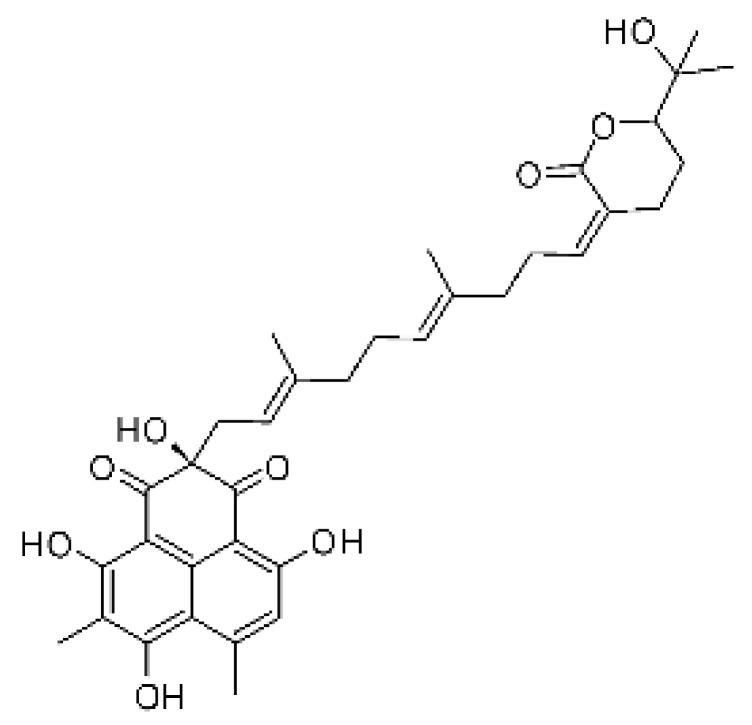	*E. coli* ATCC 25922 *P. aeruginosa* ATCC 9027	5.87 µg/mL 8.59 µg/mL	[83]
*Aspergillus* sp. A-WG-1	Phenalenone derivatives	**(116)** Aspergillussanone I—C_38_H_50_O_8_/657.3395 [M+Na]^+^	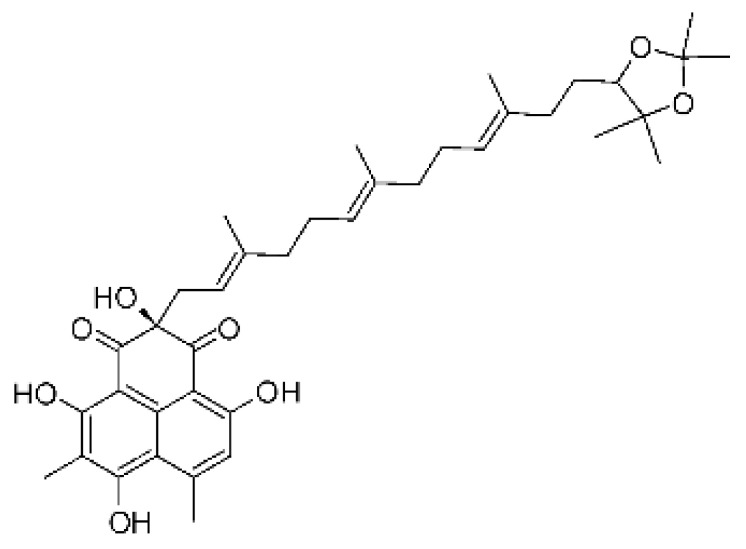	*E. coli* ATCC 25922 *P. aeruginosa* ATCC 9027	>50.0 µg/mL 12.0 µg/mL	[83]
*Aspergillus* sp. A-WG-1	phenalenone derivatives	**(117)** Aspergillussanones J—689.3295 [M+Na]^+^	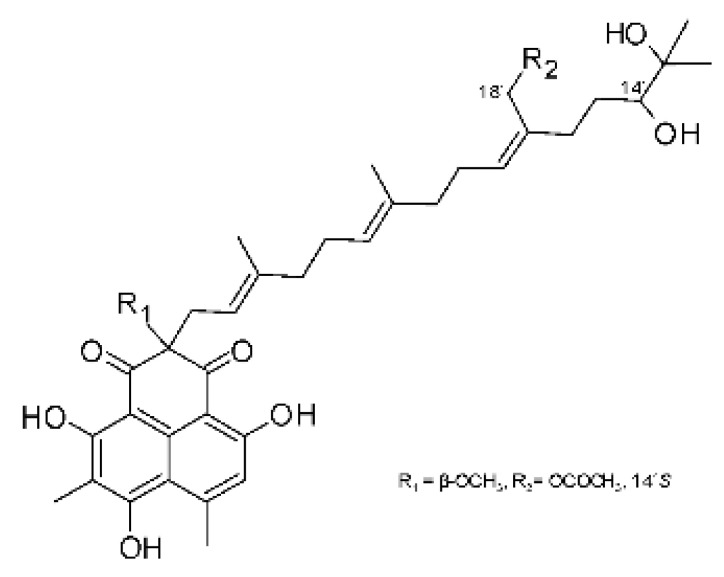	*E. coli* ATCC 25922 *P. aeruginosa* ATCC 9027	5.34 µg/mL 28.50 µg/mL	[83]
*Aspergillus* sp. A-WG-1	Phenalenone derivatives	**(118)** Aspergillussanones K—675.3139 [M+Na]^+^	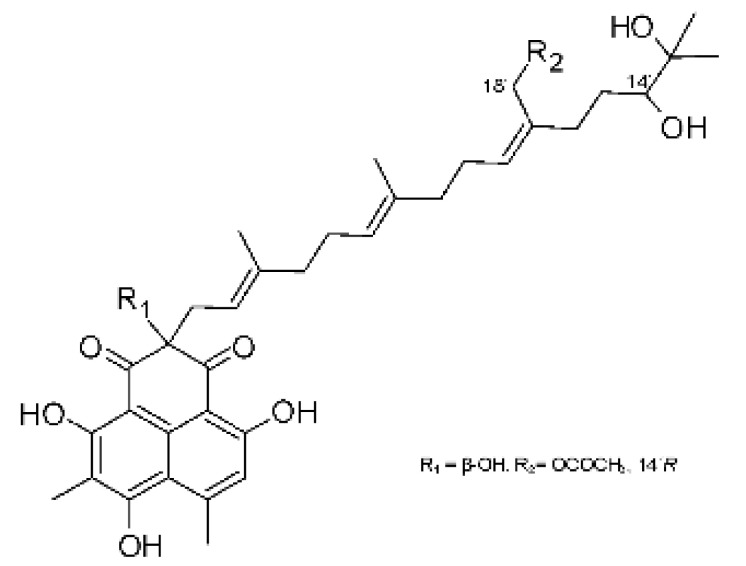	*E. coli* ATCC 25922 *P. aeruginosa* ATCC 9027	>50.0 µg/mL 6.55 µg/mL	[83]
*Aspergillus* sp. A-WG-1	Phenalenone derivatives	**(119)** Aspergillussanones L—617.3083 [M+Na]^+^	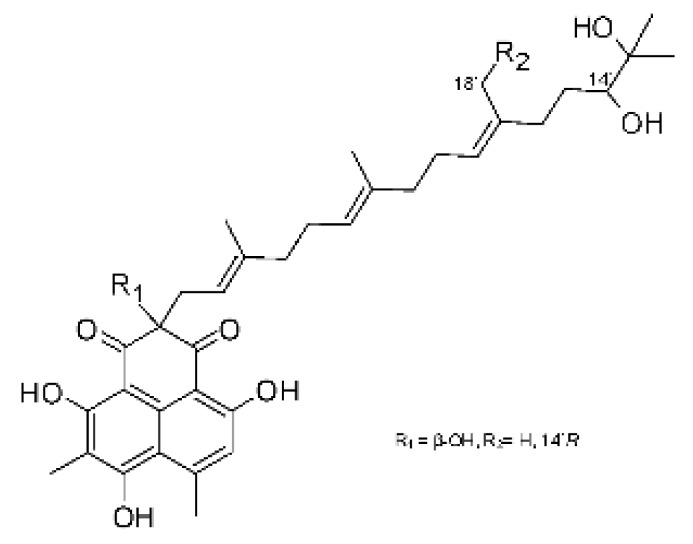	*E. coli* ATCC25922 *P. aeruginosa* ATCC 9027	>50.0 µg/mL 1.87 µg/mL	[83]
*Aspergillus* sp. A-WG-1	Phenalenone derivatives	**(120)** 11—593.727 [M+Na]^+^	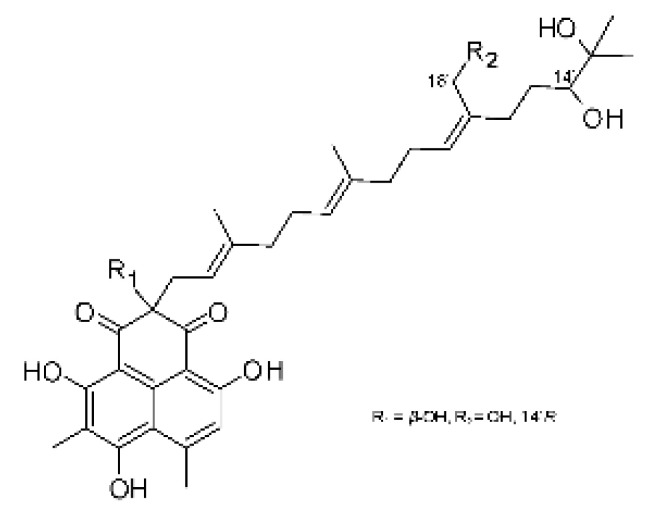	*E. coli* ATCC 25922 *P. aeruginosa* ATCC 9027	1.88 µg/mL 19.07 µg/mL	[83]
*Pestalotiopsis mangiferae* (leaves)	Phenolic compound	**(121)** 4-(2,4,7-trioxa-bicyclo [4.1.0]heptan-3-yl) phenol—C_10_H_10_O_4_/195.0654 [M + H]^+^	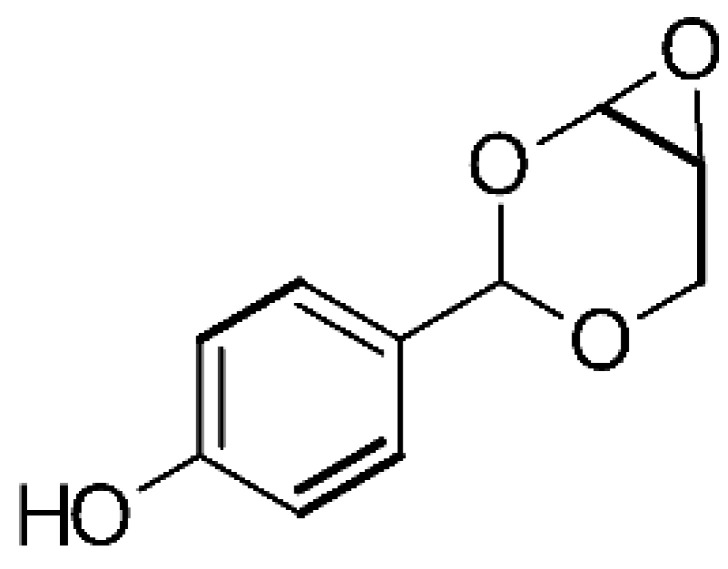	*Escherichia coli* MTCC 443 *Klebsiella pneumoniae* MTCC 109 *Pseudomonas aeruginosa* MTCC 424	1.25 μg/mL 0.039 μg/mL 5.0 μg/mL	[84]
*Stemphylium* sp. 33231 (leaves)	Phenolic sulfate derivatives	**(122)** Stemphol A—C_15_H_23_NaO_5_S/361.1066 [M+Na]^+^	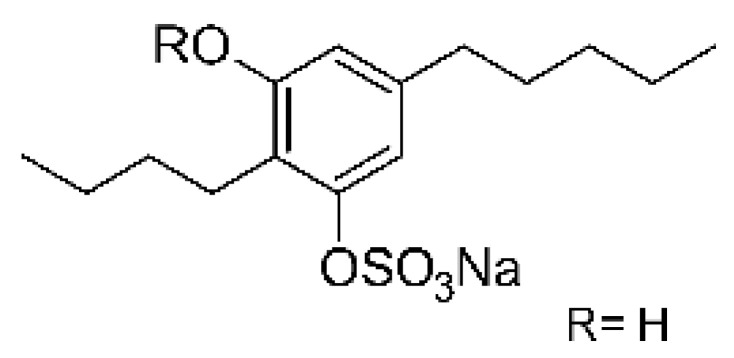	*Escherichia coli* ATCC 25922	5.0 μg/mL	[85]
*Stemphylium* sp. 33231 (leaves)	Phenolic sulfate derivatives	**(123)** Stemphol B—C_17_H_25_NaO_5_S/403.1165 [M+Na]^+^	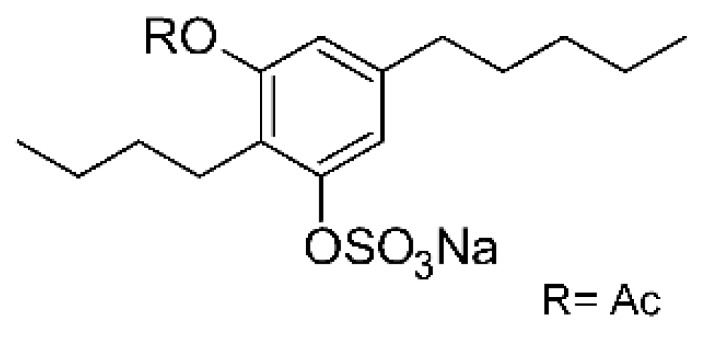	*E. coli* ATCC 25922	0.6 μg/mL	[85]
*Stemphylium* sp. 33231 (leaves)	Phenolic sulfate derivatives	**(124)** Sstemphol—C_15_H_24_O_2_/237.184906 [M + H]^+^	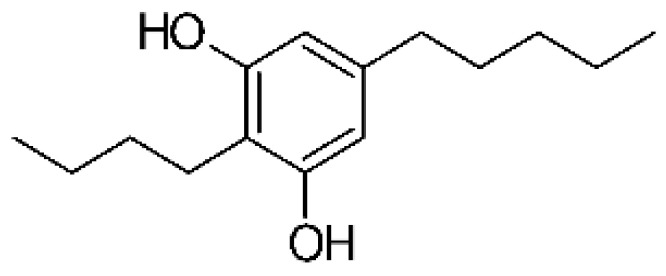	*E. coli* ATCC 25922	0.6 μg/mL	[85]
*Diaporthe* sp. F2934 (leaves)	Phomosine derivatives	**(125)** Phomosine A—C_18_H_20_O_7_/349.128179 [M + H]^+^	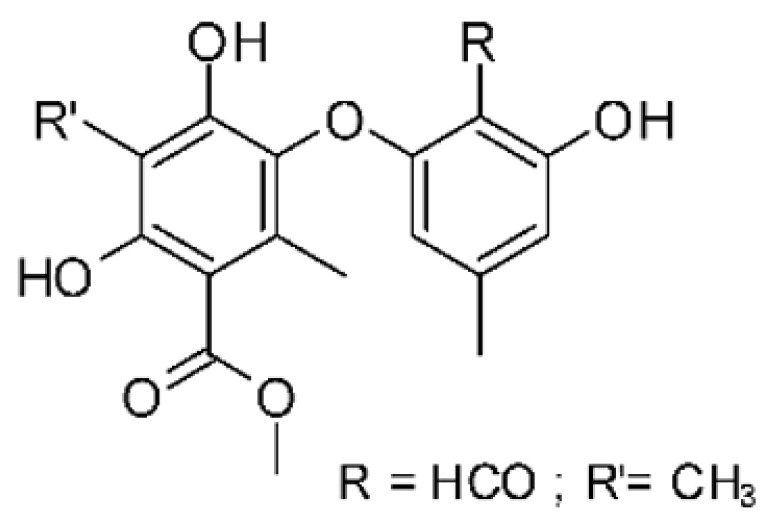	*Enterococcus cloacae* ATCC 13047	11 mm	[86]
*Diaporthe* sp. F2934 (leaves)	Phomosine derivatives	**(126)** Phomosine C—C_17_H_18_O_7_/335.112529 [M + H]^+^	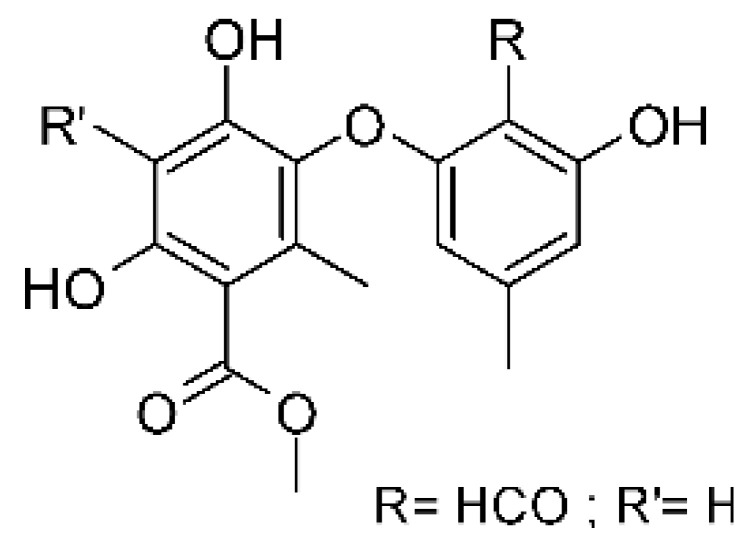	*E. cloacae* ATCC 13047	8 mm	[86]
*Diaporthe phaseolorum* (8S) (seed)	Phthalate	**(127)** Di-(2-ethylhexyl) phthalate (DEHP)—C_24_H_38_O_4_/391.284286 [M + H]^+^	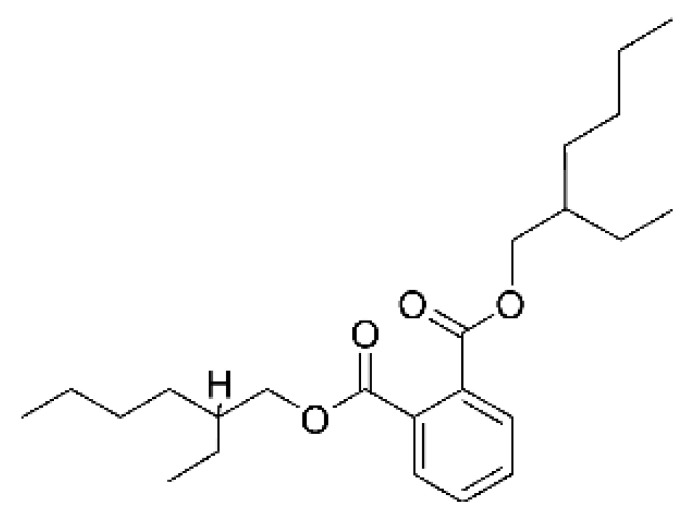	*E. coli* Multiresistant Strain *E. coli* Susceptible strain *P. aeruginosa* Multiresistant strain *P. aeruginosa* Susceptible strain	>30 µg/mL >30 µg/mL 0.23 µg/mL 0.23 µg/mL	[54]
*Xylaria* sp. GDG102 (leaves)	Phthalide derivative	**(128)** Xylarphthalide A—C_11_H_10_O_6/_237.0401[M−H]^−^	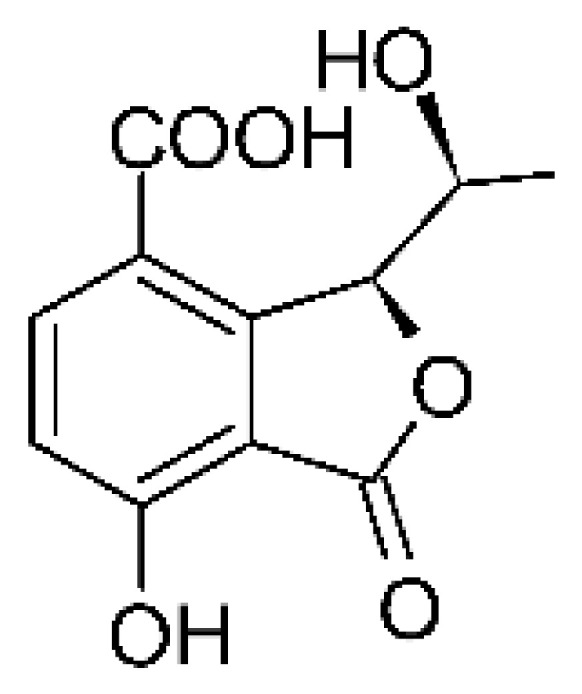	*E. coli*	12.5 μg/mL	[81]
*Epicoccum nigrum* SCNU-F0002 (fruit)	Polyketide—benzofuranone derivatives	**(129)** 1-(4-hydroxy-2-methoxybenzofuran-5-yl) butan-1-one—C_13_H_14_O_4_/233 [M-H]^−^	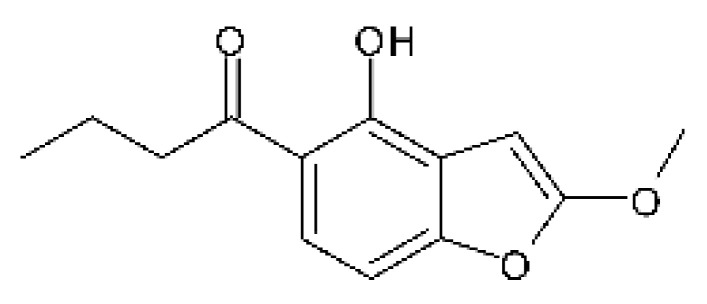	*Escherichia. coli* ATCC 8739 *P. aeruginosa* ATCC 9027	50 µg/mL >100 μg/mL	[87]
*Colletotrichum* sp. BS4	Polyketides	**(130)** Colletotrichone A—C_18_H_20_O_7/_349.1280, [M + H]^+^	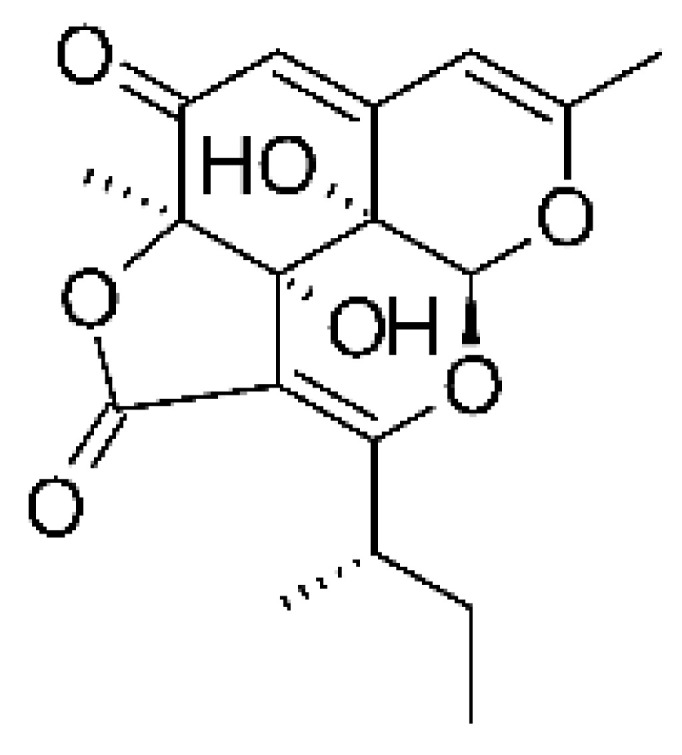	*Escherichia coli* DSM 1116 *Pseudomonas aeruginosa* DSM 22644	1.0 μg/mL >10 μg/mL	[88]
*Colletotrichum* sp. BS4	Polyketides	**(131)** Colletotrichone B—C_18_H_20_O_5_/317.1385, [M + H]^+^	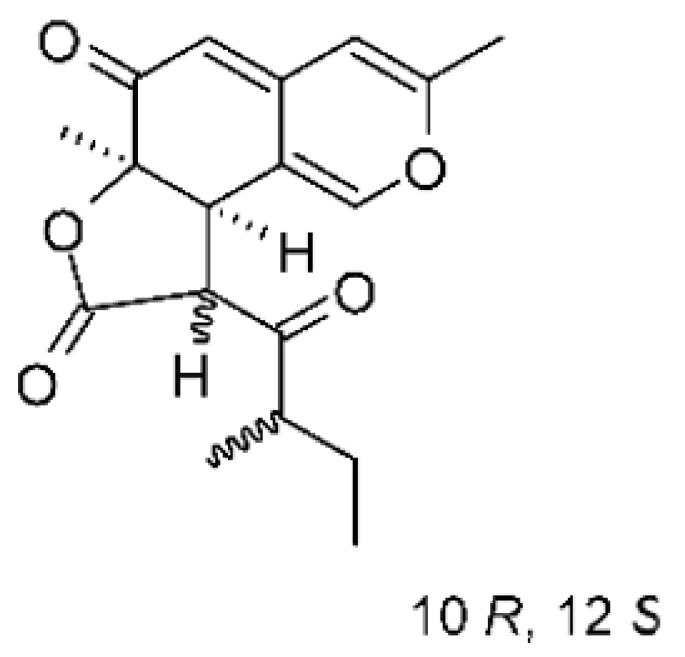	*E. coli* DSM 1116 *P. aeruginosa* DSM 22644	>10 μg/mL >10 μg/mL	[88]
*Colletotrichum* sp. BS4	Polyketides	**(132)** Colletotrichone C—C_18_H_22_O_5_/319.1542, [M + H]^+^	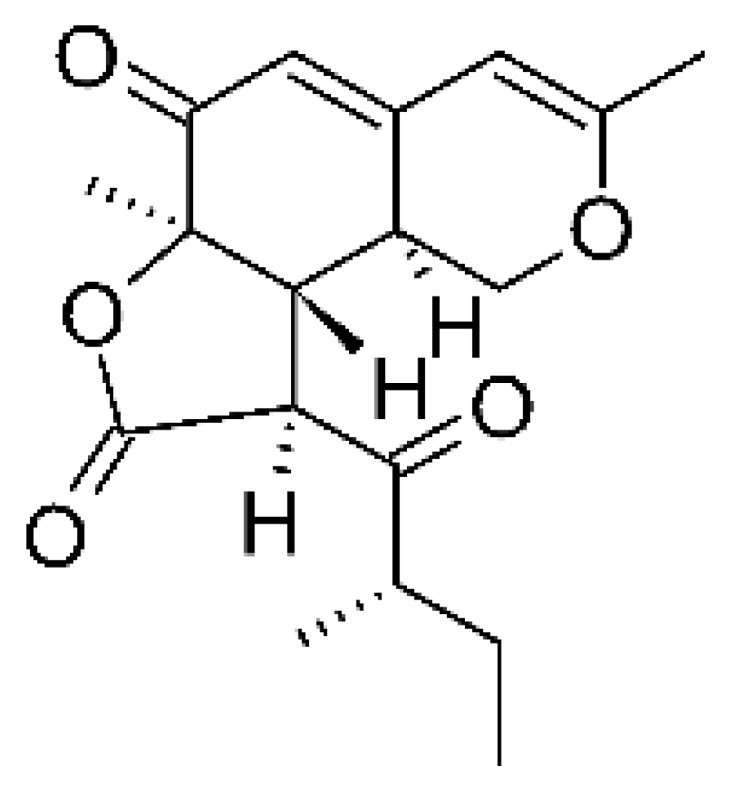	*E. coli* DSM 1116 *P. aeruginosa* DSM 22644	5.0 μg/mL >10 μg/mL	[88]
*Colletotrichum* sp. BS4	Polyketides	**(133)** chermesinone B—C_18_H_20_O_5_/317.13835 [M + H]^+^	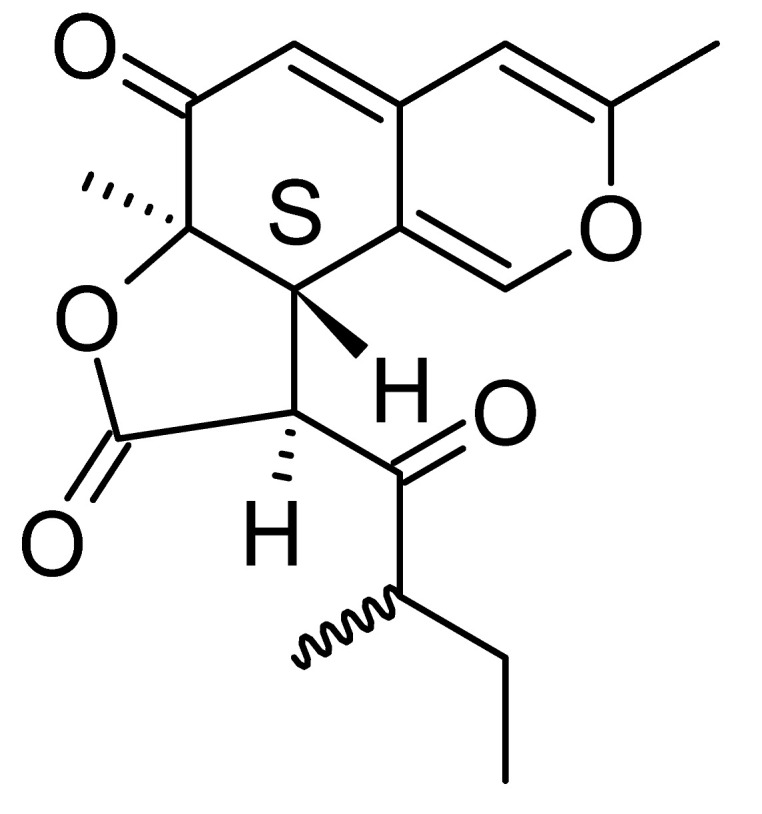	*E. coli* DSM 1116 *P. aeruginosa* DSM 22644	>10 μg/mL >10 μg/mL	[88]
*Penicillium* sp. CAMMC64 (leaves)	Polyketides	**(134)** Penialidin A—C_14_H_12_O_8_/309.06064 [M + H]^+^	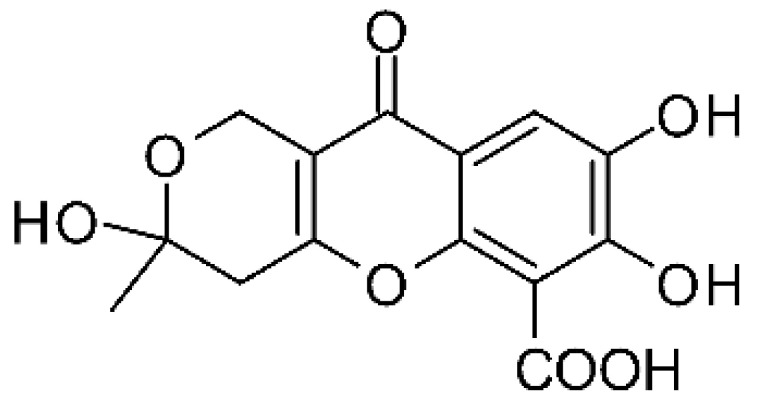	*Acinetobacter sp*. BD4 DSM 586 *E. coli* DSM 1116 *Escherichia coli* DSM 682	>10 μg/mL >10 μg/mL >10 μg/mL	[89]
*Penicillium* sp. CAMMC64 (leaves)	Polyketides	**(135)** Penialidin B—C_15_H_14_O_8_/323.07635 [M + H]^+^	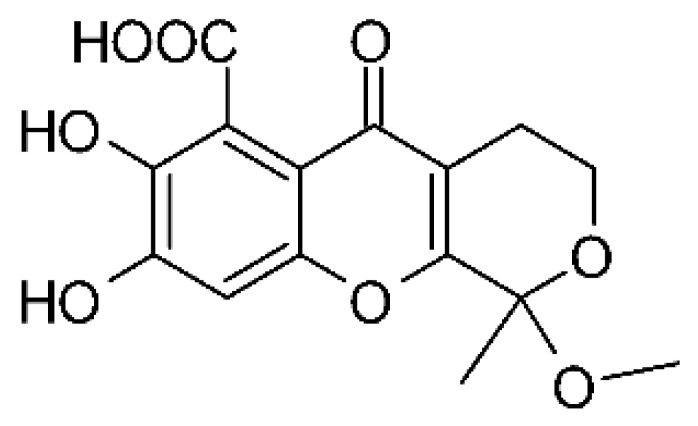	*Acinetobacter* sp. BD4 DSM 586 *Escherichia coli* DSM 1116 *E. coli* DSM 682	>10 μg/mL 10 μg/mL 10 μg/mL	[89]
*Penicillium* sp. CAMMC64 (leaves)	Polyketides	**(136)** Penialidin C—C_14_H_10_O_7_/291.04996 [M + H]^+^	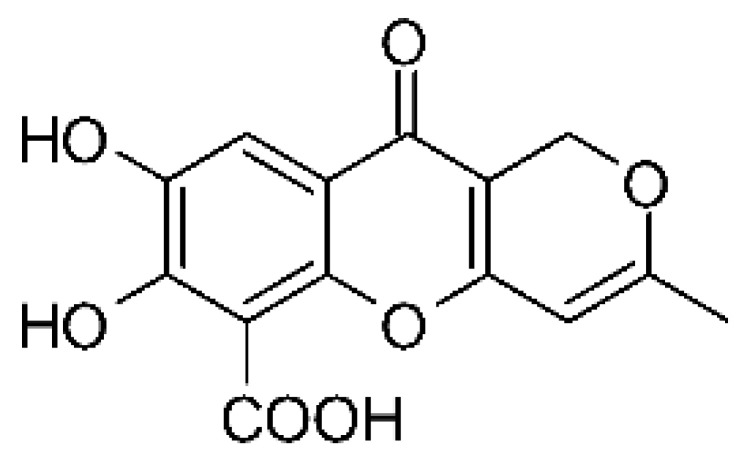	*Acinetobacter* sp. BD4 DSM 586 *Escherichia coli* DSM 1116 *E. coli* DSM 682	>10 μg/mL 10 μg/mL 10 μg/mL	[89]
*Fusarium solani* DO7	polysaccharides	**(137)** DY1	(1 →)-α-D-Glcp, (1 → 3)-β-L-Rhaf, (1 → 4)-β-D-Xylp, (1 → 6)-α-D-Glcp, (1 → 2,6)-α-D-Glcp and (1 → 2)-β-D-Galp	*E. coli* *Salmonella*	20 μg/mL 25 μg/mL	[90]
*F. solani* DO7	Polysaccharides	**(138)** DY2	(1 →)-β-D-Glcp, (1 → 2)-α-L-Rhaf, (1 → 3)-α-L-Araf, (1 → 4)-β-D-Glcp, (1 → 4,6)-β-D-Glcp and (1 → 3)-α-D-Galp	*E. coli* *Salmonella*	15 μg/mL 20 μg/mL	[90]
*Epicoccum nigrum* SCNU-F0002 (fruit)	Pyrones	**(139)** radicinol derivative—C_12_H_16_O_5_/241.10705 [M + H]^+^	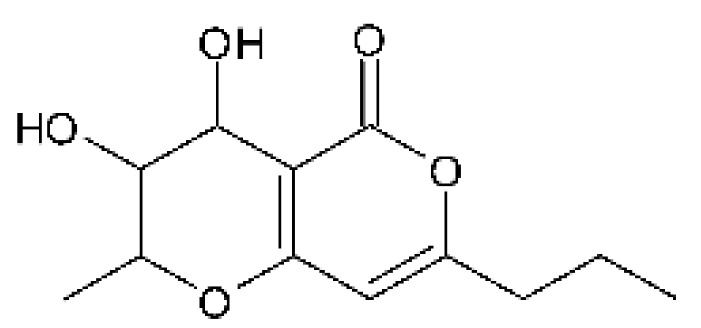	*E. coli* *P. aeruginosa*	>100 µg/mL >100 µg/mL	[87]
*Alternaria tenuissima* SP-07	Pyrones	**(140)** Solanapyrone P—C_16_H_22_O_3_/263.1635 [M + H]^+^	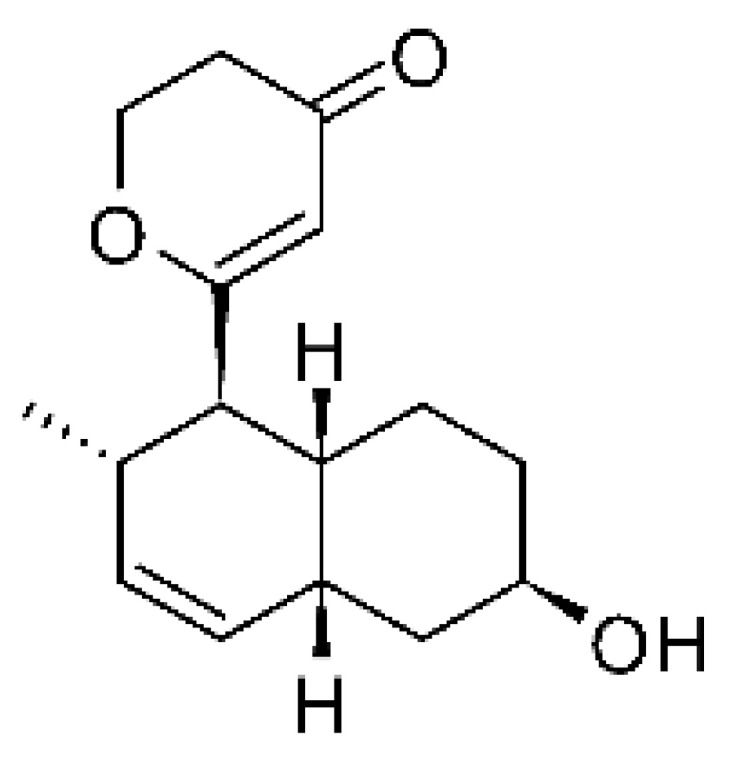	*E. coli*	100 μg/mL	[63]
*A. tenuissima* SP-07	Pyrones	**(141)** Solanapyrone Q—C_16_H_20_O_2_/245.1539 [M + H]^+^	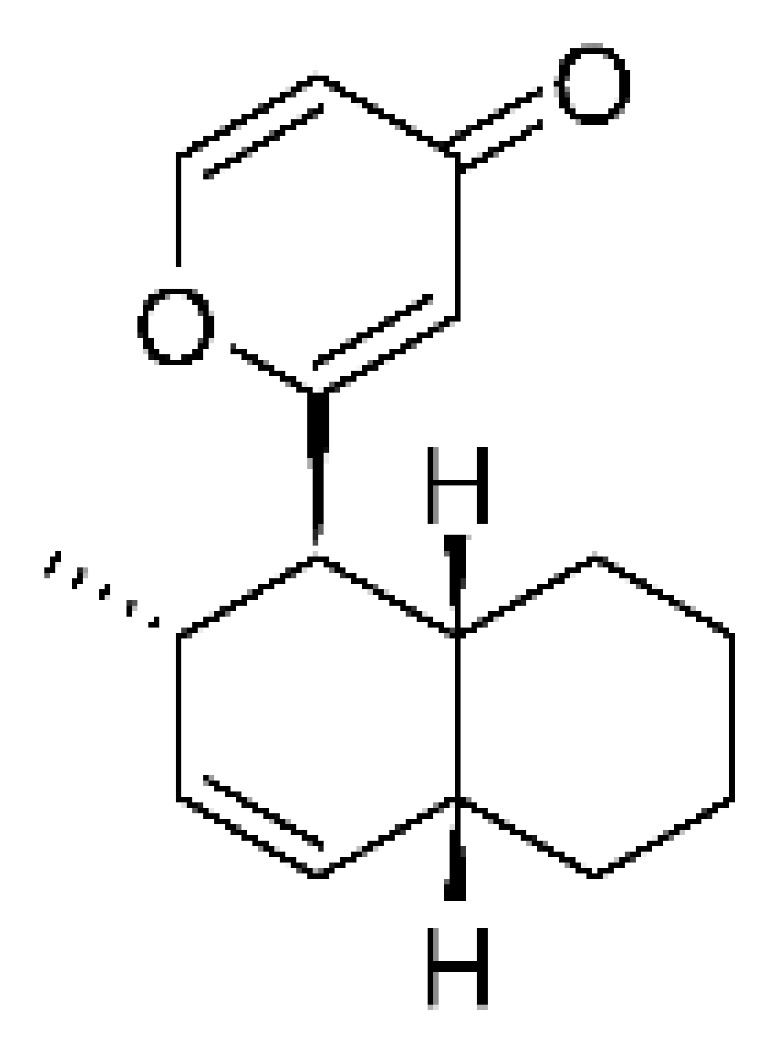	*E. coli*	100 μg/mL	[63]
*A. tenuissima* SP-07	Pyrones	**(142)** Solanapyrone R—C_19_H_26_O_4_/317.1742 [M + H]^+^	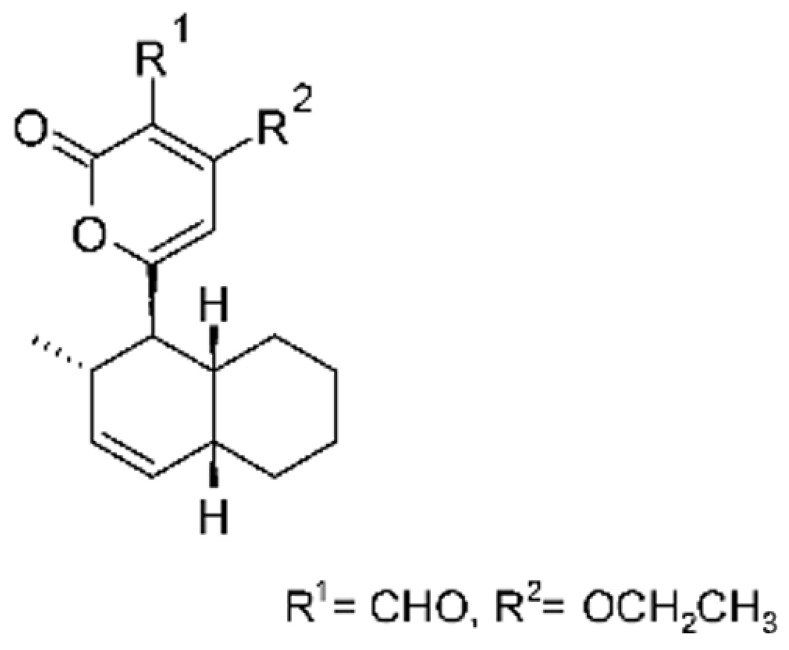	*E. coli*	>100 μg/mL	[63]
*A. tenuissima* SP-07	Pyrones	**(143)** solanapyrones A—C_18_H_24_O_4_/305.174736 [M + H]^+^	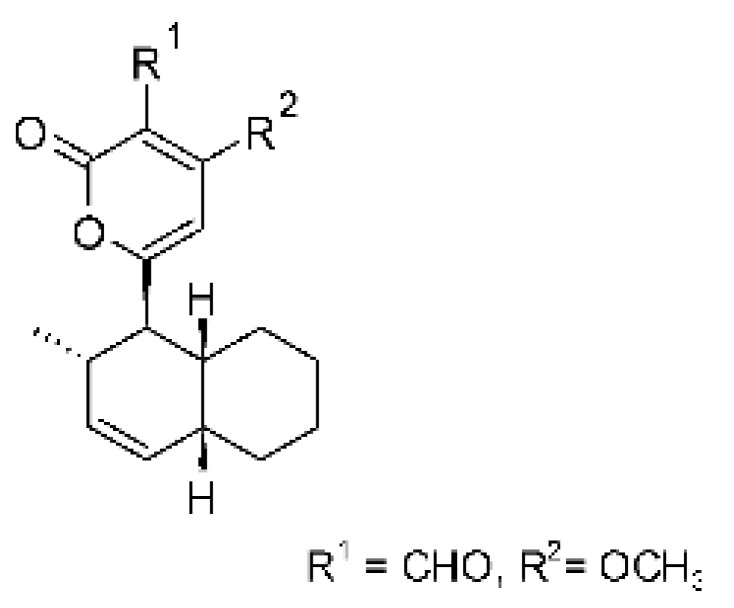	*E. coli*	100 μg/mL	[63]
*A. tenuissima* SP-07	Pyrones	**(144)** solanapyrones B—C_18_H_24_O_4_/305.174736 [M + H]^+^	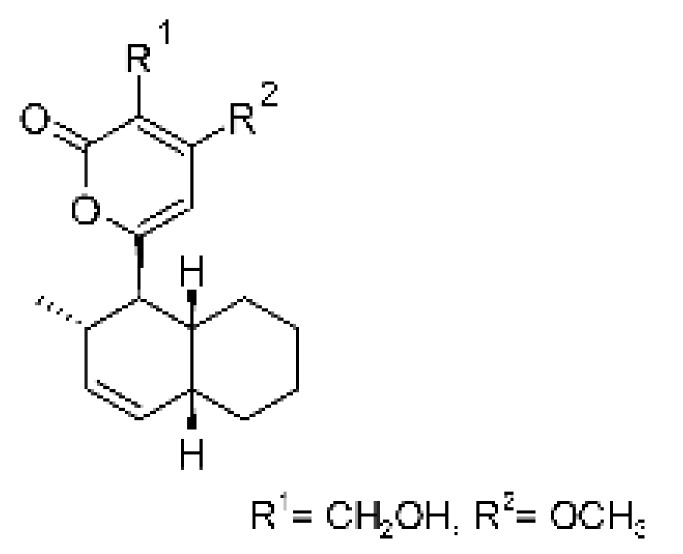	*E. coli*	>100 μg/mL	[63]
*A. tenuissima* SP-07	Pyrones	**(145)** solanapyrones C—C_19_H_27_NO_4_/334.201285 [M + H]^+^	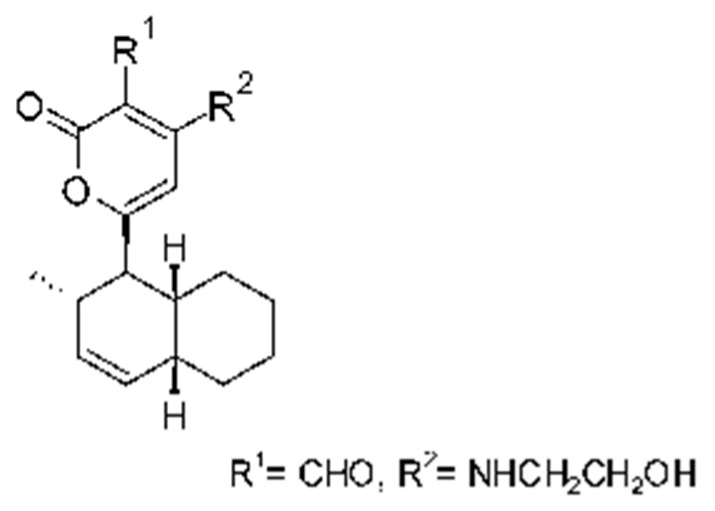	*E. coli*	100 μg/mL	[63]
*Leptosphaeria* sp. XL026 (leaves)	Sesquiterpenoids	**(146)** Leptosphin A—C_15_H_16_O_2_S/261.0943 [M + H]^+^	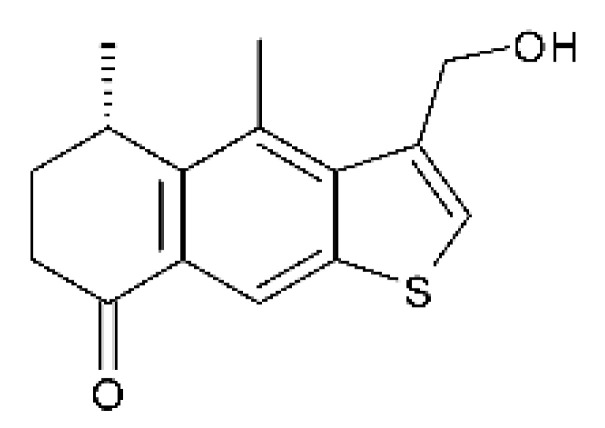	*E. coli* *P. aeruginosa* *S. typhimurium*	50 μg/mL 100 μg/mL 100 μg/mL	[67]
*Leptosphaeria* sp. XL026 (leaves)	Sesquiterpenoids	**(147)** Leptosphin B—C_15_H_20_O_4_/263.1277 [M−H]^−^	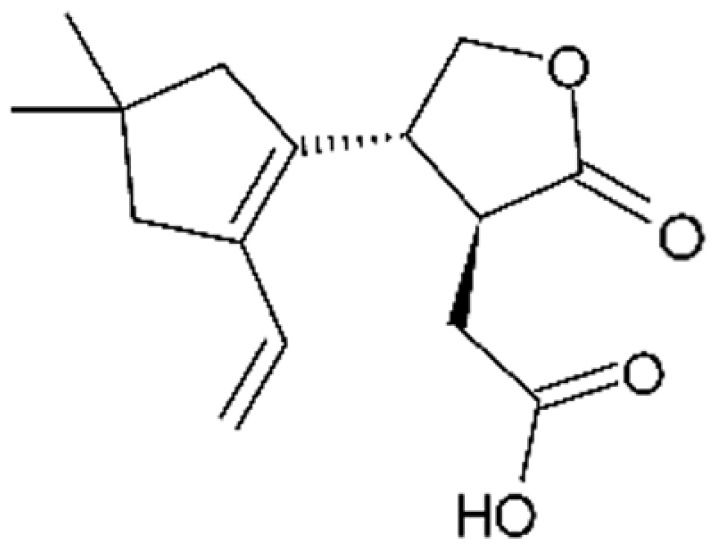	*E. coli* *P. aeruginosaSalmonella typhimurium*	50 μg/mL >100 μg/mL >100 μg/mL	[67]
*Fusarium avenaceum* SF-1502 (root) and *F. proliferatum* AF-04 (onion)	Sesquiterpenoids	**(148)** Epicyclonerodiol oxide—C_15_H_28_O_3/_257.211121 [M + H]^+^	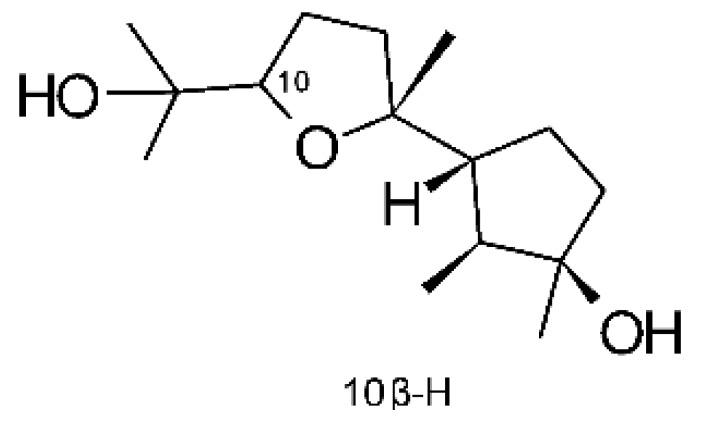	*E. coli*	>50 μg/mL	[55]
*F. avenaceum* SF-1502 (root) and *F. proliferatum* AF-04 (onion)	Sesquiterpenoids	**(149)** Cyclonerodiol lactone—C_12_H_20_O_3_/213.148521 [M + H]^+^	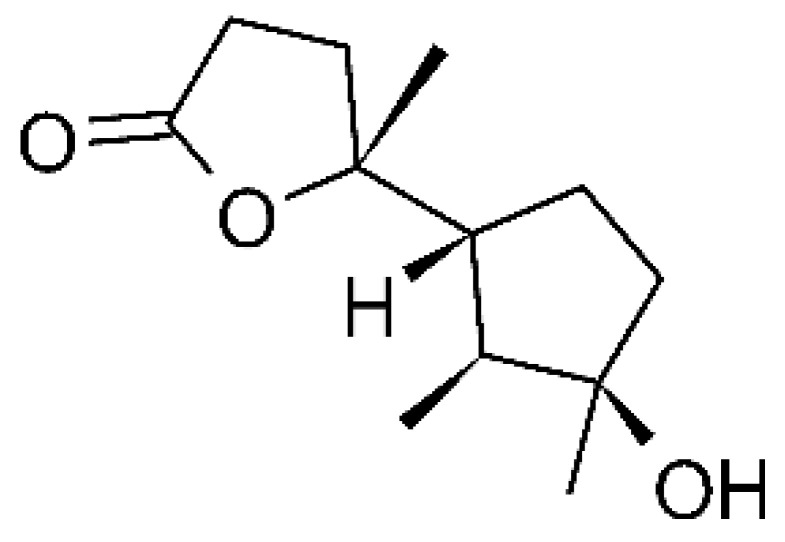	*E. coli*	>100 μg/mL	[55]
*F. proliferatum* AF-04 (onion)	Sesquiterpenoids	**(150)** 3β- hydroxy-β-acorenol—C_15_H_26_O_2_/261.1828 [M+Na]^+^	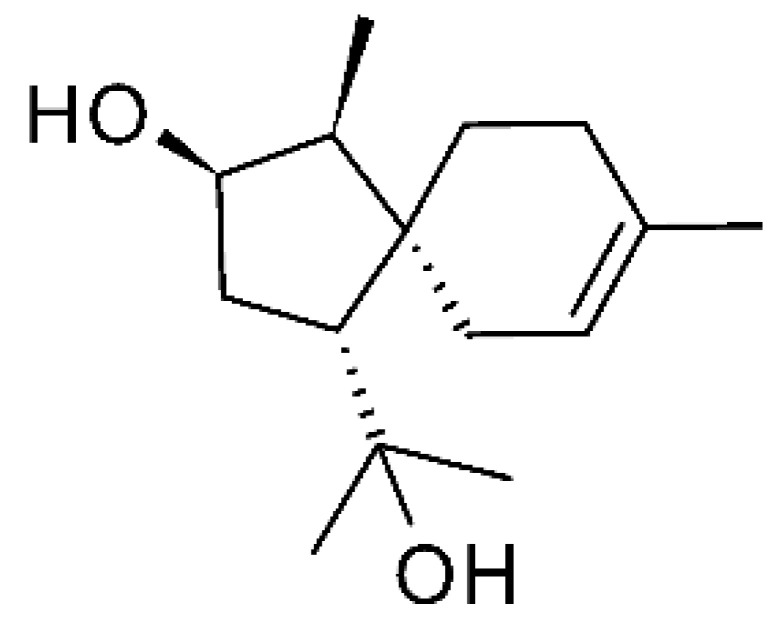	*E. coli*	>100 μg/mL	[55]
*F. proliferatum* AF-04 (onion)	Sesterterpene	**(151)** Fusaproliferin—C_27_H_40_O_5_/445.294851 [M + H]^+^	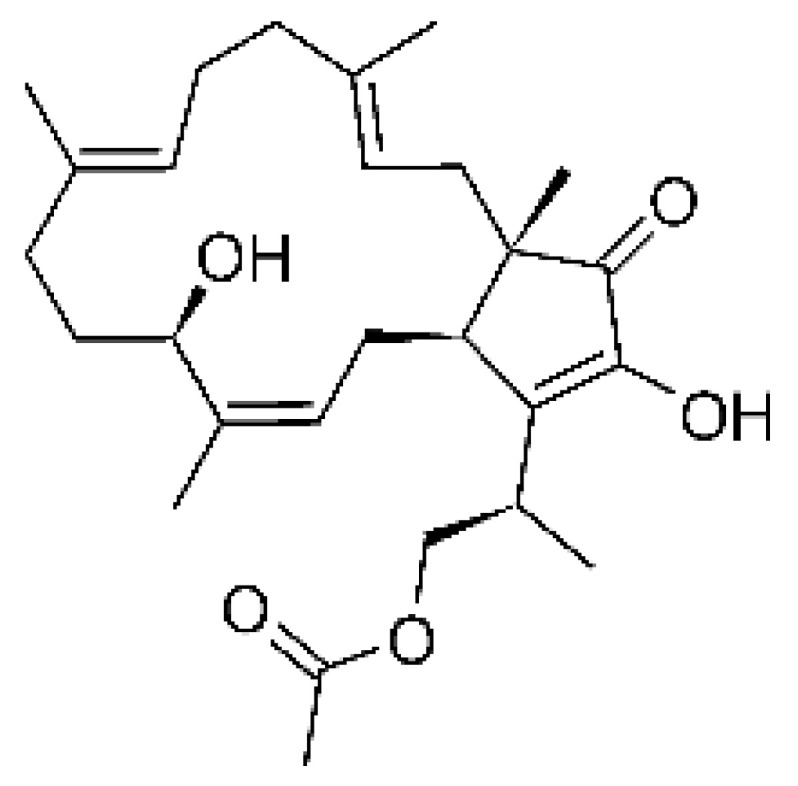	*E. coli*	>100 μg/mL	[55]
*Eupenicillium* sp. LG41 (roots)	Sirenin derivatives	**(152)** Eupenicisirenin A—C_15_H_22_O_3_/251.1638, [M + H]^+^	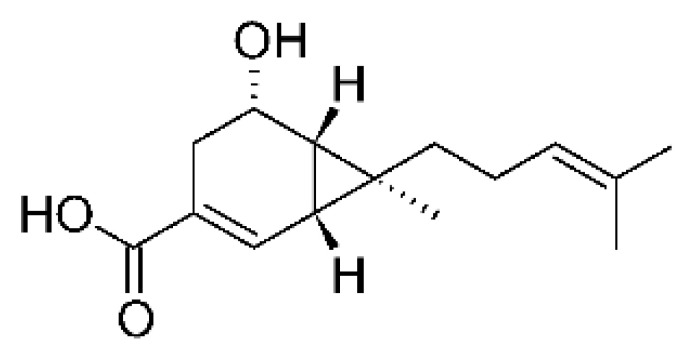	*Acinetobacter* sp. BD4 DSM 586 *Escherichia coli* DSM 1116	>10 μg/mL 10 μg/mL	[71]
*Eupenicillium* sp. LG41 (roots)	Sirenin derivatives	**(153)** Eupenicisirenin B—C_10_H_12_O_4_/195.06 [M−H]^−^	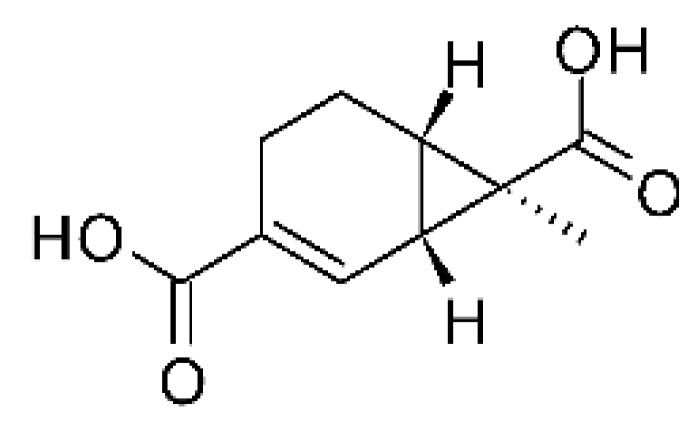	*Acinetobacter* sp. BD4 DSM 586 *E. coli* DSM 1116	5.0 μg/mL 10 μg/mL	[71]
*Diaporthe* sp. LG23 (leaves)	Triterpenoid	**(154)** 19-norlanosta-5(10),6,8,24-tetraene-1α,3β,12β,22S-tetraol—C_29_H_44_O_4_/439.3207, [2M + H]^+^	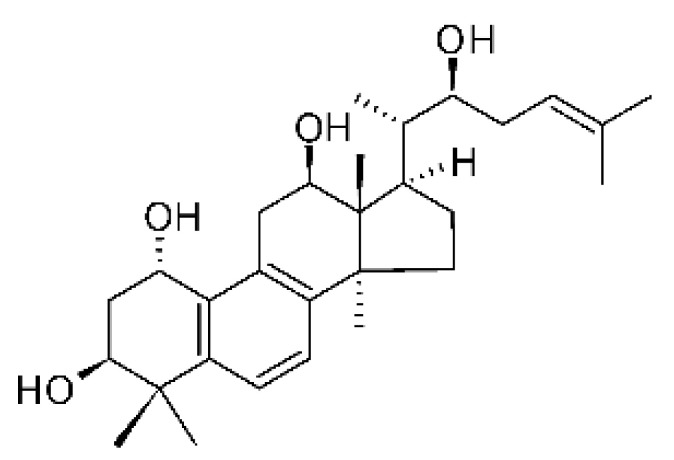	*E. coli* DSM 682 *P. aeruginosa* DSM 22644	5.0 μg/mL 2.0 μg/mL	[75]
*Nigrospora* sp. MA75 (stem)	Xanthones	**(155)** 3,6,8-trihydroxy-1-methylxanthone—C_14_H_12_O_5_/261.07575 [M + H]^+^	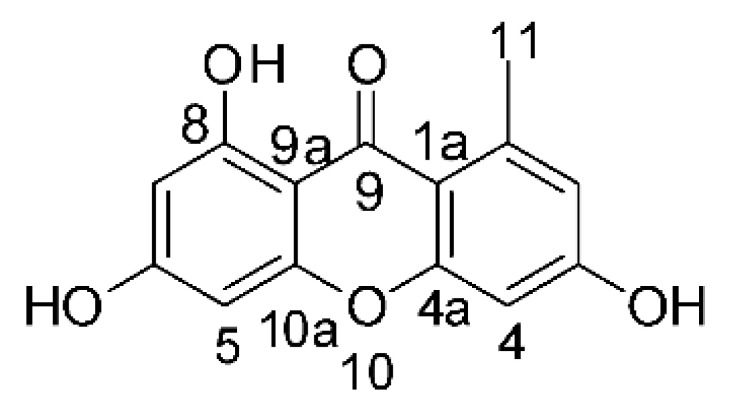	*E.coli*	32 μg/mL	[61]
*Phomopsis* sp. HNY29-2B	α-pyrone	**(156)** Phomopyrone A—C_11_H_14_O_4_/210.0890 [M]^+^	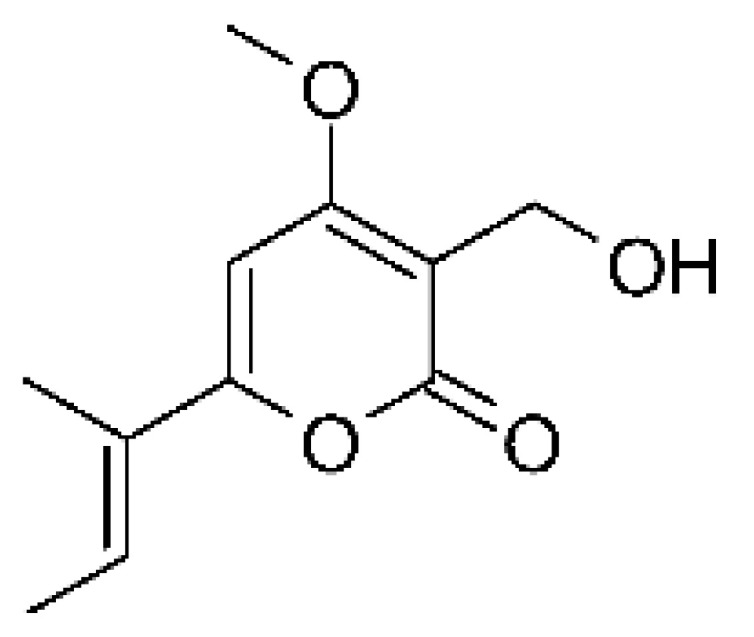	*P. aeruginosa* ATCC 9027	>100 μg/mL	[91]
*Phomopsis* sp. HNY29-2B	α-pyrone	**(157)** Acropyrone—C_11_H_12_O_5_/225.07575 [M + H]^+^	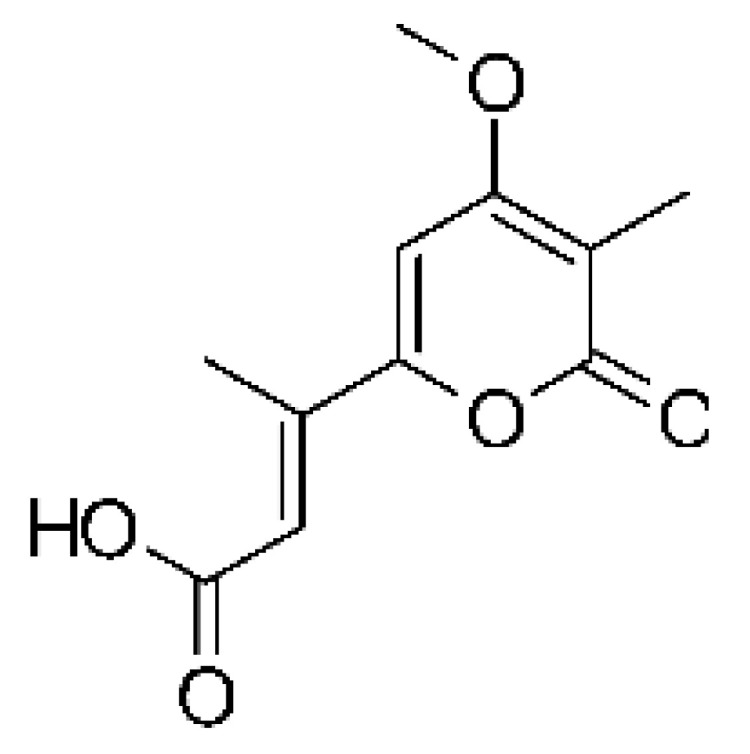	*P. aeruginosa* ATCC 9027	50 μg/mL	[91]
*Phomopsis* sp. HNY29-2B	α-pyrone	**(158)** Ampelanol—C_16_H_20_O_8/_341.123094 [M + H]^+^	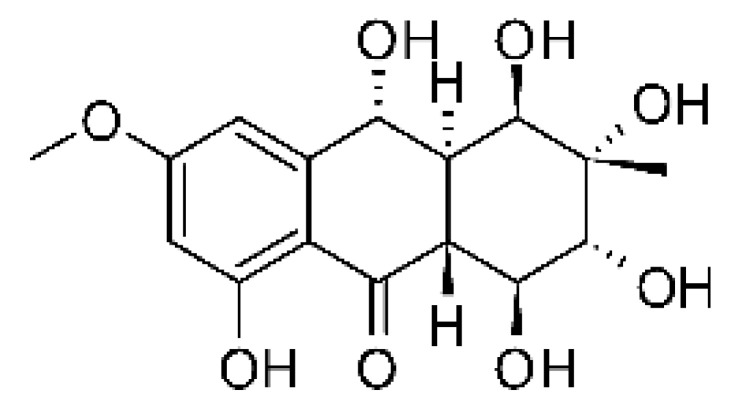	*P. aeruginosa* ATCC 9027	>100 μg/mL	[91]
*Penicillium ochrochloronthe* MPT-163 (roots)	*α*-pyrone derivatives	**(159)** 6-(2′*R*hydroxy-3′*E*,5′*E*-diene -1′-heptyl)-4-hydroxy-3-methyl -2H-pyran-2-one—C_13_H_16_O_4_/ 259.0941 [M+Na]^+^	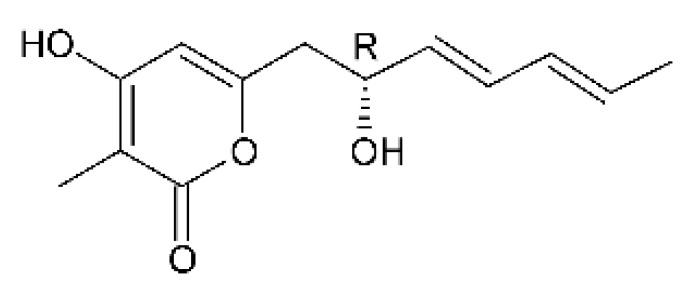	*Enterobacter aerogenes* *E. coli* *P. aeruginosa* *Salmonella enterica* *Salmonella typhi*	50 µg/mL 50 µg/mL 50 µg/mL 50 µg/mL 50 µg/mL	[92]
*Penicillium ochrochloronthe* MPT-163 (roots)	*α*-pyrone derivatives	**(160)** 6-(2′*S*-hydroxy-5′*E*-ene-1′-heptyl)-4-hydroxy-3-methyl-2H-pyran-2-one—C_13_H_18_O_4_/261.1098 [M+Na]^+^	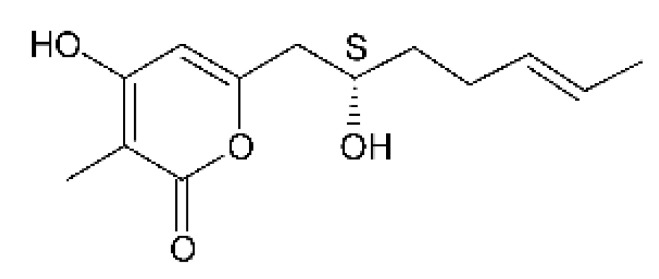	*E. aerogenes* *E. coli* *P. aeruginosa* *S. enterica* *S. typhi*	100 μg/mL 50 μg/mL 50 μg/mL 50 μg/mL 25 μg/mL	[92]
*Penicillium ochrochloronthe* MPT-163 (roots)	*α*-pyrone derivatives	**(161)** 6-(2′*S*-hydroxy-1′-heptyl)-4—hydroxy-3-methyl-2Hpyran-2-one—C_13_H_20_O_4_/263.1255 [M+Na]^+^	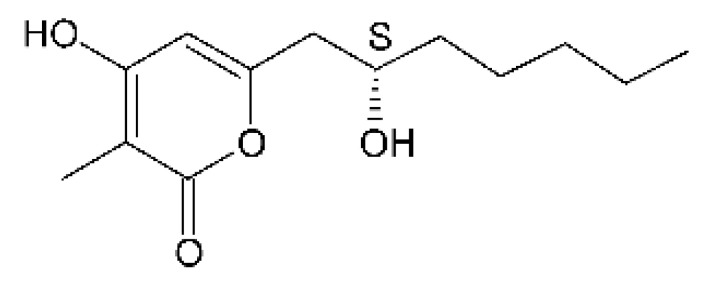	*E. aerogenes* *E. coli* *P. aeruginosa* *S. enterica* *S. typhi*	50 μg/mL 50 μg/mL 50 μg/mL 50 μg/mL 50 μg/mL	[92]
*Penicillium ochrochloronthe* MPT-163 (roots)	*α*-pyrone derivatives	**(162)** trichodermic acid—C_19_H_28_O_3_/305.211121 [M + H]^+^	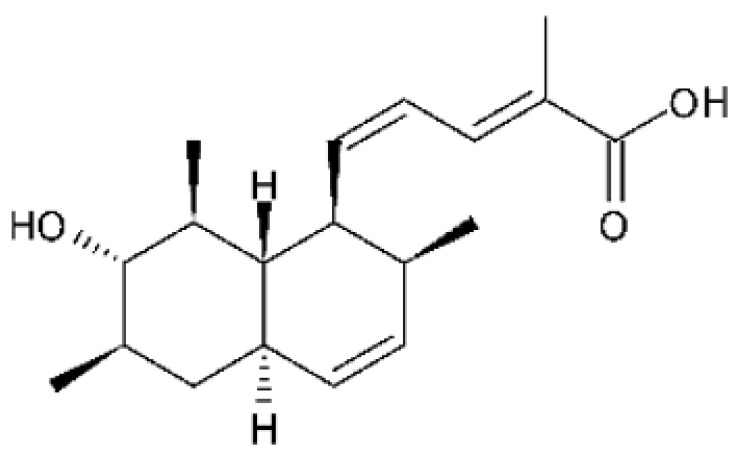	*E. aerogenes* *E. coli* *P. aeruginosa* *S. enterica* *S. typhi*	50 μg/mL 50 μg/mL 50 μg/mL 25 μg/mL 25 μg/mL	[92]
*Stemphylium* sp. 33231 (leaves)	α-pyrone derivatives	**(163)** Infectopyrone A—C_14_H_16_O_6_/303.0840 [M+Na]^+^	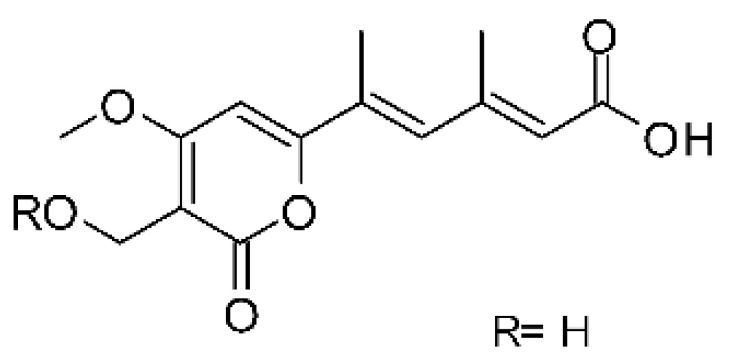	*E. coli* ATCC 25922	2.5 μg/mL	[93]
*Stemphylium* sp. 33231 (leaves)	α-pyrone derivatives	**(164)** Infectopyrone B—C_15_H_18_O_6_/317.0992 [M+Na]^+^	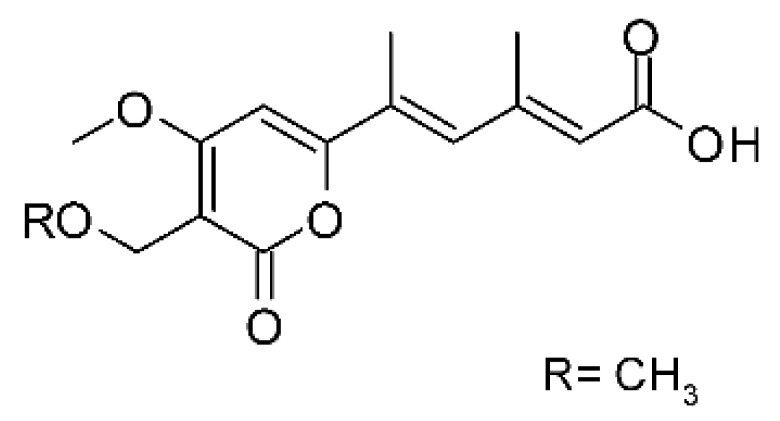	*E. coli* ATCC 25922	2.5 μg/mL	[93]
*Fusarium solani* HDN15-410 (root)	γ-pyrones derivatives	**(165)** Fusolanones A—C_16_H_24_O_3_/265.179821 [M + H]^+^	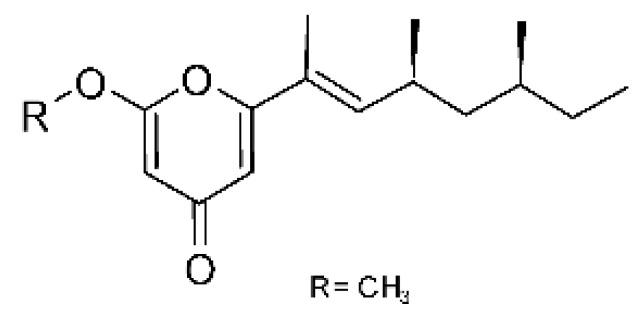	*P. aeruginosa* *V. parahaemolyticus*	26.4 µg/mL >200 µg/mL	[76]
*Fusarium solani* HDN15-410 (root)	γ-pyrones derivatives	**(166)** 6 fusolanones B—C_15_H_22_O_3_/251.164171 [M + H]^+^	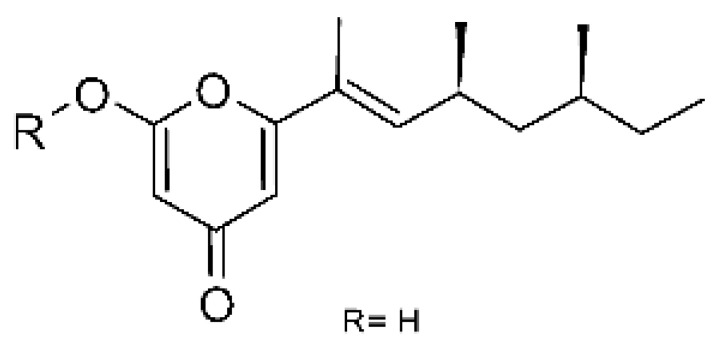	*P. aeruginosa* *V. parahaemolyticus*	12.5 µg/mL 6.25 µg/mL	[76]

* some results of antibacterial activity are represented in the form of halos in millimeters.

**Table 2:** MIC values of the main commercially known antimicrobial agents. Adapted from Clinical and Laboratory Standards Institute (CSLI) [126].

Antibiotics	MICs (µg/mL)			
	Enterobacteriaceae Except *Salmonella* spp.	*Salmonella* spp.	*Pseudomonas aeruginosa* ATCC 27853	*Acinetobacter* spp.
Ampicillin	≤8			
Amoxicillin-clavulanate	≤8/4			
Ceftazidime	≤8/4		≤8	≤8
Imipenem	≤1		≤2	≤2
Meropenem	≤1		≤2	≤2
Doripenem			≤2	≤2
Gentamicin	≤4		≤4	≤4
Azithromycin	≤16			
Tetracycline	≤4			≤4
Ciprofloxacin	≤1	≤0.06		≤1
Levofloxacin	≤2	≤0.12		≤2
Norfloxacin	≤4			
Nalidixic acid	≤16			
Chloramphenicol		≤8		
Piperacycline			≤16	≤16
Ceftazidime-avibactam			≤8/4	
Aztreonam			≤8	
Polymyxin B			≤2	≤2

## Data Availability

Not applicable.
